# Impact of Linker Composition on VHL PROTAC Cell Permeability

**DOI:** 10.1021/acs.jmedchem.4c02492

**Published:** 2024-12-18

**Authors:** Yordanos Esubalew Abeje, Lianne H. E. Wieske, Vasanthanathan Poongavanam, Stefanie Maassen, Yoseph Atilaw, Philipp Cromm, Lutz Lehmann, Mate Erdelyi, Daniel Meibom, Jan Kihlberg

**Affiliations:** † Department of Chemistry—BMC, Uppsala University, Box 576, 75123 Uppsala, Sweden; ‡ Bayer AG, Drug Discovery Sciences, 42113 Wuppertal, Germany

## Abstract

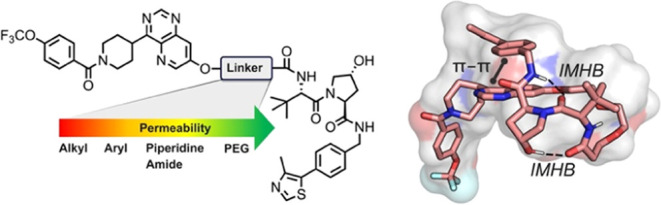

The discovery of cell permeable and orally bioavailable von Hippel-Lindau (VHL) proteolysis targeting chimeras (PROTACs) is challenging as their structures locates them at, or beyond, the outer limits of oral druggable space. We have designed a set of nine VHL PROTACs and found that the linker had a profound impact on passive cell permeability. Determination of the solution ensembles in a nonpolar solvent revealed that high permeability was correlated to the ability of the PROTACs to adopt folded conformations that have a low solvent accessible 3D polar surface area. Our results suggest that the design of cell permeable VHL PROTACs could focus on linkers that facilitate shielding of polar surface area in the VHL ligand in a nonpolar but not in a polar environment. In addition, we found that not only intramolecular hydrogen bonds, but also NH–π and π–π interactions contribute to the stabilization of low-polarity conformations, and thereby to high cell permeability.

## Introduction

Induction of complex formation between proteins has the potential to modulate a large number of biological processes, thereby providing a major increase of druggable target space.^1^ Heterobifunctional compounds that induce the degradation of target proteins by the proteasome or in lysosomes have recently emerged as innovative chemical modalities that capitalize on this opportunity. Proteolysis targeting chimeras (PROTACs) are such heterobifunctional compounds consisting of a ligand for the target protein of interest (POI) connected via a linker to another ligand that binds to an E3 ubiquitin ligase.^2^ PROTAC induced ternary complex formation with the POI and the E3 ligase results in ubiquitination and the subsequent degradation of the POI by the proteasome. Most PROTACs are based either on a Cereblon (CRBN) or a von Hippel-Lindau (VHL) E3 ligase ligand,^3^ with CRBN based PROTACs dominating among those that have entered clinical trials.^4^

Because of their heterobifunctional structure, PROTACs reside in the chemical space beyond the rule of 5 (bRo5),^5−7^ i.e., beyond that defined by the guidelines of Lipinski′s rule of 5 (Ro5)^8^ and Veber′s^9^ rule. Moreover, most PROTACS are found close to or outside the outer borders of orally bioavailable bRo5 space, derived by analysis of drugs, clinical candidates and compounds in lead optimization.^10,11^ Consequently, it is challenging to discover orally bioavailable PROTACs and parenteral administration may be preferred for many of them.^12^ However, CRBN PROTACs are located somewhat closer to the chemical space defined by Lipinski′s and Veber′s rules than VHL PROTACs and thus carry less risks of not achieving oral exposure.^4−7^

Currently, the discovery of orally bioavailable PROTACs often involves the optimization of the molecular descriptors of Lipinski′s and Veber′s rule for the three parts of the PROTACs and thereby also of the overall compound.^13−15^ Commonly used ligands for VHL consume approximately twice as much of the “descriptor budget” available for oral PROTACs as ligands for CRBN, which emphasizes that the “budget” remaining for the POI ligand and the linker is particularly limited for VHL PROTACs.^15^ To a large extent, the structure and properties of the POI ligand are determined by the properties of the binding site on the POI, emphasizing the need for judicious choice and optimization of the linker of VHL PROTACs.^13,16−18^ For example, the replacement of an amide by an ester for coupling of the linker to the POI removes a hydrogen bond donor (HBD) and was found to improve the cell permeability for a series of VHL PROTACs.^19^ In addition, linkers that facilitate for PROTACs to behave as molecular chameleons that adopt folded, less polar conformations in a nonpolar environment and extended, more polar conformations in an aqueous environment may be an essential feature that allows oral VHL PROTACs to balance cell permeability and aqueous solubility.^20,21^

Herein we describe a series of nine VHL PROTACs that vary in the structure of their linkers, but have identical POI and VHL ligands, to obtain insight into how the linker may be used to optimize passive cell permeability, a prerequisite for oral bioavailability. By correlating the cell permeability of the PROTACs to 3D descriptors of size and polarity for the experimentally determined conformational ensembles of five of them we conclude that linkers that allow folding and reduction of polarity, in particular of the VHL ligand, provide PROTACs with high cell permeability. For this series of PROTACs, different types of intramolecular interactions (intramolecular hydrogen bonds, NH–π and π–π interactions) as well as steric shielding of polar groups, and not only intramolecular hydrogen bonds, were all important for the reduction of polarity.

## Results and Discussion

### Design and Characterization of PROTACs

**Figure 1:**
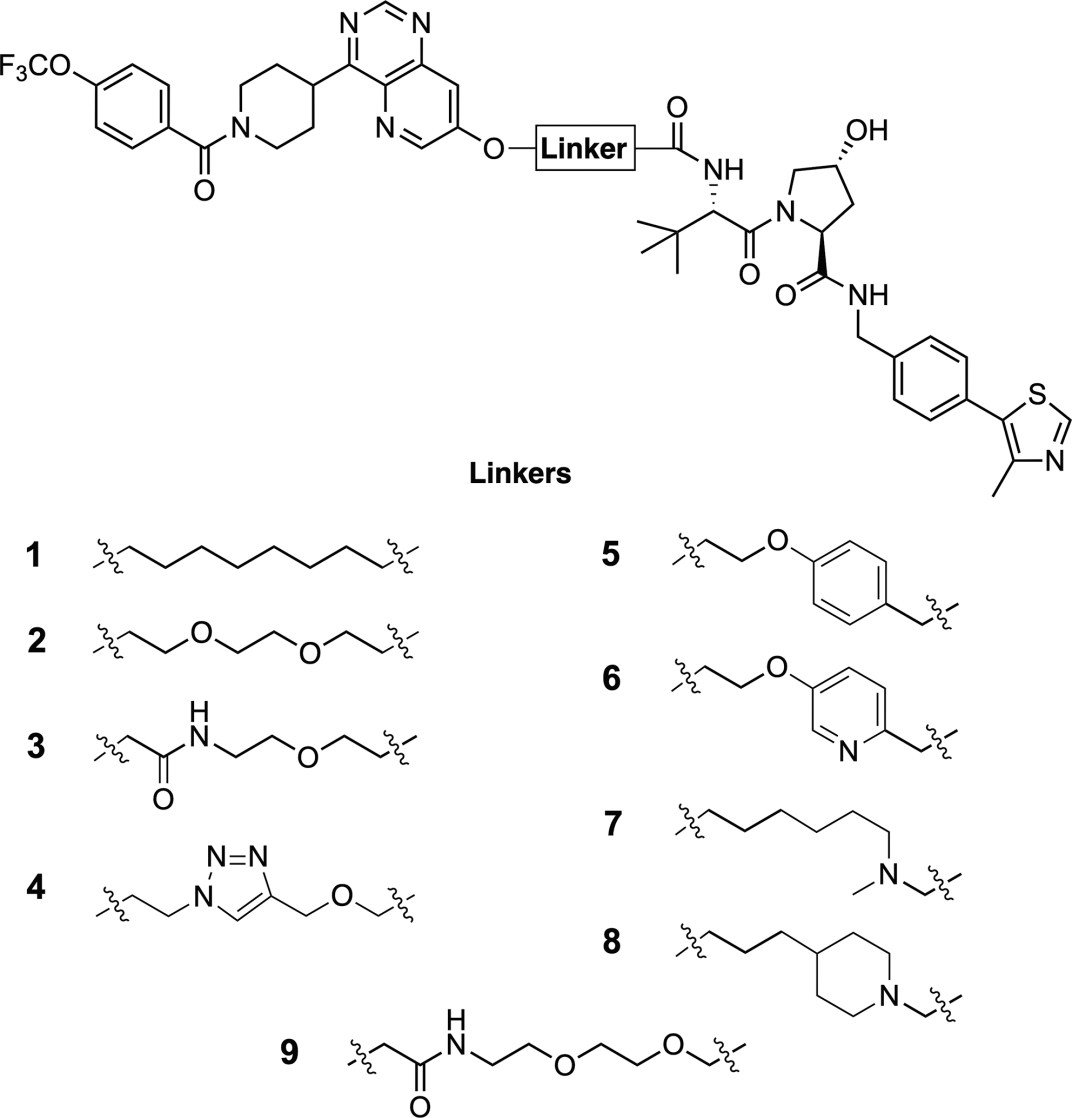
Structures of the designed PROTACs **1**–**9** and their linkers.

PROTACs that have flexible, aliphatic and ethylene glycol-based linkers are often used early in drug discovery projects. More rigid linkers, e.g., ones containing piperazine and piperidine moieties, are usually found in CRBN PROTACs that have undergone lead optimization and which possess oral bioavailability.^4,13^ A recent review found only four VHL PROTACs having some oral bioavailability (>4%), three of which have a flexible aliphatic linker while the fourth has a more rigid linker.^4^ We therefore designed a series of VHL PROTACs having linkers of different chemical composition that varied in flexibility and in polarity to probe the influence of the structure of the linker on cell permeability (Figure 1). PROTACs **1**–**9** all target the extracellular signal-regulated kinase 5 (ERK5)^22^ and recruit the VHL E3 ligase, with **1**–**8** having an eight-atom linker connecting the two ligands. PROTACs **1** and **2** have aliphatic and ethylene glycol-based linkers that are highly flexible, as determined by their number of rotatable bonds (NRotB), i.e., linkers that are often used early in drug discovery projects, but that are also found in oral VHL PROTACs. Then **3** and **4** have a somewhat more rigid linker, while the linkers of **5** and **6** are the most rigid. The polarity of the linkers of **3**–**6** varies, with **5** and **6** being a matched molecular pair in which the pyridine nitrogen atom of **6** may form an intramolecular hydrogen bond (IMHB) with the NH of the amide bond to the VHL ligand. In the matched molecular pair **7** and **8**, PROTAC **8** contains a piperidine moiety, as is commonly found in orally bioavailable CRBN PROTACs, while **7** retains the tertiary amine found in **8** but has a high flexibility, comparable to **1** and **2**. The previously reported^20^ PROTAC **9**, which has an IC_50_ of 1.23 μM for binding to ERK5, has a ten-atom long linker and was included for comparison, in particular to **3** which also contains an amide in its eight-atom linker. At physiological pH, PROTACs **1**–**6** and **9** are all uncharged, while the tertiary amine of **7** and **8** is predominantly uncharged (cf. Physicochemical properties and cell permeability, below).

An analysis of >1800 PROTACs at Arvinas allowed outer limits for the orally bioavailable chemical space of PROTACs to be derived, e.g., that the molecular weight (MW) should be ≤950 Da (Table 1).^15^ These guidelines agree well with those of the bRo5 space obtained by analysis of oral drugs, clinical candidates and compounds in lead optimization.^10,11^ Other studies have found that compounds that approach or surpass the MW guideline <950–1000 Da,^23^ or that have a lipophilicity that deviates from the drug-like optimum (cLogD = 3),^11^ very frequently suffer from low cell permeability, in part explaining their lack of oral bioavailability.^24^ The lipophilicity (cLogP), number of hydrogen bond donors (HBDs) and acceptors (HBAs) of PROTACs **1**–**9** reside within or at the outer limits of the two guidelines (Table 1). In addition, several of them have a MW, topological polar surface area (TPSA) and flexibility, as defined by their NRotB count, just beyond that of the outer limits. In conclusion, the descriptors of **1**–**9** put these PROTACs at high risk of having a low cell permeability and most likely to lack sufficient oral bioavailability.

**Table 1:** Comparison of Descriptors for the Outer Limits of PROTAC and bRo5 Oral Druggable Space with Those Calculated for PROTACs **1**–**9**^a^

PROTAC	MW (Da)	cLogP	HBD	HBA	TPSA (Å^2^)	NRotB
PROTAC, outer limit^b^	950	7	4	15	200	14
bRo5, outer limit^b^	1000	10	6	15	250	20
1	987	7.57	3	11	189	21
2	991	4.21	3	13	207	21
3	1004	3.96	4	13	227	20
4	1014	3.81	3	14	229	19
5	1009	6.41	3	12	198	18
6	1010	5.14	3	13	211	18
7	1002	6.47	3	12	192	22
8	1014	6.41	3	12	192	18
9	1034	3.54	4	14	236	22

a Outer limits of oral druggable space proposed from analysis of PROTACs,^15^ and drugs and clinical candidates in the bRo5 space,^10^ as defined by the descriptors of Lipinski’s rule of 5^8^ and Veber’s rule.^9^

b Descriptors were calculated using MOE (version 2019.01). MW, molecular weight; cLogP, calculated lipophilicity; HBD, number of hydrogen bond donors; HBA, number of hydrogen bond acceptors; TPSA, topological polar surface area; NRotB, number of rotatable bonds.

### Synthesis

**Scheme 1:**
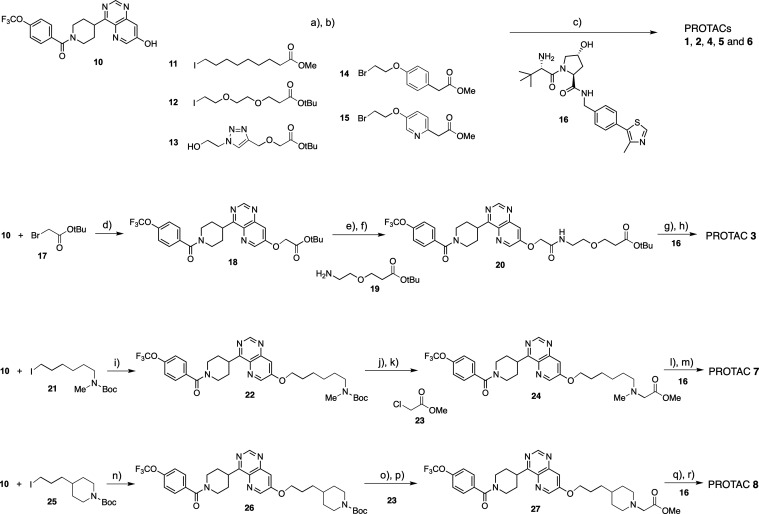
Synthesis of PROTACs **1**–**8** Reagents and conditions. (a) K_2_CO_3_, DMF, 25–80 °C, 2-24 h (for **11**, **12**, **14** and **15**, 82–92%) or Ph_3_P, DIAD, THF, r.t., overnight (for **13**, 91%); (b) LiOH, H_2_O, MeOH, r.t., 2 h (for **11**, **14** and **15**, 81–97%) or TFA, DCM r.t., 3 h (for **12** and **13**, 85-91%); (c) T3P, DIPEA, DMF, 40 °C, overnight, 14–41%; (d) K_2_CO_3_, DMF, 40 °C, overnight, 88%; (e) TFA, DCM r.t., 3 h, 84%; (f) T3P, DIPEA, DMF, r.t., overnight, 92%; (g) TFA, DCM, r.t., 3 h, 80%; (h) T3P, DIPEA, DMF, 40 °C, overnight, 24%; (i) K_2_CO_3_, DMF, r.t., overnight, 64%; (j) HCl, 4 M in 1,4-dioxane, DCM, r.t., 1.5 h, quant., crude; (k) Cs_2_CO_3_, DMF, 60 °C, 5 h, 72%; (l) LiOH, H_2_O, THF, 60 °C, 2 h, quant., crude; (m) HATU, DIPEA, DMF, r.t., overnight, 1%; (n) K_2_CO_3_, DMF, r.t., overnight, 89%; (o) HCl 4 M in 1,4-dioxane, DCM, r.t., 1.5 h, 93%; (p) Cs_2_CO_3_, DMF, 60 °C, 5 h, 78%; (q) LiOH, H_2_O, THF, 60 °C, 2 h, quant., crude; (r) HATU, DIPEA, DMF, r.t., overnight, 2%. DCM = dichloromethane, DIAD = diisopropyl azodicarboxylate, DIPEA = diisopropylethylamine, DMF = *N*,*N*-dimethylformamide, h = hour(s), HATU = *O*-(7-azabenzotriazol-1-yl)-*N*,*N*,*N*′,*N*′-tetramethyluronium hexafluorophosphate, M = molar, MeOH = methanol, Ph_3_P = triphenylphosphine, r.t. = room temperature, T3P = propanephosphonic acid anhydride, TFA = trifluoroacetic acid, THF = tetrahydrofurane.

PROTACs **1**–**8** were synthesized in a modular fashion using well-established chemistry (Scheme 1). Alkylations of the phenolic OH of the POI ligand **10**(22) with different alkyl halides (**11**, **12**, **14**, **15**, **17**, **21**, **25**), or with the alcohol **13** under Mitsunobu conditions, were followed by cleavage of the respective esters or removal of a BOC protecting group in case of amines **22** and **26**. Carboxylic acids resulting from the linker elements of **11**–**15** were then reacted with the commercially available VHL binder **16** under amide coupling conditions to give PROTACs **1**, **2**, and **4**–**6**. The acid resulting from cleavage of the tBu ester **18** was elongated with **19** under amide formation to give **20**. Liberation of the corresponding acid of **20** and coupling with amine **16** yielded PROTAC **3**. Low to moderate yields (14–41%) were obtained in the last step in the syntheses of **1**–**6**, i.e., the amide coupling promoted by propanephosphonic acid anhydride (T3P). No optimization of the amide forming step was done as enough material for the subsequent physiochemical property, biochemical and NMR studies was obtained. Synthesis of PROTACs **7** and **8** also began by introduction of the linker region via an alkylation of the phenolic OH of the POI ligand **10**. After N-deprotection of **22** and **26**, alkylation with **23** gave esters **24** and **27**. Again ester cleavage and amide coupling were performed as the next step, this time to provide **7** and **8**. Yields for the reactions giving **7** and **8** are very low (1–2%) due to unclean conversions giving multiple products, as well as purification issues stemming from a side product coeluting with **7** and **8**. For both PROTACs the coeluting molecule is 16 Da heavier than **7** or **8**, respectively. As enough pure material for the subsequent studies was collected, no further elucidation of the side-products or attempts to optimize the syntheses of **7** and **8** were performed.

### Physicochemical Properties and Cell Permeability

Before determining the cell permeability of PROTACs **1**–**8** we assessed their aqueous solubility. Kinetic solubilities can be measured with high throughput, but it has been pointed out that interpretation of the data for PROTACs is associated with large uncertainties;^25^ thus the use of more biorelevant media has been recommended when obtaining accurate solubilities is essential.^26^ As the kinetic solubility of **9** in PBS at pH 6.5 is low (6.8 μM),^20^ we determined the solubilities of PROTACs **1**–**6** under the same conditions. Solubilities were not determined for **7** and **8** due to the low amounts obtained in their synthesis. PROTACs **1**–**6** also had low solubilities (<1 μM for **1**, **2**, **5**, and **6**, 10–20 μM for **3** and **4**; cf. molecular formulas strings document), therefore microtiter plate wells were inspected visually in the biochemical and cell-based assays to ensure that IC_50_ values were not affected by the precipitation of poorly soluble compounds. As an additional precaution the shape of the inhibition curves was examined carefully.

**Figure 2:**
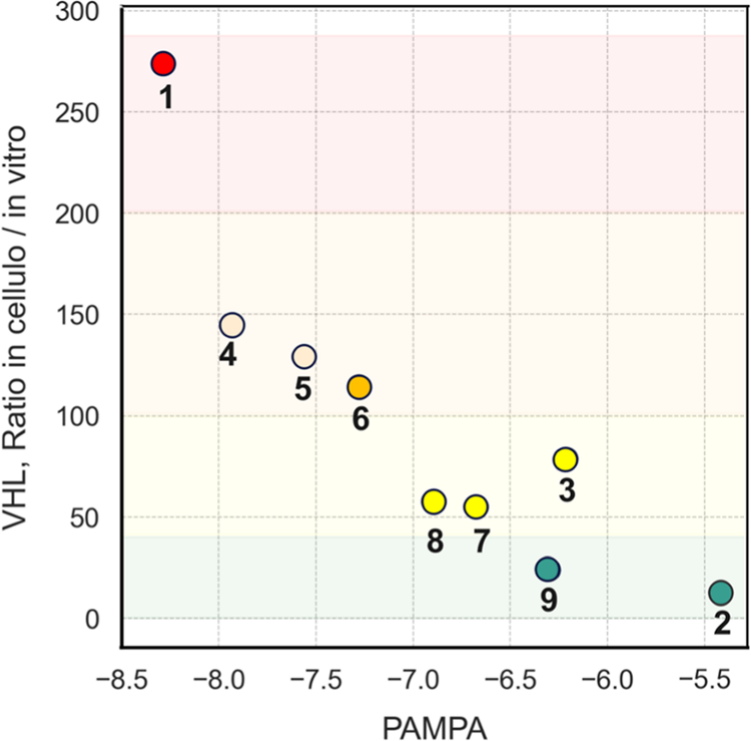
Correlation between the cell permeabilities, i.e., the ratio between the potencies for binding to VHL in a cell-based and in a biochemical assay, and the membrane permeabilities in the PAMPA assay of PROTACs **1**–**9**. The faint red, orange, yellow and green shading indicates the cut-offs for low, medium–low, medium–high and high cell permeability classes, respectively, as determined by the in cellulo/in vitro permeability ratios. The filled circle for each compound has been color coded in the same way. Note that the PAMPA permeabilities of PROTACs **4** and **5** (marked in light orange) are lower than, or equal to the plotted values.

The ratio between the potencies for binding of a PROTAC to VHL in a cell-based and in a biochemical assay has been used as a surrogate measure for its passive permeability into a target cell.^27−29^ PROTACs **1**–**9** were found to show very large differences in this permeability measure, with permeabilities that could be grouped in four classes (Table 2, Figure 2). PROTACs **2** and **9** had a high cell permeability (low in cellulo/in vitro ratio), the permeability of **3**, **7**, and **8** was classified as medium–high, while that of **4**, **5**, and **6** was medium–low and **1** low. Intracellular binding to macromolecules and organelles may affect the in cellulo/in vitro ratio for binding to VHL. Therefore the passive permeabilities of **1**–**9** was also determined in the parallel artificial membrane permeability assay (PAMPA). Also, in this assay PROTACs **1** and **2** were the least and most permeable, respectively, with **2** having a close to 3 orders of magnitude higher permeability than **1** (Table 2, Figure 2). Interestingly, the permeabilities determined in the two assays showed a good qualitative correlation to each other, but with PROTAC **3** having a somewhat higher PAMPA permeability than expected from the in cellulo/in vitro ratio (Figure 2). We recently found that the two assays also correlated in ranking the permeabilities of a pair of CRBN PROTACs, one of which had low and the other high permeability.^30^

**Table 2:** Lipophilicity and Cell Permeabilities for PROTACs **1**–**9**^a^

PROTAC	LogD (pH 7.5)	VHL (in cellulo) IC_50_ (μM)^a^	VHL (in vitro) IC_50_ (μM)^a^	VHL, ratio in cellulo/in vitro^b^	permeability class^c^	PAMPA Log[*P*_e_ (cm/s)]^d^
1^e^	3.8	43.0 ± 4.6	0.157 ± 0.0067	274	L	–8.29 ± 0.023
2^e^	2.9	8.92 ± 0.80	0.700 ± 0	12.7	H	–5.42 ± 0.035
3^e^	2.7	42.2 ± 2.7	0.533 ± 0.033	79.1	M–H	–6.21 ± 0.046
4	2.7	17.8 ± 2.8	0.123 ± 0.0033	145	M–L	<−7.94 ± 0.20
5	3.2	20.2 ± 4.9	0.257 ± 0.0067	129	M–L	<−7.56 ± 0.30
6^e^	3.0	16.9 ± 1.9	0.147 ± 0.0033	115	M–L	–7.28 ± 0.035
7^e^	3.7	34.0 ± 6.3	0.617 ± 0.017	55.1	M–H	–6.67 ± 0.017
8	3.6	35.3 ± 3.9	0.610 ± 0.021	57.8	M–H	–6.90 ± 0.061
9^e^^,^^f^	2.7	7.52 ± 0.77	0.313 ± 0.0033	24	H	–6.30 ± 0.074

a The potencies for binding to VHL are mean values ± SEM from three and six repeats in the biochemical (in vitro) and cell-based (in cellulo) assays, respectively. Descriptions of both assays are provided in the Experimental Section.

b Highly permeable PROTACs have a low in cellulo/in vitro ratio, while the opposite is true for low permeable ones.

c Cut-offs used in this work for the in cellulo/in vitro permeability ratios: H = high (ratio < 40), M–H = medium–high (ratio 41–100), M–L = medium–low (ratio 101–200), L = low (ratio > 200).

d Data from the parallel artificial membrane permeability assay (PAMPA). Data are mean values ± SEM from three, six or nine repeats.

e Conformational ensemble determined in CDCl_3_.

f Permeability data differ somewhat from those reported previously^20^ due to slight differences in protocols for the VHL binding and PAMPA assays.

Conflicting results have been reported regarding the suitability of the PAMPA assay for assessment of the cell permeability of PROTACs.^26^ While the PAMPA assay has been used for understanding and improving the passive permeability within series of PROTACs,^31^ PAMPA permeabilities were found not to correlate to permeabilities across Lilly Laboratories Cell-Porcine Kidney 1 (LLC-PK1) cell monolayers.^25^ The lack of correlation may originate, at least to some extent, from that LLC-PK1 cells overexpress the efflux transporter MDR1 (P-glycoprotein 1, Pgp), while only passive permeability is determined in the PAMPA assay. The good correlation between PAMPA permeabilities and the in cellulo/in vitro ratio reported herein can be understood from that the cells used for the determination of the ratio express no, or only low levels of efflux transporters,^32^ i.e., both assays determine passive permeability.

The linkers of PROTACs **7** and **8** contain a tertiary amine, the p*K*_a_ of which was calculated with the SimPlus^33^ tool to understand if it was charged or not at physiological pH. The p*K*_a_ of **8** was predicted to be 6.22, indicating **8** to be uncharged at pH 7.4 (94% uncharged), while that of **7** was predicted to be 7.16, i.e., closer to physiological pH. Determination of the p*K*_a_ of **7** revealed it to be 6.90 and thereby that **7** is predominantly uncharged (76%) at pH 7.4. Both compounds were therefore treated as being mainly uncharged in the subsequent analyses, and the uncharged form of **7** was used in the NMR studies of its conformational ensemble in chloroform.

The MW and HBD counts show little variation between PROTACs **1**–**9**, whereas HBA counts, TPSA and NRotB vary somewhat more, and cLogP varies most (Table 1). Only very weak correlations were found between each of the two permeability measures of the PROTACs and these six calculated descriptors (Figures S1 and S2). Similarly, no clear trends were found between the permeabilities and a combination of lipophilicity (cLog P) and polarity (TPSA) (Figure S3). These observations are not entirely unexpected since all 17 descriptors, including the six used herein, were found to be of similar importance in the construction of machine learning models for PROTAC cell permeability.^29^

cLogP is the descriptor that varies the most between the nine PROTACs, with values ranging from 3.5 for **9** to 7.6 for **1**. However, predictions of *c *Log* P* may show large variations depending on the method used and correlations to experimental values are often poor.^34,35^ The chromatographic lipophilicities of the nine PROTACs were therefore determined to understand to what extent the cell permeability differences may originate from lipophilicity. The lipophilicities of **1**–**9** ranged from 2.7 to 3.8 (Table 2), i.e., were within one logarithm unit of 3, which is usually considered as optimal for cell permeable and orally absorbed drugs. For **1**–**9**, LogD values showed a strong linear correlation to *c* Log *P* (*R*^2^ = 0.91), but *c* Log *P*s overestimated lipophilicities by 1–3.8 orders of magnitude (Figure S4). PROTACs **1**, **7**, and **8** had LogDs at the high end of the range, but while **1** had the lowest permeability **7** and **8** fell in the medium–high range. Moreover, the LogD of the least and most permeable of the set of PROTACs, i.e., **1** and **2**, only differed by 0.9 units. Thus, while lipophilicity may contribute to the differences in cell permeability other properties must be more important. Recently, the ability of three CRBN PROTACs,^30^ as well as several drugs and other compounds in the bRo5 space,^21^ to behave as molecular chameleons which adopt conformations that have low surface accessible polar surface area (PSA) and a low radius of gyration (*R*_gyr_) has been correlated to differences in cell permeability. We therefore proceeded to determine the conformational ensembles of some of PROTACs **1**–**9** using solution-phase NMR spectroscopy to understand whether conformational preferences are the root-cause of the large differences in cell permeability displayed by these PROTACs.

### Determination of Conformational Ensembles

As described in two studies based on solution-phase NMR spectroscopy, PROTACs are flexible compounds that exist in an equilibrium of rapidly exchanging conformations.^20,30^ We therefore used the NMR analysis of molecular flexibility in solution (NAMFIS) algorithm^36^ to deconvolute the time averaged NMR data into solution ensembles for the selected PROTACs using procedures that have been reported recently.^30,37^ The NAMFIS algorithm was chosen as it is available open-source, showed excellent performance in a recent comparison of deconvolution algorithms,^38^ and has been validated in numerous studies of structurally diverse compounds ranging from small molecules to larger macrocycles and a few PROTACs (cf. ref (37), and references therein). In brief, NAMFIS fits interproton distances, determined by the nuclear Overhauser effect (NOE),^39^ and dihedral angles derived from the ^3^*J*_HH_ scalar coupling constants,^40,41^ to back-calculated data from a combination of conformations selected from a theoretical ensemble.^36^ Theoretical conformational ensembles that provide a comprehensive coverage of conformational space are crucial for the successful determination of solution ensembles.^42^ Unrestrained Monte Carlo conformational searches using a high energy cutoff, different force fields and implicit solvation models, followed by removal of redundant conformations are used to this end. Finally, the solution ensembles obtained by using the NAMFIS algorithm are validated by comparison of the experimentally observed interproton distances and dihedral angles to those back-calculated from the solution ensembles, by the addition of ±10% random noise to the experimental data, and by systematic removal of individual experimental restraints.

We selected one uncharged PROTAC from each of the four permeability classes (Table 2) for the NMR studies, i.e., **1** (Low), **2** (High), **3** (Medium–High) and **6** (Medium–Low). A slow conformational exchange was observed for **8** (Medium–High), that contains a piperidine moiety in the linker, which prevented the determination of its conformational ensemble. Instead, **7** which has a permeability that is almost identical to that of **8** (Table 2) was included as a PROTAC that contains a basic tertiary amine in the linker. Since compounds are, in general, assumed to cross membranes in their uncharged form, the tertiary amine of **7** was unprotonated in the NMR studies, as revealed by the shift of the *N*-methyl group (δ_H_: 2.22 ppm, δ_C_: 41.8 ppm). Chloroform was used as solvent for the five PROTACs since it has a dielectric constant (ε = 4.8) close to that determined for the central, lipidic part of a cell membrane (ε = 3.0).^43^ The solution ensemble of PROTAC **9** in chloroform has been reported previously,^20^ and can be used for comparisons with **1**, **2**, **3**, **6**, and **7**.

**Figure 3:**
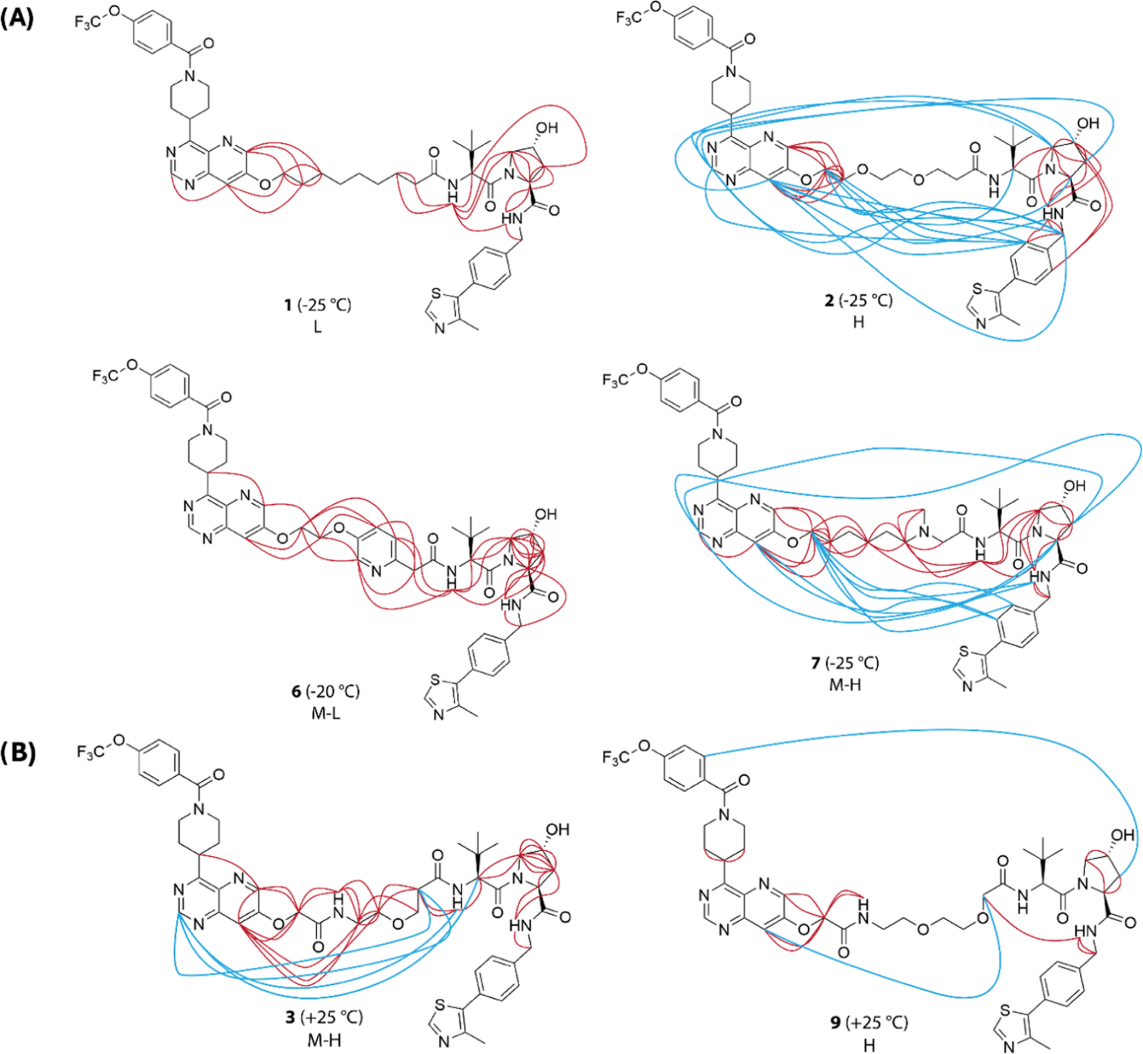
Overview of the experimentally determined nuclear Overhauser effects, that were used in the NAMFIS analysis of PROTACs **1**–**3**, **6**, **7**, and **9**, determined (A) at low temperature (−20 or −25 °C) and (B) at room temperature (+25 °C). The temperature used to record the NMR spectra and the permeability class (cf. Table 2) is given for each PROTAC. Blue lines indicate long-range NOEs between protons in the ERK5 and VHL ligands, or between protons in one of the ligands and protons at the other end of the linker. All other NOEs are indicated by red lines. The conformational ensemble of **9** has been reported previously.^20^

Spectra were first acquired at low temperature (−25 or −20 °C) for the five PROTACs to obtain optimal chemical shift dispersion, thereby minimizing overlap between signals. Low-permeable PROTAC **1** only showed NOEs between protons located close to each other, i.e., between protons in the ERK5 ligand and adjacent parts in the linker as well as between protons within the VHL ligand and between some of these protons and the adjacent linker (Figure 3A). The lack of long- and medium-range NOEs for **1** indicates that it adopts elongated instead of folded conformations in chloroform. In contrast, the high- and high-moderate-permeable PROTACs **2** and **7**, respectively, display several long-range NOEs between protons in the two ligands, or between the VHL ligand and protons in the linker that are close to the ERK5 ligand, indicating that **2** and **7** adopt folded conformations in solution. PROTAC **6** shows a different pattern of NOEs, most of which are between protons close in the chemical structure. Interestingly, these short-range NOEs bridge from the ERK5 ligand across the linker to the VHL ligand. From this pattern, it appears likely that PROTAC **6** could adopt less folded and more elongated conformations than **2** and **7**.

Unfortunately, the NMR spectra revealed that medium–high-permeable PROTAC **3** populated two sets of conformations in slow exchange at −25 °C, which prevented the determination of its conformational ensemble at low temperature due to severe overlap of the proton resonances. Spectra were instead recorded for **3** at +25 °C with the aim of determining an ensemble that could be compared to the one reported previously for high-permeable **9** at this temperature.^20^ At +25 °C, all conformations of **3** were in fast exchange and several medium- or long-range NOEs were observed between protons in either of the ligands and protons in the linker. Additionally, long-range NOEs were found between protons in the *tert*-butyl glycine moiety of the VHL ligand and in the ERK5 ligand (Figure 3B). This may indicate that **3** populates a mixture of folded, semifolded and elongated conformations at room temperature. The conformational ensemble of **9**, which displayed long- and medium-range NOEs, has been reported to consist mainly of folded conformations.^20^

As mentioned above the lack of long- and medium-range NOEs for **1** prevented the determination of its solution conformational ensemble and thereby led to uncertainty in comparisons to the other PROTACs. Another limitation of this study is the lack of reliable NOEs that define the orientation of the distal half of the ERK5 ligand, and for the thiazole of the VHL ligand, with regards to the rest of the molecule in PROTACs **1**, **2**, **3**, **6**, and **7** (Figure 3). Since both poorly defined moieties contain few rotatable bonds, i.e., they are rather rigid, conformations selected by the NAMFIS analyses were used for the further property and structural analyses without attempts to optimize them by energy minimization. Up to approximately 1000 conformations may be used as input for the NAMFIS algorithm. For PROTACs **2**, **3**, **6**, and **7**, theoretical ensembles consisting of from 98 to 507 conformations were used (Table S11). These ensembles were obtained when a root-mean-square deviation (RMSD) cutoff of 3.0 Å for heavy atoms was used for elimination of redundant conformations in the theoretical conformational ensemble of each PROTAC. Consequently, the conformational ensembles obtained as output from NAMFIS for these four PROTACs may be described as having been determined with a medium resolution.

### Correlating Conformational Ensembles to Permeability

The solution ensembles determined in this work for VHL PROTACs **2**, **3**, **6**, and **7**, and the one reported previously for **9**, were described by a number of conformations that ranged from six for **6** to 13 and 14 for **2** and **7**, respectively (Table 3). The number of major conformations that had a population ≥10% were similar in the ensembles, i.e., either three or four. The large difference in the number of conformations populated by **2** and **7** as compared to **6**, all of which were studied at low temperature, is an indicator that **6** is more rigid than **2** and **7**. Most likely the pyridine moiety in the linker of **6** reduces the flexibility of this PROTAC, as also indicated by its reduced NRotB count (Table 1). By a similar reasoning the longer linker of PROTAC **9** would be expected to result in that **9** is more flexible than **3**, which has a shorter linker. However, this was not found to be the case.

**Table 3:** Population of the Conformations in the Solution Ensembles of PROTACs **2**, **3**, **6**, **7**, and **9**

	conformation number, population (%)													
PROTAC^a^	1	2	3	4	5	6	7	8	9	10	11	12	13	14
**2** (H)^b^	21	16	11	11	9	8	6	6	3	3	2	2	2	
**6** (M–L)^b^	45	18	17	9	8	3								
**7** (M–H)^b^	23	14	14	12	7	7	5	5	3	2	2	2	2	2
**3** (M–H)^c^	24	16	15	13	6	5	5	4	4	3	3	2		
**9** (H)^c^^,^^20^	33	22	16	11	8	4	3	3						

a The permeability class is given in parentheses for each PROTAC.

b Ensemble determined at −20 or −25 °C.

c Ensemble determined at +25 °C.

The permeability of a compound across a cell membrane is determined by the size and polarity of the conformations crossing the membrane, with increased values for both properties resulting in lower permeabilities.^44^ The solvent accessible 3D polar surface area (SA 3D PSA) is a 3D descriptor of polarity found to correlate better to passive, transcellular permeability than the TPSA for compounds in the bRo5 space.^44,45^ Similarly, size is approximated better by the radius of gyration^46^ (*R*_gyr_) than by molecular weight.^44^

**Figure 4:**
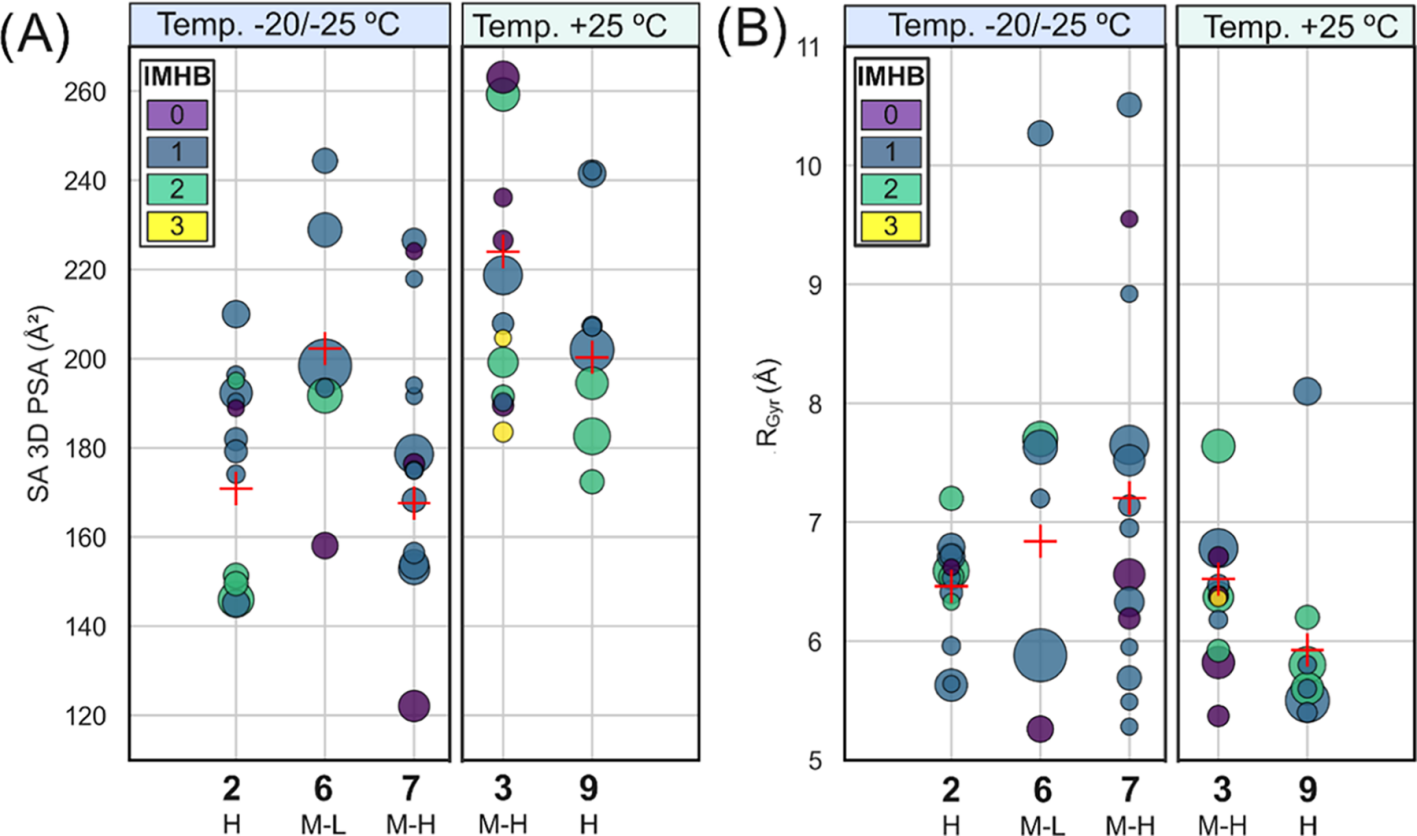
(A) Solvent accessible 3D polar surface area (SA 3D PSA) and (B) radius of gyration (*R*_gyr_) for the solution ensembles in CDCl_3_ of PROTACs **2**, **6**, and **7** at −20 or −25 °C, and of PROTACs **3** and **9**(20) at +25 °C. The permeability class (cf. Table 2) is given below the number of each PROTAC. The area of each circle is proportional to the population (in %) of the corresponding conformation. The number of intramolecular hydrogen bonds (IMHB) in each conformation is indicated by the color coding. Population weighted mean values are shown as red plus signs.

The large differences in size (*R*_gyr_) and polarity (SA 3D PSA) between the conformations in the ensembles of the PROTACs investigated herein illustrate the high flexibility of this series (Figure 4A,4B). Differences are particularly large for PROTAC **7**, which has a tertiary amine in its linker. It can be assumed that cell permeability is not driven by a single or a few of the conformations populated by these flexible compounds. Consequently, we investigated how the population weighted mean values of *R*_gyr_ and SA 3D PSA, i.e., the size and polarity of the overall ensemble, correlated to membrane permeability. As described by these two 3D descriptors, the ensemble of the highly permeable **2** was on average both smaller in size and less polar than that of the medium–low permeable **6** (Figure 4). Similarly, the ensemble of the highly permeable **9** was smaller and less polar than that of medium–high permeable **3**. Thus, the 3D descriptors calculated for the solution ensembles of these two pairs of PROTACs provide an excellent qualitative rationalization of the observed differences in permeability within each pair.

Attempted rationalization of the permeability of **7** (medium–high) in comparison to **2** (high) and **6** (medium–low) was not straightforward since the ensemble of **7** has a lower population weighted SA 3D PSA than **2** and **6**, while **7** has the highest *R*_gyr_; i.e., the two descriptors indicated opposite rankings of the permeability of **7**. The comparison of **7** with **2** and **6** is further complicated by the presence of a tertiary amine in **7** (p*K*_a_ 6.90). Thus, **7** carries a partial positive charge at physiological pH that is expected to reduce its permeability as compared to what is indicated by the two descriptors, which were calculated for the ensemble of the uncharged form of **7** used in the NMR studies. Taken together, the contributions of size, polarity and partial charge may provide a rationale for **7** having a permeability intermediate between that of **2** and **6**.

A few of the conformations in the ensembles of PROTACs **6** and **7** have an elongated, linear shape characterized by a *R*_gyr_ > 8 Å (Figures S7 and S8, Table S18). The SA 3D PSA of these linear conformations is high (>215 Å^2^) since polar groups are not shielded from the surrounding environment by intramolecular interactions. It is therefore reasonable to hypothesize that low permeable **1**, which is assumed to be elongated due to the lack of medium- and long-range NOEs, populates a more polar ensemble than the ones of **2**, **6**, and **7**, rationalizing the low permeability of **1**. An alternative, or complementary, explanation for the low permeability of **1** is provided by studies of series of cyclic peptides, which found that permeability drops when the lipophilicity of the peptides in the series passes a threshold.^47,48^ It cannot be ruled out that somewhat higher lipophilicity of **1**, as compared to **2** and **6** (0.8–0.9 log units, Table 2), moves **1** over such a threshold.

### Intramolecular Interactions in Solution Ensembles

**Figure 5:**
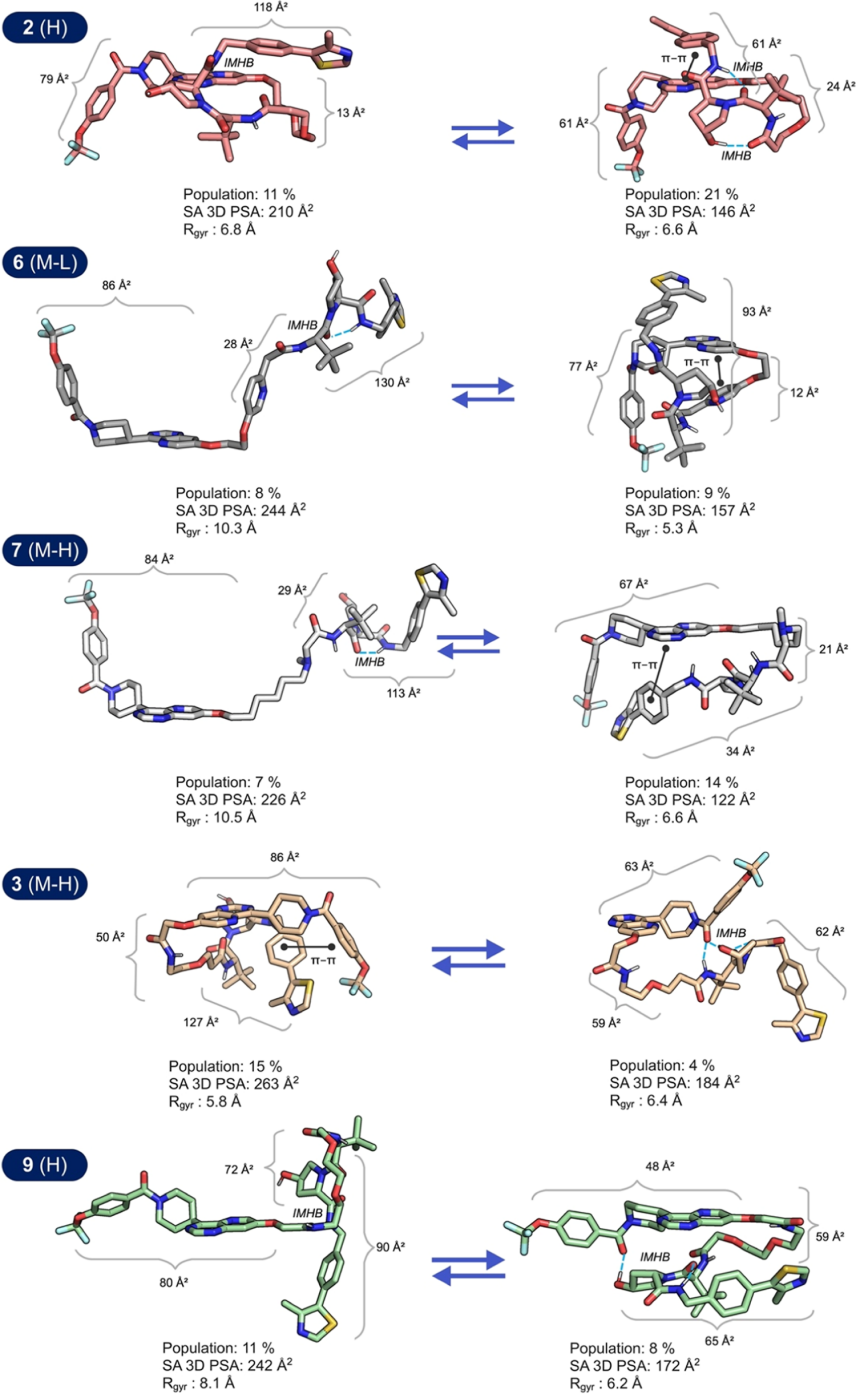
Comparisons of the conformations in the ensembles of PROTACs **2**, **6**, **7**, **3**, and **9** that have the highest and lowest surface accessible 3D polar surface area (SA 3D PSA). For each conformation the SA 3D PSA of the ERK5 and VHL ligands, and of the linker, is given adjacent to the corresponding bracket. Intramolecular hydrogen bonds (IMHBs) are indicated by blue dotted lines, and π–π interactions by black lines having a dot at each end. Conformations that have low populations (2 or 3%) have been omitted from the comparisons. The permeability class of each PROTAC is given in brackets after its number.

Dynamic IMHBs that are formed in an apolar environment, but broken in a polar one, can have a large impact on the polarity and size of a compound and thereby on key properties such as solubility, cell permeability and target binding potency.^49,50^ Dynamic IMHBs are therefore essential for compounds that behave as molecular chameleons, but dynamic shielding and exposure of polar groups by adjacent aryl and alkyl groups is also known to be important for chameleonicity.^21,45^ Perhaps surprisingly, the chloroform ensembles of PROTACs **2**, **3**, **6**, **7**, and **9** do not show a strong correlation between the number of IMHBs and the SA 3D PSA of the conformations (cf. color coding in Figure 4A). For example, the least polar conformations of PROTACs **6** and **7** do not display any IMHB, whereas the most polar conformations of **6** and **7** each contain one IMHB (Figures 4A and 5). Another example is provided by the ensemble of **3** which contains four conformations having a similar SA 3D PSA (184–192 Å^2^), while the number of IMHBs varies from 0 to 3 (Figure 4A). Similarly, strong correlations between other types of intramolecular interactions, such as π–π interactions, and the SA 3D PSA was not found for this series of PROTACs. IMHB-formation thus appears to act in combination with other intramolecular interactions for PROTACs that behave as molecular chameleons when crossing cell membranes.

We reasoned that inspection of the conformations in the ensembles of PROTACs **2**, **3**, **6**, **7**, and **9** should provide additional insight into how different intramolecular interactions may contribute to the reduction of polarity and size. This might, in turn, provide knowledge that can be used for design of PROTACs with increased cell permeability. Since the five PROTACs show large variations in SA 3D PSA between conformations (>60 Å^2^, Figure 4A) we analyzed the conformations having the lowest and highest SA 3D PSA for each PROTAC (Figure 5).

Comparison of the contributions of the SA 3D PSA of the three moieties of the PROTACs to the total SA 3D PSA in the low and high polarity conformations provided some high-level insights (Figure 5). For **2**, **6**, **7**, **3**, which all have an eight-atom linker, shielding of the polarity of the VHL moiety provides the major part of the reduction in SA 3D PSA between the high and low polarity conformations (37–79 Å^2^). The ERK5 and linker parts provided smaller contributions, i.e., 9–23 Å^2^ for the ERK5 ligand and 8–16 Å^2^ for the linker. PROTAC **9**, which has a longer 10-atom linker, differed as the ERK ligand contributed somewhat more than the VHL ligand to the PSA reduction (32 and 25 Å^2^, respectively) while the contribution from the linker was still low (13 Å^2^). Thus, design of VHL PROTACs for increased permeability should focus on the introduction of dynamic intramolecular interactions that shield the polarity of the VHL ligand.

**Figure 6:**
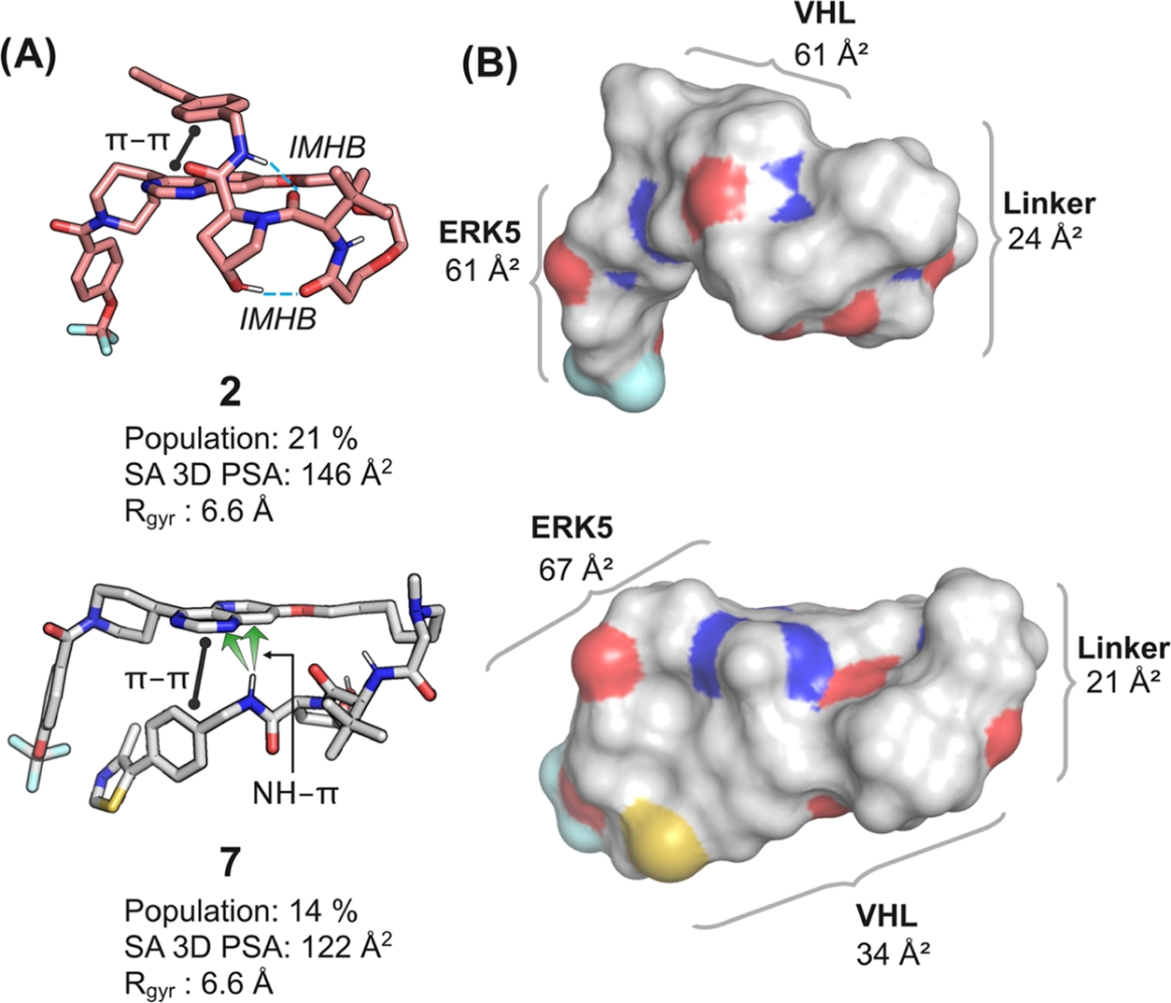
(A) Intramolecular interactions in the conformations of PROTACs **2** and **7** that have the lowest surface accessible 3D polar surface area (SA 3D PSA). Intramolecular hydrogen bonds (IMHBs) are indicated by blue dotted lines, π–π interactions by black lines having a dot at each end, and the NH-π interaction in the conformation of **7** by green arrows. (B) Surface representation of the two low SA 3D PSA conformations in the same orientation as in panel A. Surface exposed oxygen atoms are red, nitrogen atoms and nitrogen atoms carrying hydrogen atoms are blue, while the surface exposed sulfur atom in each PROTAC is yellow.

The contribution of the VHL ligand to the overall SA 3D PSA in the low PSA conformation was particularly reduced for PROTACs **2** and **7**, that have high and medium–high permeabilities, respectively. Inspection of the low-PSA conformations of these two PROTACs revealed how IMHBs, π–π interactions, NH–π interactions, and shielding of polar groups by alkyl groups all may contribute to the reduction of polarity for PROTACs (Figure 6). The low-PSA conformation of **2** contains two IMHBs that effectively remove two of the three HBDs from interactions with the surrounding environment (Figure 6A, top). The remaining HBD, i.e., the NH of the *tert*-butyl glycine moiety of the VHL ligand, is almost completely shielded from solvent by the adjacent *tert*-butyl group (Figure 6B, top), as also reported earlier in the conformational analysis of **9**.^20^ In addition, the conformation is stabilized by the formation of a π–π interaction between the heterocyclic part of the ERK5-ligand and the phenyl group at the distal end of the VHL ligand. IMHBs were not present in the low-PSA conformation of **7** (Figure 6A, bottom). Instead, the NH proton in the amine that is acylated by the hydroxyproline moiety forms an NH–π interaction with the heterocycle of the ERK5 ligand, while the NH of the *tert*-butyl glycine moiety is completely shielded from solvent by the adjacent *tert*-butyl group and the *N*-methylated amine of the linker (Figure 6B, bottom). The remaining HBD, i.e., the hydroxyl group of the hydroxyproline moiety is solvent exposed on the other face of the molecule. The conformation is also stabilized by a π–π interaction between the phenyl group of the VHL moiety and the heterocycle of the ERK5 ligand.

In spite of that PROTACs **2** and **7** both contain highly flexible linkers (as revealed by their NRotB counts, Table 2) they adopt turns that make them tightly folded in their low-PSA conformation where the SA 3D PSA of the VHL moiety is particularly reduced (Figure 6). For PROTACs, such as **2**, formation of turns have been proposed to be favored by the gauche effect of PEG-type linker.^51^ For PROTAC **7** the NH proton of the *tert*-butyl glycine residue is oriented toward the lone pair of the *N*-methylated amine in the linker and a minor conformational adjustment would result in the formation of a 5-membered pseudoring stabilized by an IMHB. We speculate that such a conformation could contribute to the nucleation of folding for **7** as a step toward low-PSA conformations. In summary, inspection of the low-PSA conformations of PROTACs **2** and **7** illustrate how different types of intramolecular interactions contribute to the folding and reduction of surface accessible 3D PSA of PROTACs. In addition, the conformational ensembles of the five PROTACs investigated herein highlight that design of VHL PROTACs for increased permeability should focus on the introduction of dynamic intramolecular interactions that shield the polarity of the VHL ligand. When the POI ligand contains aromatic moieties, design could involve stabilization of conformations that engage the HBDs of the VHL ligand in IMHBs, while also promoting the formation of π–π or NH–π interactions between the two ligands.

## Summary and Conclusions

We have designed and synthesized eight PROTACs which have identical ligands for VHL and ERK5, as well as linkers that are eight atoms long, to understand how differences in the flexibility, functionality and polarity of the linker influences the PROTAC′s membrane permeability. A previously reported^20^ VHL PROTAC that has a 10-atom long linker was also included in the study. Interestingly, small variations of the structure of the linker had a profound impact both on the passive cell permeability and on the permeability in the PAMPA assay for this set of VHL PROTACs. For example, replacement of two of the eight methylene groups in a linear aliphatic linker by oxygen atoms (cf. **1** and **2**, Table 2) led to close to 3 orders of magnitude improvement in PAMPA permeability and to a 22-fold increase in cell permeability. Similarly, replacement of one of the eight methylene groups by an *N*-methylated amine (cf. **1** and **7**, Table 2) resulted in a 40-fold improvement in PAMPA permeability and a 5-fold increase in cell permeability.

3D descriptors calculated for the solution ensembles determined by NMR spectroscopy in chloroform provided a rationalization of the observed differences in membrane permeability between four of the five PROTACs. PROTACs that were more permeable had ensembles characterized by an overall lower polarity, as determined by the solvent accessible 3D polar surface area (SA 3D PSA), and a smaller size, i.e., radius of gyration (*R*_gyr_). Similar trends were recently found for three CRBN PROTACs, which also differed only in the structure of the linker, and which were studied by NMR spectroscopy in combination with MD simulations.^30^ The permeability of PROTAC **7**, the linker of which contains a tertiary amine that is partially charged (<25%) at physiological pH, could also be rationalized in comparison to the four other, uncharged PROTACs, but with a larger degree of uncertainty. The ensembles of PROTACs **2** and **7**, which have high and medium–high permeability, respectively, revealed that their low-polarity conformations can hide large polar surface areas, in particular in the VHL ligand but also in the ERK5 ligand. Design of linkers, tailored to the structures of the two ligands, that facilitates folding of such low-polarity conformations appears as an attractive, albeit nontrivial approach toward cell permeable and orally bioavailable VHL PROTACs. This, and other approaches for dynamic, environment dependent reduction of polarity may be considerably more attractive for VHL PROTACs, which reside in a chemical space at or beyond the proposed outer guidelines for oral absorption,^4,15^ than for the somewhat smaller and less polar CRBN PROTACs.

Recent studies of CRBN PROTACs have suggested a dominant role of intramolecular hydrogen bonding for their physicochemical profile and cell permeability. A strong correlation was found between intramolecular hydrogen bonding, as predicted by conformational sampling, and molecular chameleonicity, as quantified by the chromatographic descriptor Chamelogk, for a set of six CRBN PROTACs which targeted the bromodomain and extraterminal domain (BET) proteins.^52^ In another study, 1D NMR spectroscopy was used to investigate intramolecular hydrogen bonding for 11 PROTACs, all but one of which were based on CRBN.^53^ An upper limit of two solvent exposed hydrogen bond donors was found for the PROTACs that had >10% oral bioavailability in the mouse, while a larger number of exposed hydrogen bond donors resulted in a very low oral bioavailability. In addition, structure–property relationship studies of 1806 PROTACs at Arvinas, most of which were likely based on CRBN, also found ≤2 exposed hydrogen bond donors as a cutoff for PROTACs to have a satisfactory oral absorption.^15^ Herein, we found that intramolecular hydrogen bonding alone does not account for the folding and polar surface area (PSA) reduction displayed by members of the series of VHL PROTACs studied. Instead, a complex interplay between intramolecular hydrogen bonds, NH–π and π–π interactions stabilize low-PSA conformations, while steric shielding of HBDs by adjacent alkyl groups also contributes to reduction of PSA and to higher cell permeability. Further studies are required to clarify how structurally different PROTACs rely on different types of intramolecular interactions for reduction of PSA when crossing cell membranes.

Once the POI and the E3-ligase ligands have been chosen, the linker is the moiety that allows the largest structural variation in optimization of a PROTAC toward an oral drug. The length and chemical composition of the linker is not only crucial for ternary complex formation with the POI and the E3-ligase, and thereby for target degradation, but it also influences the physicochemical and ADME properties of PROTACs.^54^ Since VHL ligands are larger, more polar and flexible than those for CRBN, putting VHL PROTACs close to the outer “limits” for oral absorption,^4,15^ the choice and optimization of the linker is crucial for VHL PROTACs intended for oral administration. Herein we have shown that systematic design of a set of linkers can result in large differences in the membrane permeability of VHL PROTACs, i.e., in one of the three properties that is crucial for oral bioavailability (the others being solubility and metabolism). Our work also suggests that rational design of oral VHL PROTACs could focus on linkers that facilitate the dynamic hiding of large polar surface areas in the VHL ligand in an apolar but not in a polar environment. While such a 3D-design approach is far from established, conformational sampling and/or MD simulations have shown promising results in the environment-dependent sampling of biologically relevant conformational space for individual PROTACs,^55^ or for small sets of structurally related PROTACs.^30,52^

## Experimental Section

### General Synthetic and Analytical Methods

All commercial reagents and catalysts were used as provided by the commercial supplier without purification. Solvents for synthesis, extraction, and chromatography were of reagent grade and used as received. Moisture-sensitive reactions were carried out under an atmosphere of argon, and anhydrous solvents were used as provided by the commercial supplier. Reaction progress was monitored by HPLC, LC/MS or thin layer chromatography. Crude products were immediately purified using preparative reversed-phase HPLC methodology with UV detection or flash chromatography on silica gel. The fractions obtained were concentrated in vacuo to remove organic volatiles.

^1^H NMR and ^13^C NMR spectra used to determine the identity and purity of PROTACs **1**–**8**, and the intermediates in their synthesis, were recorded in the solvents indicated below at RT with Bruker Avance spectrometers operating at 400 or 500 MHz for ^1^H NMR and at 126 MHz for ^13^C NMR spectroscopy. Chemical shifts for most compounds are reported in ppm relative to tetramethylsilane (TMS) as an internal standard, but for some compounds the residual peak from the deuterated solvent was used. The descriptions of the coupling patterns of ^1^H NMR signals are based on the optical appearance of the signals and do not necessarily reflect the physically correct interpretation. In general, the chemical shift information refers to the center of the signal. In the case of multiplets, intervals are given. Spin multiplicities are reported as s = singlet, br s = broad singlet, d = doublet, dd = doublet of doublets, t = triplet, q = quartet, m = multiplet.

The following mass spectrometry methods were used. LC/MS-Method 1: System MS: Thermo Scientific FT-MS; System UHPLC+: Thermo Scientific UltiMate 3000; Column: Waters, HSST3, 2.1 mm × 75 mm, C18 1.8 μm; Eluent A: 1 l Water + 0.01% Formic acid; Eluent B: 1 l Acetonitrile + 0.01% Formic acid; Gradient: 0.0 min 10% B → 2.5 min 95% B → 3.5 min 95% B; Oven: 50 °C; Flow: 0.90 mL/min; UV-Detection: 210 nm/Optimum Integration Path 210–300 nm. LC/MS-Method 2: System MS: Waters TOF instrument; System UPLC: Waters Acquity I-CLASS; Column: Waters, HSST3, 2.1 mm × 50 mm, C18 1.8 μm; Eluent A: 1 l Water +0.01% Formic acid; Eluent B: 1 l Acetonitrile +0.01% Formic acid; Gradient: 0.0 min 2% B → 0.5 min 2% B → 7.5 min 95% B → 10.0 min 95% B; Oven: 50 °C; Flow: 1.00 mL/min; UV-Detection: 210 nm. LC/MS-Method 3: System MS: Thermo Scientific FT-MS; System UHPLC+: Thermo Scientific Vanquish; Column: Waters, HSST3, 2.1 mm × 75 mm, C18 1.8 μm; Eluent A: 1 l Water +0.01% Formic acid; Eluent B: 1 l Acetonitrile +0.01% Formic acid; Gradient: 0.0 min 10% B → 2.5 min 95% B → 3.5 min 95% B; Oven: 50 °C; Flow: 0.90 mL/min; UV-Detection: 210 nm. LC/MS-Method 4: Instrument: Shimadzu LCMS-2020 SingleQuad; Column: Chromolith@Flash RP-18E 25–2 MM; Eluent A: Water +0.0375 vol % Trifluoroacetic acid, Eluent B: Acetonitrile +0.01875 vol % Trifluoroacetic acid; gradient: 0–0.8 min, 5–95% B, 0.8–1.2 min 95% B; Flow 1.5 mL/min; Temperature: 50 °C; PDA: 220 nm and 254 nm. Single Mass Analysis (HR-MS): Instrument: Waters time of flight system (ToF), electrospray ionization (ESI).

The purity of the synthetic intermediates was determined by reversed-phase LC/MS using one of methods 1–4, as reported below. PROTACs **1**–**8** are >95% pure as determined by reversed-phase LC/MS (cf. Supporting Information).

### Preparation of PROTAC **1** and Respective Starting Materials

#### Methyl 9-Iodononanoate

To a mixture of triphenylphosphine (2.5 g, 9.56 mmol), 1H-imidazole (814 mg, 11.95 mmol) and iodine (2.4 g, 9.56 mmol) in 10 mL THF was added methyl 9-hydroxynonanoate (1.5 g, 7.97 mmol). The mixture was stirred at room temperature for 1.5 h. The mixture was filtered. The filter cake was washed with ethyl acetate. The filtrate was concentrated in vacuo and the residue was purified by silica gel chromatography eluting with petroleum ether/ethyl acetate: 3:1 → 1:5 mesh: 200–300. 1.5 g (95% purity, 60% yield) of the title compound was obtained.

^1^H NMR (400 MHz, CDCl_3_) δ [ppm] = 3.67 (s, 3H), 3.18 (t, 2H), 2.31 (t, 2H), 1.83–1.80 (m, 2H), 1.64–1.60 (m, 2H), 1.38–1.31 (m, 8H).

#### Methyl9-[(4-{1-[4-(trifluoromethoxy)benzoyl]piperidin-4-yl}pyrido[3,2-*d*]pyrimidin-7-yl)oxy]nonanoate

To a mixture of methyl 9-iodononanoate (270 mg, 95% purity, 0.86 mmol) and potassium carbonate (198 mg, 1.43 mmol) in 4 mL DMF was added [4-(7-hydroxypyrido[3,2-*d*] pyrimidin-4-yl) piperidin-1-yl][4-(trifluoromethoxy)phenyl]methanone^20^ (300 mg, 0.72 mmol) and the mixture was stirred at 25 °C for 2 h. The mixture was poured into water and exacted with ethyl acetate. The combined organic layers were dried over anhydrous sodium sulfate and concentrated in vacuo. The residue was purified by silica gel column chromatography eluting with petroleum ether/ethyl acetate: 10:1 → 1:1, mesh: 200–300. 452 mg (93% purity, 83% yield) of the title compound was obtained.

LC-MS (Method 4): *R*_t_ = 0.827 min; MS (ESIpos): *m*/*z* = 589 [M + H]^+^.

^1^H NMR (400 MHz, DMSO-*d*_6_) δ [ppm] = 9.19 (s, 1H), 8.82 (s, 1H), 7.73 (d, 1H), 7.58 (d, 2H), 7.45 (d, 2H), 4.25 (t, 2H), 4.0 (m, 1H), 3.57 (s, 7H), 2.31–2.27 (m, 2H) 1.99–1.81 (m, 8H), 1.34–1.23 (m, 8H).

#### 9-[((4-{1-[4-Trifluoromethoxy) benzoyl]piperidin-4-yl}pyrido[3,2-*d*]pyrimidin-7-yl)oxy]nonanoic Acid

Methyl 9-[(4-{1-[4-(trifluoromethoxy)benzoyl]piperidin-4-yl}pyrido[3,2-*d*]pyrimidin-7-yl)oxy]nonanoate (400 mg, 93% purity, 0.63 mmol) was added to 4 mL of a mixture of water and methanol (v/v = 1:3), lithium hydroxide monohydrate (79 mg, 1.89 mmol) was added, and the mixture was stirred at 25 °C for 2 h. The mixture was poured into aqueous hydrochloric acid (1 mol/L) and extracted with ethyl acetate. The combined organic layers were dried over anhydrous sodium sulfate and concentrated in vacuo. 310 mg (94% purity, 81% yield) of the title compound was obtained.

LC-MS (Method 4): *R*_t_ = 1.035 min; MS (ESIpos): *m*/*z* = 575 [M + H]^+^.

#### 3-Methyl-*N*-{9-[(4-{1-[4-(trifluoromethoxy)benzoyl]piperidin-4-yl}pyrido[3,2-*d*]pyrimidin-7-yl)oxy]nonanoyl}-l-valyl-(4*R*)-4-hydroxy-*N*-[4-(4-methyl-1,3-thiazol-5-yl)benzyl]-l-prolinamide (**1**)

A mixture of 9-[(4-{1-[4-(trifluoromethoxy) benzoyl] piperidin-4-yl}pyrido[3,2-*d*]pyrimidin-7-yl)oxy]nonanoic acid (100 mg, 94% purity, 0,16 mmol), propane phosphonic anhydride (0.25 mL, 50% purity, 0.25 mmol) and *N*,*N*-diisopropylethylamine (0.06 mL, 0.33 mmol) in 1 mL DMF was stirred at 40 °C. After 30 min 3-methyl-l-valyl-(4*R*)-4-hydroxy-*N*-[4-(4-methyl-1,3-thiazol-5-yl) benzyl]-l-prolineamide (85 mg, 0.2 mmol) was added to the mixture and the mixture was stirred at 40 °C overnight. The reaction mixture was poured into ice cold ammonium chloride solution and extracted with ethyl acetate. The combined organic layers were washed with brine, dried over anhydrous sodium sulfate, and concentrated in vacuo. The residue was purified by preparative HPLC (Instrument: Gilson-281; Column: Phenomenex Luna C 18 75 mm × 30 mm × 3 μm; eluent A: 10 mM NH_4_HCO_3_ in water, eluent B: acetonitrile; gradient: 0 → 10 min 52 → 82% B; flow: 25 mL/min; temperature: room temperature; Detector: UV 220/254 nm). 23 mg (100% purity, 14% yield) of the title compound was obtained.

LC-MS (Method 4): *R*_t_ = 1.048 min; MS (ESIpos): *m*/*z* = 987 [M – H]^+^.

HRMS (ESI) *m*/*z*: calcd. for C_51_H_62_N_8_O_9_F_3_S [M + H]^+^, 987.4414; found, 987.4432.

NMR: See Supporting Information.

### Preparation of PROTAC **2** and Respective Starting Materials

#### *tert*-Butyl 3-[2-(2-hydroxyethoxy)ethoxy]propanoate

To 100 mL of anhydrous THF was added sodium (54 mg, 2.34 mmol) and 2,2′-oxidiethanol (41.4 g, 390 mmol) with stirring. After the sodium had completely dissolved, *tert*-butyl acrylate (10 g, 78 mmol) was added, and the solution was stirred at room temperature for 20 h. The mixture was poured into aqueous ammonium chloride solution and extracted with ethyl acetate. The combined organic layers were washed with brine, dried over anhydrous sodium sulfate, and concentrated in vacuo. The residue was purified by silica gel chromatography eluting with petroleum ether/ethyl acetate: 8:1 → 2:1 mesh: 100–200. 6.5 g (90% purity, 32% yield) of the title compound were obtained.

^1^H NMR (400 MHz, CDCl3-*d*_6_) δ [ppm] = 3.75–3.37 (m, 10H), 2.51 (t, 2H), 2.43 (s, 1H), 1.45 (s, 9H).

#### *tert*-Butyl 3-[2-(2-iodoethoxy)ethoxy]propanoate

To a mixture of triphenylphosphine (2.5 g, 9.56 mmol), 1H-imidazole (656 mg, 9.63 mmol) and iodine (3.1 g, 12.03 mmol) in 20 mL THF was added *tert*-butyl 3-[2-(2-hydroxyethoxy) ethoxy] propanoate (2.0 g, 8.02 mmol). The mixture was stirred at room temperature. After 1.5 h the mixture was filtered. The filter cake was washed with ethyl acetate. The filtrate was concentrated in vacuo and the residue was purified by silica gel chromatography eluting with petroleum ether/ethyl acetate: 20:1 → 4:1 mesh: 100–200. 2 g (90% purity, 65% yield) of the title compound were obtained.

^1^H NMR (400 MHz, CDCl3-*d*_6_) δ [ppm] = 3.76–3.72 (m, 4H), 3.70–3.62 (m, 4H), 3.25 (t, 2H), 2.50 (t, 2H), 1.44 (s, 9H).

#### *tert*-Butyl 3-(2-{2-[(4-{1-[4-(trifluoromethoxy)benzoyl]piperidin-4-yl}pyrido[3,2-*d*]pyrimidin-7-yl)oxy]ethoxy}ethoxy)propanoate

To a mixture of *tert*-butyl 3-[2-(2-iodoethoxy)ethoxy]propanoate (440 mg, 90% purity, 1.15 mmol) and potassium carbonate (265 mg, 1.91 mmol) in 10 mL DMF was added [4-(7-hydroxypyrido[3,2-*d*]pyrimidin-4-yl)piperidin-1-yl][4-(trifluoromethoxy)phenyl]methanone^20^ (401 mg, 0.96 mmol) and the mixture was stirred at 70 °C overnight. The mixture was poured into water and extracted with ethyl acetate. The combined organic layers were dried over anhydrous sodium sulfate and concentrated in vacuo. The residue was purified by silica gel column chromatography eluting with petroleum ether/ethyl acetate: 5:5 → 0:1, mesh: 100–200. 640 mg (94% purity, 82% yield) of the title compound were obtained.

LC-MS (Method 4): *R*_t_ = 0.936 min; MS (ESIpos): *m*/*z* = 635 [M + H]^+^.

#### 3-(2-{2-[(4-{1-[4-(Trifluoromethoxy)benzoyl]piperidin-4-yl}pyrido[3,2-*d*]pyrimidin-7-yl)oxy]ethoxy}ethoxy)propanoic Acid

A mixture of *tert*-butyl 3-(2-{2-[(4-{1-[4-(trifluoromethoxy)benzoyl] piperidin-4-yl}pyrido[3,2-*d*]pyrimidin-7-yl)oxy]ethoxy}ethoxy)propanoate (590 mg, 94% purity, 0.87 mmol) and trifluoroacetic acid (2.0 mL, 25.96 mmol) in 6 mL of dichloromethane was stirred at room temperature. After 3 h the mixture was poured into water and extracted with ethyl acetate. The combined organic layers were washed with brine, dried over anhydrous sodium sulfate, and filtered. The filtrate was concentrated in vacuo. 460 mg (93% purity, 85% yield) of the title compound were obtained.

LC-MS (Method 4): *R*_t_ = 0.886 min; MS (ESIpos): *m*/*z* = 579 [M + H]^+^.

#### 3-Methyl-*N*-[3-(2-{2-[(4-{1-[4-(trifluoromethoxy) benzoyl]piperidin-4-yl}pyrido[3,2-*d*]pyrimidin-7-yl)oxy]ethoxy}ethoxy)propanoyl]-l-valyl-(4R)-4-hydroxy-*N*-[4-(4-methyl-1,3-thiazol-5-yl)benzyl]-l-prolinamide (**2**)

A mixture of 3-(2-{2-[(4-{1-[4-(trifluoromethoxy)benzoyl]piperidin-4-yl}pyrido[3,2-*d*]pyrimidin-7-yl)oxy]ethoxy}ethoxy)propanoic acid (200 mg, 93% purity, 0.32 mmol), propane phosphonic anhydride (236 mg, 50% purity, 0.37 mmol) and *N*,*N*-diisopropylethylamine (0.09 mL, 0.49 mmol) in 3 mL DMF was stirred at 40 °C. After 30 min 3-methyl-l-valyl-(4*R*)-4-hydroxy-*N*-[4-(4-methyl-1,3-thiazol-5-yl) benzyl]-l-prolineamide (106 mg, 0.25 mmol) was added to the mixture and mixture was stirred at 40 °C overnight. The reaction mixture was poured into ice cold ammonium chloride solution and extracted with ethyl acetate. The combined organic layers were washed with brine, dried over anhydrous sodium sulfate, and concentrated in vacuo. The residue was purified by preparative HPLC (Instrument: Gilson-281; Column: Phenomenex Synergy C18 150 mm × 25 mm × 10 μm; eluent A: 0.225% formic acid in water, eluent B: acetonitrile; gradient: 0 → 10 min 42 → 62% B; flow 25 mL/min; temperature: room temperature; Detector: UV 220/254 nm). 131 mg (100% purity, 41% yield) of the title compound were obtained.

LC-MS (Method 2): *R*_t_ = 4.38 min; MS (ESIpos): *m*/*z* = 991 [M + H]^+^.

HRMS (ESI) *m*/*z*: calcd. for C_49_H_58_N_8_O_9_F_3_S [M + H]^+^, 991.4006; found, 991.4006.

NMR: See Supporting Information.

### Preparation of PROTAC **3** and Respective Starting Materials

#### *tert*-Butyl 3-(2-{[(benzyloxy)carbonyl] amino}ethoxy)propanoate

To a mixture of benzyl (2-hydroxyethyl) carbamate (10 g, 51.22 mmol) and *tert*-butyl acrylate (13.13 g, 102.45 mmol) in 25 mL of 1.4-dioxane was added a potassium hydroxide solution (9.5 g, 102.45 mmol, dissolved in water 60%) at 25 °C. The mixture was stirred at 25 °C overnight. The mixture was poured into ice cold aqueous ammonium chloride solution and filtered. The filter cake was suspended in ethyl acetate and filtered. The filtrate was washed with brine, dried over anhydrous sodium sulfate, and concentrated in vacuo. The residue was purified by silica gel chromatography eluting with petroleum ether/ethyl acetate: 20:1 → 3:1 mesh: 100–200. 8 g (90% purity, 43% yield) of the title compound were obtained.

^1^H NMR (400 MHz, CDCl_3_) δ [ppm] = 7.28–7.25 (m, 6H), 5.00 (s, 2H), 3.58 (t, 2H), 3.44–3.42 (m, 2H), 3.27 (m, 2H), 2.37 (t, 2H), 1.34 (s, 9H).

#### *tert*-Butyl 3-(2-aminoethoxy)propanoate

To a mixture of *tert*-butyl 3-(2-{[(benzyloxy)carbonyl] amino}ethoxy)propanoate (3 g, 90% purity, 8.35 mmol) in 20 mL THF was added palladium on carbon (200 mg, 0.83 mmol). The mixture was stirred under a hydrogen atmosphere (hydrogen balloon, 15 psi) for 2 h. The mixture was filtered, and the filter cake was washed with ethyl acetate. The filtrate was concentrated in vacuo. The residue was purified by silica gel chromatography eluting with petroleum ether/ethyl acetate: 3:1 → 1:4 mesh: 100–200. 1.5 g (90% purity, 85% yield) of the title compound were obtained.

^1^H NMR (400 MHz, CDCl_3_) δ [ppm] = 3.63 (t, 2H), 3.43 (t, 2H), 2.79 (t, 2H), 2.43 (t, 2H), 2.22 (t, 2H), 1.39 (s, 9H).

#### *tert*-Butyl [(4-{1-[4-(Trifluoromethoxy)benzoyl]piperidin-4-yl}pyrido[3,2-*d*]pyrimidin-7-yl)oxy]acetate

To a mixture of *tert*-butyl bromoacetate (213 mg, 1.09 mmol) and potassium carbonate (251 mg, 1.82 mmol) in 4 mL DMF was added [4-(7-hydroxypyrido[3,2-*d*]pyrimidin-4-yl)piperidin-1-yl][4-(trifluoromethoxy)phenyl]methanone^20^ (380 mg, 0.91 mmol) and the mixture was stirred at 70 °C overnight. The reaction mixture was filtered, and the filter cake was washed with ethyl acetate. The filtrate was poured into ice cold ammonium chloride solution. The resulting mixture was extracted with ethyl acetate. The combined organic layers were washed with brine, dried over anhydrous sodium sulfate, and concentrated in vacuo. The residue was purified by silica gel chromatography eluting with petroleum ether/ethyl acetate: 6:1 → 3:1 mesh: 100–200. 450 mg (95% purity, 88% yield) of the title compound were obtained.

LC-MS (Method 4): *R*_t_ = 0.995 min; MS (ESIpos): *m*/*z* = 533 [M + H]^+^.

#### [((4-{1-[4-Trifluoromethoxy)benzoyl]piperidin-4-yl}pyrido[3,2-*d*]pyrimidin-7-yl)oxy]acetic Acid

A mixture of *tert*-butyl [(4-{1-[4-(trifluoromethoxy)benzoyl]piperidin-4-yl}pyrido[3,2-*d*]pyrimidin-7-yl)oxy]acetate (400 mg, 95% purity, 0.71 mmol) and trifluoroacetic acid (2.0 mL, 25.96 mmol) in 6 mL of dichloromethane was stirred at room temperature. After 3 h the mixture was poured into water and extracted with ethyl acetate. The combined organic layers were washed with brine, dried over anhydrous sodium sulfate, and filtered. The filtrate was concentrated in vacuo. 300 mg (95% purity, 84% yield) of the title compound were obtained.

LC-MS (Method 4): *R*_t_ = 0.861 min; MS (ESIpos): *m*/*z* = 477 [M + H]^+^.

#### *tert*-Butyl 3-(2-{2-[(4-{1-[4-(trifluoromethoxy)benzoyl]piperidin-4-yl}pyrido[3,2-*d*]pyrimidin-7-yl)oxy]acetamido}ethoxy)propanoate

A mixture of [(4-{1-[4-(trifluoromethoxy)benzoyl]piperidin-4-yl}pyrido[3,2-*d*]pyrimidin-7-yl)oxy]acetic acid (250 mg, 95% purity, 0.5 mmol), propane phosphonic anhydride (476 mg, 50% purity, 0.75 mmol) and *N*,*N*-diisopropylethylamine (0.17 mL, 1 mmol) in 5 mL DMF was stirred at 25 °C. After 20 min *tert*-butyl 3-(2-aminoethoxy) propanoate (157 mg, 0.75 mmol) was added to the mixture which was then stirred at 25 °C overnight. The reaction mixture was poured into ice cold ammonium chloride solution and extracted with ethyl acetate. The combined organic layers were washed with brine, dried over anhydrous sodium sulfate, and concentrated in vacuo. The residue was purified by silica gel chromatography eluting with petroleum ether/ethyl acetate: 20:1 → 3:1 mesh: 100–200. 320 mg (93% purity, 92% yield) of the title compound were obtained.

LC-MS (Method 4): *R*_t_ = 0.884 min; MS (ESIpos): *m*/*z* = 648 [M + H]^+^.

#### 3-(2-{2-[(4-{1-[4-(Trifluoromethoxy)benzoyl]piperidin-4-yl}pyrido[3,2-*d*]pyrimidin-7-yl)oxy]acetamido}ethoxy)propanoic Acid

A mixture of *tert*-butyl 3-(2-{2-[(4-{1-[4-(trifluoromethoxy)benzoyl]piperidin-4-yl}pyrido[3,2-*d*]pyrimidin-7-yl)oxy]acetamido}ethoxy)propanoate (320 mg, 93% purity, 0.46 mmol) and trifluoroacetic acid (1.0 mL, 12.98 mmol) in 3 mL of dichloromethane was stirred at room temperature. After 3 h the mixture was concentrated in vacuo. The residue was triturated with MTBE. The precipitated solid was filtered off, and dried in vacuo. 220 mg (99% purity, 80% yield) of the title compound were obtained.

LC-MS (Method 4): *R*_t_ = 0.787 min; MS (ESIpos): *m*/*z* = 592 [M + H]^+^.

#### 3-Methyl-*N*-[3-(2-{2-[(4-{1-[4-(trifluoromethoxy)benzoyl]piperidin-4-yl}pyrido[3,2-*d*]pyrimidin-7-yl)oxy]acetamido}ethoxy)propanoyl]-l-valyl-(4*R*)-4-hydroxy-*N*-[4-(4-methyl-1,3-thiazol-5-yl)benzyl]-l-prolinamide (**3**)

A mixture of 3-(2-{2-[(4-{1-[4-(trifluoromethoxy)benzoyl]piperidin-4-yl}pyrido[3,2-*d*]pyrimidin-7-yl)oxy]acetamido}ethoxy)propanoic acid (170 mg, 99% purity, 0.28 mmol), propane phosphonic anhydride (272 mg, 50% purity, 0.43 mmol) and *N*,*N*-diisopropylethylamine (0.1 mL, 0.57 mmol) in 2 mL DMF was stirred at 40 °C. After 30 min 3-methyl-l-valyl-(4S)-4-hydroxy-*N*-[4-(4-methyl-1,3-thiazol-5-yl)benzyl]-l-prolinamide (147 mg, 0.34 mmol) was added to the mixture and the mixture was stirred at 40 °C overnight. The reaction mixture was poured into ice cold ammonium chloride solution and filtered. The filter cake was suspended in ethyl acetate and filtered. The filtrate was washed with brine, dried over anhydrous sodium sulfate, and concentrated in vacuo. The residue was purified by preparative HPLC (Instrument: Gilson-281; Column: Phenomenex Luna C18 75 mm × 30 mm × 3 μm; eluent A: 0.225% formic acid in water, eluent B: acetonitrile; gradient: 0 → 10 min 42 → 62% B; flow 25 mL/min; temperature: room temperature; Detector: UV 220/254 nm) and lyophilized. 71 mg (98% purity, 24% yield) of the title compound were obtained.

LC-MS (Method 2): *R*_t_ = 4.11 min; MS (ESIpos): *m*/*z* = 1004 [M + H]^+^.

HRMS (ESI) *m*/*z*: calcd. for C_49_H_58_N_9_O_9_F_3_S [M + H]^+^, 1004.3952; found, 1004.3947.

NMR: See Supporting Information.

### Preparation of PROTAC **4** and Respective Starting Materials

#### *tert*-Butyl (prop-2-yn-1-yloxy)acetate

To a mixture of prop-2-yn-1-ol (5.23 g, 93.29 mmol) in 50 mL THF was added sodium hydride (3.36 g, 60%, 83.96 mmol) at 0 °C. The mixture was stirred at 0 °C for 10 min. Then *tert*-butyl bromoacetate (14.56 g, 74.63 mmol) was added to the resulting suspension and the mixture was stirred at room temperature overnight. The mixture was poured into saturated aqueous ammonium chloride solution and exacted with ethyl acetate. The combined organic layers were dried over anhydrous sodium sulfate and concentrated in vacuo. The residue was purified by silica gel column chromatography eluting with petroleum ether/ethyl acetate: 100:1 → 2:1, mesh: 300–400. 3.1 g (90% purity, 18% yield) of the title compound were obtained.

^1^H NMR (400 MHz, CDCl_3_) δ [ppm] = 4.27 (d, 2H), 4.05 (s, 2H), 2.45 (t, 1H), 1.45 (s, 9H).

#### *tert*-Butyl {[1-(2-Hydroxyethyl)-1*H*-1,2,3-triazol-4-yl]methoxy}acetate

Under a nitrogen atmosphere, copper(I) iodide (604 mg, 3.17 mmol) and triethylamine (4.42 mL, 31.73 mmol) were added to a solution of *tert*-butyl (prop-2-yn-1-yloxy)acetate (3.0 g, 90%, 15.86 mmol) and 2-azidoethanol (1.93 g, 22.21 mmol) in 30 mL DMF. The mixture was stirred at 20 °C for 16 h under nitrogen. The reaction mixture was diluted with water and extracted with ethyl acetate. The combined organic layers were dried over anhydrous sodium sulfate, filtered, and concentrated in vacuo. The residue was purified by silica gel column chromatography eluting with dichloromethane/methanol: 50:1 → 10:1, 100–200 mesh. 3.1 g (99% purity, 75% yield) of the title compound were obtained.

LC-MS (Method 4): *R*_t_ = 0.425 min; MS (ESIpos): *m*/*z* = 258 [M + H]^+^.

^1^H NMR (400 MHz, CDCl_3_) δ [ppm] = 7.77 (s, 1H), 4.73 (s, 2H), 4.49 (t, 2H), 4.07–4.04 (m, 4H), 1.47 (s, 9H).

#### *tert*-Butyl [(1-{2-[(4-{1-[4-(trifluoromethoxy)benzoyl]piperidin-4-yl}pyrido[3,2-*d*]pyrimidin-7-yl)oxy]ethyl}-1H-1,2,3-triazol-4-yl)methoxy]acetate

To a solution of [4-(7-hydroxypyrido[3,2-*d*]pyrimidin-4-yl)piperidin-1-yl][4-(trifluoromethoxy)phenyl]methanone^20^ (300 mg, 717 μmol), *tert*-butyl {[1-(2-hydroxyethyl)-1H-1,2,3-triazol-4-yl]methoxy}acetate (252 mg, 99%, 932 μmol) and triphenylphosphine (376 mg, 1.43 mmol) in 3 mL dry THF at 0 °C was added diisopropyl (*E*)-diazene-1,2-dicarboxylate (290 mg, 1.43 mmol) dropwise. After addition, the mixture was stirred at room temperature overnight. The reaction mixture was diluted with water and extracted with ethyl acetate. The combined organic layers were dried over anhydrous sodium sulfate, filtered, and concentrated in vacuo. The residue was purified by silica gel column chromatography eluting with petroleum ether/ethyl acetate: 6:1 → 1:2, 100–200 mesh. 450 mg (95% purity, 91% yield) of the title compound were obtained.

LC-MS (Method 4): *R*_t_ = 0.872 min; MS (ESIpos): *m*/*z* = 658 [M + H]^+^.

#### [(1-{2-[(4-{1-[4-(Trifluoromethoxy)benzoyl]piperidin-4-yl}pyrido[3,2-*d*]pyrimidin-7-yl)oxy]ethyl}-1H-1,2,3-triazol-4-yl)methoxy]acetic Acid

A mixture of *tert*-butyl [(1-{2-[(4-{1-[4-(trifluoromethoxy)benzoyl]piperidin-4-yl}pyrido[3,2-*d*]pyrimidin-7-yl)oxy]ethyl}-1H-1,2,3-triazol-4-yl)methoxy]acetate (450 mg, 95%, 650 μ mol) and trifluoroacetic acid (2.0 mL, 26 mmol) in 6 mL DCM was stirred at room temperature for 3 h. The reaction mixture was concentrated in vacuo. The residue was diluted with ethyl acetate and washed with sodium bicarbonate solution. The combined organic layers were dried over anhydrous sodium sulfate, filtered, and concentrated in vacuo. 290 mg (73% yield) of the title compound were obtained.

#### 3-Methyl-*N*-{[(1-{2-[(4-{1-[4-(trifluoromethoxy)benzoyl]piperidin-4-yl}pyrido[3,2-*d*]pyrimidin-7-yl)oxy]ethyl}-1*H*-1,2,3-triazol-4-yl)methoxy]acetyl}-l-valyl-(4*R*)-4-hydroxy-*N*-[4-(4-methyl-1,3-thiazol-5-yl)benzyl]-l-prolinamide (**4**)

A mixture of [(1-{2-[(4-{1-[4-(trifluoromethoxy)benzoyl]piperidin-4-yl}pyrido[3,2-*d*]pyrimidin-7-yl)oxy]ethyl}-1*H*-1,2,3-triazol-4-yl)methoxy]acetic acid (200 mg, 99% purity, 329 μmol), propane phosphonic anhydride (314 mg, 50% purity, 494 μmol) and *N*,*N*-diisopropylethylamine (0.12 mL, 658 μmol) in 2 mL DMF was stirred at 40 °C. After 30 min 3-methyl-l-valyl-(4*S*)-4-hydroxy-*N*-[4-(4-methyl-1,3-thiazol-5-yl)benzyl]-l-prolinamide (170 mg, 395 μmol) was added to the mixture and the mixture was stirred at 40 °C overnight. The reaction mixture was poured into ice cold ammonium chloride solution and filtered. The filter cake was suspended in ethyl acetate and filtered. The filtrate was washed with brine, dried over anhydrous sodium sulfate, filtered, and concentrated in vacuo. The residue was purified by preparative HPLC (Instrument: Gilson-281; Column: Phenomenex Synergi C18 150 mm × 25 mm × 10 μm; eluent A: 0.225% formic acid in water, eluent B: acetonitrile; gradient: 0 → 10 min 45 → 5 9% B; flow 25 mL/min; temperature: room temperature; Detector: UV 220/254 nm) and lyophilized. 124 mg (100% purity, 37% yield) of the title compound were obtained.

LC-MS (Method 2): *R*_t_ = 4.16 min; MS (ESIpos): *m*/*z* = 1014 [M + H]^+^.

HRMS (ESI) *m*/*z*: calcd. for C_49_H_55_N_11_O_8_F_3_S [M + H]^+^, 1014.3908; found, 1014.3907.

NMR: See Supporting Information.

### Preparation of PROTAC **5** and Respective Starting Materials

#### Methyl [4-(2-Bromoethoxy)phenyl]acetate

To a mixture of 1,2-dibromoethane (45.2 g, 240.7 mmol) and potassium carbonate (12.5 g, 90.3 mmol) in 50 mL acetonitrile was added methyl (4-hydroxyphenyl) acetate (5.0 g, 30.1 mmol) and the mixture was stirred at 80 °C overnight. The reaction mixture was poured into saturated aqueous ammonium chloride solution and extracted with ethyl acetate. The combined organic layers were concentrated in vacuo and the residue was purified by silica gel column chromatography eluting with petroleum ether/ethyl acetate: 10:1 → 1:3, 100–200 mesh. 2.5 g (95% purity, 29% yield) of the title compound were obtained.

^1^H NMR (400 MHz, DMSO-*d*_6_) δ [ppm] = 7.18 (d, 2H), 6.90 (d, 2H), 4.29 (t, 2H), 3.79 (t, 2H), 3.60–3.33 (m, 5H).

#### Methyl(4-{2-[(4-{1-[4-(trifluoromethoxy)benzoyl]piperidin-4-yl}pyrido[3,2-*d*]pyrimidin-7-yl)oxy]ethoxy}phenyl)acetate

A mixture of [4-(7-hydroxypyrido[3,2-*d*]pyrimidin-4-yl)piperidin-1-yl][4-(trifluoromethoxy)phenyl]methanone^20^ (243 mg, 578 μmol), methyl [4-(2-bromoethoxy)phenyl]acetate (200 mg, 95%, 696 μmol) and potassium carbonate (160 mg, 1.2 mmol) in 3 mL DMF was stirred at 80 °C overnight. The reaction mixture was poured into ammonium chloride solution and extracted with ethyl acetate. The combined organic layers were washed with brine, dried over anhydrous sodium sulfate, filtered, and concentrated in vacuo. The residue was purified by silica gel column chromatography eluting with petroleum ether/ethyl acetate: 10:1 → 1:1, 100–200 mesh. 340 mg (95% purity, 91% yield) of the title compound were obtained.

LC-MS (Method 4): *R*_t_ = 1.022 min; MS (ESIpos): *m*/*z* = 611 [M + H]^+^.

^1^H NMR (400 MHz, CDCl_3_) δ [ppm] = 9.24 (s, 1H), 8.8 (d, 1H), 7.62–7.5 (m, 3H), 7.31–7.24 (m, 4H), 6.95 (d, 2H), 4.92 (m, 2H), 4.55–4.50 (m, 2H), 3.9–3.85 (m, 2H), 3.71 (s, 3H), 3.60 (s, 2H), 3.45–3.05 (m, 2H), 2.10–2.23 (m, 4H), 1.98–1.85 (m, 1H).

#### (4-{2-[(4-{1-[4-(Trifluoromethoxy)benzoyl]piperidin-4-yl}pyrido[3,2-*d*]pyrimidin-7-yl)oxy]ethoxy}phenyl)acetic Acid

A mixture of methyl(4-{2-[(4-{1-[4-(trifluoromethoxy)benzoyl]piperidin-4-yl}pyrido[3,2-*d*]pyrimidin-7-yl)oxy]ethoxy}phenyl)acetate (300 mg, 95%, 467 μmol), lithium hydroxide monohydrate (78 mg, 1.87 mmol), 2 mL water and 3 mL methanol was stirred at 25 °C for 2 h. The reaction mixture was adjusted to pH 3–4 with hydrochloric acid solution (1 mol/L in water) and extracted with ethyl acetate. The combined organic layers were dried over anhydrous sodium sulfate, filtered, and concentrated in vacuo. The crude product was purified by silica gel column chromatography eluting with petroleum ether/ethyl acetate: 1:5 → 0:1, 200–300 mesh. 290 mg (93% purity, 97% yield) of the title compound were obtained.

LC-MS (Method 4): *R*_t_ = 0.966 min; MS (ESIpos): *m*/*z* = 597 [M + H]^+^.

^1^H NMR (400 MHz, DMSO-*d*_6_) δ [ppm] = 12.17 (s, 1H), 9.22 (s, 1H), 8.89 (d, 1H), 7.86 (t, 1H), 7.59–7.57 (d, 2H), 7.45 (d, 2H), 7.18 (d, 2H), 6.94 (d, 2H), 4.63–4.62 (m, 3H), 4.41–4.39 (m, 3H), 3.71 (m, 1H), 3.70 (s, 2H), 3.05 (m, 1H), 1.99 (s, 1H), 1.91 (m, 4H).

#### 3-Methyl-*N*-[(4-{2-[(4-{1-[4-(trifluoromethoxy)benzoyl]piperidin-4-yl}pyrido[3,2-*d*]pyrimidin-7-yl)oxy]ethoxy}phenyl)acetyl]-l-valyl-(4*R*)-4-hydroxy-*N*-[4-(4-methyl-1,3-thiazol-5-yl)benzyl]-l-prolinamide (**5**)

A mixture of (4-{2-[(4-{1-[4-(trifluoromethoxy)benzoyl]piperidin-4-yl}pyrido[3,2-*d*]pyrimidin-7-yl)oxy]ethoxy}phenyl)acetic acid (200 mg, 93% purity, 312 μmol), propane phosphonic anhydride (248 mg, 50% purity, 390 μmol) and N,N-diisopropylethylamine (0.09 mL, 520 μmol) in 2 mL DMF was stirred at 40 °C. After 30 min 3-methyl-l-valyl-(4*S*)-4-hydroxy-*N*-[4-(4-methyl-1,3-thiazol-5-yl)benzyl]-l-prolinamide (111 mg, 260 μmol) was added to the mixture and the mixture was stirred at 40 °C overnight. The reaction mixture was poured into ice cold ammonium chloride solution and extracted with ethyl acetate. The combined organic layers were washed with brine, dried over anhydrous sodium sulfate, filtered, and concentrated in vacuo. The residue was purified by preparative HPLC (Instrument: Gilson-281; Column: Phenomenex Luna C18 75 mm × 30 mm × 3 μm; eluent A: 10 mM NH_4_HCO_3_, eluent B: acetonitrile; gradient: 0 → 10 min 43 → 73% B; flow 25 mL/min; temperature: room temperature; Detector: UV 220/254 nm) and lyophilized. 60 mg (100% purity, 19% yield) of the title compound were obtained.

LC-MS (Method 2): *R*_t_ = 4.71 min; MS (ESIpos): *m*/*z* = 1009 [M + H]^+^.

HRMS (ESI) *m*/*z*: calcd. for C_52_H_56_N_8_O_8_F_3_S [M + H]^+^, 1009.3894; found, 1009.3904.

NMR: See Supporting Information.

### Preparation of PROTAC **6** and Respective Starting Materials

#### 5-{[*tert*-Butyl(dimethyl)silyl]oxy}-2-methylpyridine

Under a nitrogen atmosphere *tert*-butyl(chloro)dimethylsilane (12.43 g, 82.5 mmol) was added dropwise into a mixture of 6-methylpyridin-3-ol (10.0 g, 91.63 mmol) and 1*H*-imidazole (7.49 g, 110 mmol) in 40 mL DMF. The mixture was stirred at 25 °C for 2 h. The reaction mixture was poured into saturated aqueous ammonium chloride solution and extracted with ethyl acetate. The combined organic layers were concentrated in vacuo and the residue was purified by silica gel column chromatography eluting with petroleum ether/ethyl acetate: 100:1 → 20:1, 100–200 mesh. 15.4 g (90% purity, 68% yield) of the title compound were obtained.

^1^H NMR (400 MHz, DMSO-*d*_6_) δ [ppm] = 8.04 (d, 1H), 7.19 (m, 2H), 2.38 (s, 3H), 0.94 (s, 9H), 0.18 (s, 6H).

#### Methyl (5-{[*tert*-Butyl(dimethyl)silyl]oxy}pyridin-2-yl)acetate

Under a nitrogen atmosphere butyllithium (25.14 mL, 62.85 mmol) was added at −70 °C to a solution of diisopropylamine (8.81 mL, 62.85 mmol) and the mixture was stirred at −70 °C. After 30 min 5-{[*tert*-butyl(dimethyl)silyl]oxy}-2-methylpyridine (13.0 g, 52.37 mmol) was added and the mixture was stirred at −70 °C for 10 min before dimethyl carbonate (5.66 g, 62.85 mmol) was added dropwise. The mixture was stirred another 1 h at −70 °C. Then, the reaction mixture was poured into saturated aqueous ammonium chloride solution and extracted with ethyl acetate. The combined organic layers were washed with brine, dried over anhydrous sodium sulfate, and filtered. The filtrate was concentrated in vacuo and the residue was purified by silica gel column chromatography eluting with petroleum ether/ethyl acetate: 10:1 → 1:1, 100–200 mesh. 12.5 g (95% purity, 81% yield) of the title compound were obtained.

^1^H NMR (400 MHz, DMSO-*d*_6_) δ [ppm] = 8.1 (t, 1H), 7.29–7.27 (m, 2H), 3.78 (s, 2H), 3.62 (s, 3H), 0.96 (s, 9H), 0.21 (s, 6H).

#### Methyl (5-Hydroxypyridin-2-yl)acetate

Tetra-*n*-butylammonium fluoride solution (40.0 mL, 40 mmol) was added dropwise to a solution of methyl (5-{[*tert*-butyl(dimethyl)silyl]oxy}pyridin-2-yl)acetate (12,0 g, 40.51 mmol) in 100 mL THF and the mixture was stirred at room temperature overnight. The reaction mixture was poured into saturated aqueous ammonium chloride solution and extracted with ethyl acetate. The combined organic layers were washed with brine, dried over anhydrous sodium sulfate, and filtered. The filtrate was concentrated in vacuo and the residue was purified by silica gel column chromatography eluting with petroleum ether/ethyl acetate: 10:1 → 1:3, 100–200 mesh. 5.5 g (95% purity, 77% yield) of the title compound were obtained.

^1^H NMR (400 MHz, DMSO-*d*_6_) δ [ppm] = 9.80 (s, 1H), 8.02 (d, 1H), 7.17–7.10 (m, 2H), 3.71 (s, 2H), 3.60 (s, 3H).

#### Methyl [5-(2-Bromoethoxy)pyridin-2-yl]acetate

To a mixture of 1,2-dibromoethane (8.54 g, 45.5 mmol) and potassium carbonate (2.36 g, 17.0 mmol) in 10 mL acetonitrile was added methyl (5-hydroxypyridin-2-yl)acetate (1.0 g, 95%, 5.7 mmol) and the resulting mixture was stirred at 80 °C overnight. The reaction mixture was poured into saturated aqueous ammonium chloride solution and extracted with ethyl acetate. The combined organic layers were concentrated in vacuo and the residue was purified by silica gel column chromatography eluting with petroleum ether/ethyl acetate: 10:1 → 1:3, 100–200 mesh. 1.1 g (95% purity, 67% yield) of the title compound were obtained.

^1^H NMR (400 MHz, DMSO-*d*_6_) δ [ppm] = 8.21 (d, 1H), 7.40 (d, 1H), 7.30 (d, 1H), 4.39 (t, 2H), 3.82 (t, 2H), 3.80 (s, 2H), 3.61 (s, 3H).

#### Methyl (5-{2-[(4-{1-[4-(Trifluoromethoxy)benzoyl]piperidin-4-yl}pyrido[3,2-*d*]pyrimidin-7-yl)oxy]ethoxy}pyridin-2-yl)acetate

A mixture of [4-(7-hydroxypyrido[3,2-*d*]pyrimidin-4-yl)piperidin-1-yl][4-(trifluoromethoxy)phenyl]methanone^20^ (400 mg, 956 μmol), methyl [5-(2-bromoethoxy)pyridin-2-yl]acetate (331 mg, 95%, 1.15 mmol) and potassium carbonate (264 mg, 1.9 mmol) in 8 mL DMF was stirred at 70 °C overnight. The reaction mixture was filtered, and the filter cake was washed with methanol. The filtrate was concentrated in vacuo and the residue was purified by reverse phase HPLC. 550 mg (98% purity, 92% yield) of the title compound were obtained.

LC-MS (Method 4): *R*_t_ = 0.625 min; MS (ESIpos): *m*/*z* = 612 [M + H]^+^.

#### (5-{2-[(4-{1-[4-(Trifluoromethoxy)benzoyl]piperidin-4-yl}pyrido[3,2-*d*]pyrimidin-7-yl)oxy]ethoxy}pyridin-2-yl)acetic Acid

To a solution of methyl (5-{2-[(4-{1-[4-(trifluoromethoxy)benzoyl]piperidin-4-yl}pyrido[3,2-*d*]pyrimidin-7-yl)oxy]ethoxy}pyridin-2-yl)acetate (500 mg, 98%, 800 μmol) in 4 mL methanol was added a solution of lithium hydroxide monohydrate (101 mg, 2.4 mmol) in 2 mL water. The mixture was stirred 25 °C for 2 h. Hydrochloric acid was added, and the mixture was extracted with ethyl acetate. The combined organic layers were washed with brine and dried over anhydrous sodium sulfate and filtered. The filtrate was concentrated in vacuo. 425 mg (94% purity, 83% yield) of the title compound were obtained.

LC-MS (Method 4): *R*_t_ = 0.584 min; MS (ESIpos): *m*/*z* = 598 [M + H]^+^.

#### 3-Methyl-*N*-[(5-{2-[(4-{1-[4-(trifluoromethoxy)benzoyl]piperidin-4-yl}pyrido[3,2-*d*]pyrimidin-7-yl)oxy]ethoxy}pyridin-2-yl)acetyl]-l-valyl-(4*R*)-4-hydroxy-*N*-[4-(4-methyl-1,3-thiazol-5-yl)benzyl]-l-prolinamide (**6**)

A mixture of (5-{2-[(4-{1-[4-(trifluoromethoxy)benzoyl]piperidin-4-yl}pyrido[3,2-*d*]pyrimidin-7-yl)oxy]ethoxy}pyridin-2-yl)acetic acid (200 mg, 94% purity, 313 μmol), propane phosphonic anhydride (399 mg, 50% purity, 626 μmol) and *N*,*N*-diisopropylethylamine (0.22 mL, 1.25 mmol) in 2 mL DMF was stirred at 40 °C. After 30 min 3-methyl-l-valyl-(4*S*)-4-hydroxy-*N*-[4-(4-methyl-1,3-thiazol-5-yl)benzyl]-l-prolinamide (161 mg, 376 μmol) was added to the mixture and the mixture was stirred at 40 °C overnight. The reaction mixture was poured into ice cold ammonium chloride solution and extracted with ethyl acetate. The combined organic layers were washed with brine, dried over anhydrous sodium sulfate, filtered, and concentrated in vacuo. The residue was purified by preparative HPLC (Instrument: Gilson-281; Column: Phenomenex Synergi C18 150 mm × 25 mm × 10 μm; eluent A: 0.225% formic acid in water, eluent B: acetonitrile; gradient: 0 → 10 min 45 → 59% B; flow 25 mL/min; temperature: room temperature; Detector: UV 220/254 nm) and lyophilized. 60 mg (100% purity, 19% yield) of the title compound were obtained.

LC-MS (Method 2): *R*_t_ = 4.34 min; MS (ESIpos): *m*/*z* = 1010 [M + H]^+^.

HRMS (ESI) *m*/*z*: calcd. for C_51_H_55_N_9_O_8_F_3_S [M + H]^+^, 1010.3846; found, 1010.3849.

NMR: See Supporting Information.

### Preparation of PROTAC **7** and Respective Starting Materials

#### *tert*-Butyl (6-Iodohexyl)methylcarbamate

To a mixture of triphenylphosphine (1.36 g, 5.19 mmol), 1H-imidazole (441 mg, 6.48 mmol) and iodine (1.32 g, 5.19 mmol) in 10 mL of THF was added *tert*-butyl (6-hydroxyhexyl)methylcarbamate (1.0 g, 4.32 mmol). The mixture was stirred at room temperature overnight. The solid was filtered off, washed with ethyl acetate and the filtrate was concentrated. The residue was purified by column chromatography (Machine: Biotage Isolera; column: Biotage Sfär HC 25 g; eluent: Cy/EE: 5% EE → 20% EE; flow: 80 mL/min). Product containing samples were united and the solvents were evaporated. 933 mg (100% purity, 63% yield) of the title compound were obtained.

LC-MS (Method 1): *R*_t_ = 2.44 min; MS (ESIpos): *m*/*z* = 286 [M + H – C_4_H_8_]^+^.

#### *tert*-Butyl Methyl{6-[(4-{1-[4-(trifluoromethoxy)benzoyl]piperidin-4-yl}pyrido[3,2-*d*]pyrimidin-7-yl)oxy]hexyl}carbamate

To a mixture of *tert*-butyl (6-iodohexyl)methylcarbamate (489 mg, 1.43 mmol) and potassium carbonate (330 mg, 2.39 mmol) in 5 mL DMF was added [4-(7-hydroxypyrido[3,2-*d*]pyrimidin-4-yl)piperidin-1-yl][4-(trifluoromethoxy)phenyl]methanone^20^ (500 mg, 1.19 mmol) and the mixture was stirred at room temperature overnight. The mixture was poured into water and extracted with ethyl acetate. The combined organic layers were washed with water and brine, dried over anhydrous sodium sulfate, and concentrated in vacuo. The residue was purified by column chromatography (Machine: Biotage Isolera; column: Biotage Sfär HC 25 g; eluent: Cy/EE: 12% EE → 100% EE; flow: 80 mL/min). Product containing samples were united and the solvents were evaporated. 482 mg (100% purity, 64% yield) of the title compound were obtained.

LC-MS (Method 3): *R*_t_ = 2.62 min; MS (ESIpos): *m*/*z* = 632 [M + H]^+^.

#### [4-(7-{[6-(Methylamino)hexyl]oxy}pyrido[3,2-*d*]pyrimidin-4-yl)piperidin-1-yl][4-(trifluoromethoxy)phenyl]methanone Hydrochloride

*tert*-Butyl methyl{6-[(4-{1-[4-(trifluoromethoxy)benzoyl]piperidin-4-yl}pyrido[3,2-*d*]pyrimidin-7-yl)oxy]hexyl}carbamate (482 mg, 0.76 mmol) was dissolved in 10 mL dichloromethane. Hydrochloric acid in 1,4-dioxan (1.91 mL, 4 N, 7.63 mmol) was added to the mixture and the mixture was stirred at room temperature for 1.5 h. The solvents were evaporated, and the residue was dried in vacuo. 495 mg (93% purity, 106% yield) of the title compound were obtained.

LC-MS (Method 1): *R*_t_ = 1.24 min; MS (ESIpos): *m*/*z* = 532 [M + H]^+^.

#### Methyl *N*-Methyl-N-{6-[(4-{1-[4-(trifluoromethoxy)benzoyl]piperidin-4-yl}pyrido[3,2-*d*]pyrimidin-7-yl)oxy]hexyl}glycinate

To a mixture of [4-(7-{[6-(methylamino)hexyl]oxy}pyrido[3,2-*d*]pyrimidin-4-yl)piperidin-1-yl][4-(trifluoromethoxy)phenyl]methanone hydrochloride (445 mg, 93% purity, 0.74 mmol) were added methyl chloroacetate (70 μL, 0.74 mmol) and cesium carbonate (720 mg, 2.21 mmol) and the mixture was stirred at 60 °C for 5 h. The mixture was poured into water and extracted with ethyl acetate. The combined organic layers were washed with water and brine, dried over anhydrous sodium sulfate, and concentrated in vacuo. The residue was purified by column chromatography (Machine: Biotage Isolera; column: Biotage Sfär HC 50 g; eluent: DCM/MeOH: 2% MeOH → 20% MeOH; flow: 120 mL/min). Product containing samples were united and the solvents were evaporated. 320 mg (100% purity, 72% yield) of the title compound were obtained.

LC-MS (Method 1): *R*_t_ = 1.27 min; MS (ESIpos): *m*/*z* = 604 [M + H]^+^.

#### *N*-Methyl-*N*-{6-[(4-{1-[4-(trifluoromethoxy)benzoyl]piperidin-4-yl}pyrido[3,2-*d*]pyrimidin-7-yl)oxy]hexyl}glycine

To a solution of methyl *N*-methyl-*N*-{6-[(4-{1-[4-(trifluoromethoxy)benzoyl]piperidin-4-yl}pyrido[3,2-*d*]pyrimidin-7-yl)oxy]hexyl}glycinate (320 mg, 530 μmol) in 10 mL THF was added a lithium hydroxide solution (1.06 mL, 1 M, 1.06 mmol). The mixture was stirred at 60 °C for 2 h. The solvent was evaporated, and the residue was diluted with water and acidified with 2 N HCl. The mixture was extracted with ethyl acetate. The combined organic layers were dried over anhydrous sodium sulfate and filtered. The filtrate was concentrated in vacuo. 442 mg (79% purity, 111% yield) of the title compound were obtained.

LC-MS (Method 1): *R*_t_ = 1.44 min; MS (ESIpos): *m*/*z* = 590 [M + H]^+^.

#### *N*-Methyl-*N*-{6-[(4-{1-[4-(trifluoromethoxy)benzoyl]piperidin-4-yl}pyrido[3,2-*d*]pyrimidin-7-yl)oxy]hexyl}glycyl-3-methyl-l-valyl-(4*R*)-4-hydroxy-*N*-[4-(4-methyl-1,3-thiazol-5-yl)benzyl]-l-prolinamide (**7**)

To a solution of *N*-methyl-*N*-{6-[(4-{1-[4-(trifluoromethoxy)benzoyl]piperidin-4-yl}pyrido[3,2-*d*]pyrimidin-7-yl)oxy]hexyl}glycine (295 mg, 79% purity, 0.5 mmol) in 5 mL DMF were added 3-methyl-l-valyl-(4*R*)-4-hydroxy-*N*-[4-(4-methyl-1,3-thiazol-5-yl)benzyl]-l-prolinamide (215 mg, 0.5 mmol), HATU (209 mg, 0.55 mmol) and *N*,*N*-diisopropylethylamine (0.26 mL, 1.5 mmol) and the mixture was stirred at room temperature overnight. The reaction mixture was poured into water and extracted with ethyl acetate. The combined organic layers were washed with brine, dried over anhydrous sodium sulfate, filtered, and concentrated in vacuo. The residue was purified by preparative HPLC (Column: Chromatorex C18 10 μm 250 mm × 30 mm; Eluent A = water, B = acetonitrile; gradient: 0.0 min 30% B; 4.5 min 50% B; 11.5 min 70% B; 12 min 100% B; 14.75 min 30% B; flow: 50 mL/min; 0.1% formic acid). Product containing samples were united and the solvents were evaporated. 7 mg (100% purity, 1.4% yield) of the title compound were obtained.

LC-MS (Method 2): *R*_t_ = 3.70 min; MS (ESIpos): *m*/*z* = 1002 [M + H]^+^.

NMR: See Supporting Information.

### Preparation of PROTAC **8** and Respective Starting Materials

#### *tert*-Butyl 4-(3-iodopropyl)piperidine-1-carboxylate

To a mixture of triphenylphosphine (1.29 g, 4.93 mmol), 1H-imidazole (420 mg, 6.16 mmol) and iodine (1.25 g, 4.93 mmol) in 10 mL of THF was added *tert*-butyl 4-(3-hydroxypropyl)piperidine-1-carboxylate (1.0 g, 4.11 mmol). The mixture was stirred at room temperature overnight. The solid was filtered off, washed with ethyl acetate and the filtrate was concentrated. The residue was purified by column chromatography (Machine: Biotage Isolera; column: Biotage Sfär HC 25 g; eluent: Cy/EE: 5% EE → 20% EE; flow: 80 mL/min). Product containing samples were united and the solvents were evaporated. 880 mg (100% purity, 61% yield) of the title compound were obtained.

LC-MS (Method 1): *R*_t_ = 2.47 min; MS (ESIpos): *m*/*z* = 298 [M + H – C_4_H_8_]^+^.

#### *tert*-Butyl 4-{3-[(4-{1-[4-(trifluoromethoxy)benzoyl]piperidin-4-yl}pyrido[3,2-*d*]pyrimidin-7-yl)oxy]propyl}piperidine-1-carboxylate

To a mixture of *tert*-butyl 4-(3-iodopropyl)piperidine-1-carboxylate (507 mg, 1.43 mmol) and potassium carbonate (330 mg, 2.39 mmol) in 5 mL DMF was added [4-(7-hydroxypyrido[3,2-*d*]pyrimidin-4-yl)piperidin-1-yl][4-(trifluoromethoxy)phenyl]methanone^20^ (500 mg, 1.19 mmol) and the mixture was stirred at room temperature overnight. The mixture was poured into water and extracted with ethyl acetate. The combined organic layers were washed with water and brine, dried over anhydrous sodium sulfate, and concentrated in vacuo. The residue was purified by column chromatography (Machine: Biotage Isolera; column: Biotage Sfär HC 25 g; eluent: Cy/EE: 12% EE → 100% EE; flow: 80 mL/min). Product containing samples were united and the solvents were evaporated. 685 mg (100% purity, 89% yield) of the title compound were obtained.

LC-MS (Method 3): *R*_t_ = 2.63 min; MS (ESIpos): *m*/*z* = 644 [M + H]^+^.

#### (4-{7-[3-(Piperidin-4-yl)propoxy]pyrido[3,2-*d*]pyrimidin-4-yl}piperidin-1-yl)[4-(trifluoromethoxy)phenyl]methanone Hydrochloride

*tert*-Butyl 4-{3-[(4-{1-[4-(trifluoromethoxy)benzoyl]piperidin-4-yl}pyrido[3,2-*d*]pyrimidin-7-yl)oxy]propyl}piperidine-1-carboxylate (685 mg, 1.06 mmol) was dissolved in 10 mL dichloromethane. Hydrochloric acid in 1,4-dioxan (2.66 mL, 4 N, 1.64 mmol) was added and the mixture was stirred at room temperature for 1.5 h. The solvents were evaporated, and the residue was dried in vacuo. 616 mg (94% purity, 93% yield) of the title compound were obtained.

LC-MS (Method 1): *R*_t_ = 1.26 min; MS (ESIpos): *m*/*z* = 544 [M + H]^+^.

#### Methyl (4-{3-[(4-{1-[4-(Trifluoromethoxy)benzoyl]piperidin-4-yl}pyrido[3,2-*d*]pyrimidin-7-yl)oxy]propyl}piperidin-1-yl)acetate

To a mixture of (4-{7-[3-(piperidin-4-yl)propoxy]pyrido[3,2-*d*]pyrimidin-4-yl}piperidin-1-yl)[4-(trifluoromethoxy)phenyl]methanone hydrochloride (566 mg, 94% purity, 0.92 mmol) were added methyl chloroacetate (80 μL, 0.92 mmol) and cesium carbonate (897 mg, 2.75 mmol) and the resulting mixture was stirred at 60 °C for 5 h. The mixture was poured into water and extracted with ethyl acetate. The combined organic layers were washed with water and brine, dried over anhydrous sodium sulfate, and concentrated in vacuo. The residue was purified by column chromatography (Machine: Biotage Isolera; column: Biotage Sfär HC 50 g; eluent: DCM/MeOH: 2% MeOH → 20% MeOH; flow: 120 mL/min). Product containing samples were united and the solvents were evaporated. 443 mg (100% purity, 78% yield) of the title compound were obtained.

LC-MS (Method 1): *R*_t_ = 1.25 min; MS (ESIpos): *m*/*z* = 616 [M + H]^+^.

#### (4-{3-[(4-{1-[4-(Trifluoromethoxy)benzoyl]piperidin-4-yl}pyrido[3,2-*d*]pyrimidin-7-yl)oxy]propyl}piperidin-1-yl)acetic Acid

To a solution of methyl (4-{3-[(4-{1-[4-(trifluoromethoxy)benzoyl]piperidin-4-yl}pyrido[3,2-*d*]pyrimidin-7-yl)oxy]propyl}piperidin-1-yl)acetate (443 mg, 720 μmol) in 10 mL THF was added a lithium hydroxide solution (1.44 mL, 1 M, 1.44 mmol). The mixture was stirred 60 °C for 2 h. The solvent was evaporated, and the residue was diluted with water and acidified with 2 N HCl. The mixture was extracted with ethyl acetate. The combined organic layers were dried over anhydrous sodium sulfate and filtered. The filtrate was concentrated in vacuo. 1.12 g of a product-salt mixture was obtained and used in the next step without prior purification.

LC-MS (Method 1): *R*_t_ = 1.42 min; MS (ESIpos): *m*/*z* = 602 [M + H]^+^.

#### 3-Methyl-*N*-[(4-{3-[(4-{1-[4-(trifluoromethoxy)benzoyl]piperidin-4-yl}pyrido[3,2-*d*]pyrimidin-7-yl)oxy]propyl}piperidin-1-yl)acetyl]-l-valyl-(4*R*)-4-hydroxy-*N*-[4-(4-methyl-1,3-thiazol-5-yl)benzyl]-l-prolinamide (**8**)

To a solution of (4-{3-[(4-{1-[4-(trifluoromethoxy)benzoyl]piperidin-4-yl}pyrido[3,2-*d*]pyrimidin-7-yl)oxy]propyl}piperidin-1-yl)acetic acid (1.12 g product-salt mixture, containing 0.7 mmol educt) in 5 mL DMF were added 3-methyl-l-valyl-(4*R*)-4-hydroxy-*N*-[4-(4-methyl-1,3-thiazol-5-yl)benzyl]-l-prolinamide (301 mg, 0.7 mmol), HATU (292 mg, 0.77 mmol) and *N*-ethyl-*N*-isopropylpropan-2-amine (0.37 mL, 2.1 mmol) and the mixture was stirred at room temperature overnight. The reaction mixture was poured into water and extracted with ethyl acetate. The combined organic layers were washed with brine, dried over anhydrous sodium sulfate, filtered, and concentrated in vacuo. The residue was purified by preparative HPLC (Column: Chromatorex C18 10 μm 250 mm × 30 mm; Eluent A = water, B = acetonitrile; gradient: 0.0 min 30% B; 4.5 min 50% B; 11.5 min 70% B; 12 min 100% B; 14.75 min 30% B; flow: 50 mL/min; 0.1% formic acid). Product containing samples were united and the solvents were evaporated. 14 mg (100% purity, 2% yield) of the title compound were obtained.

LC-MS (Method 2): *R*_t_ = 3.66 min; MS (ESIpos): *m*/*z* = 1014 [M + H]^+^.

HRMS (ESI) *m*/*z*: calcd. for C_52_H_63_N_9_O_7_F_3_S [M + H]^+^, 1014.4523; found, 1014.4539.

NMR: See Supporting Information.

### Aqueous Solubility and Log *D*

The kinetic aqueous solubility at pH 6.5 and chromatographic Log *D* at pH 7.5 (Log *D*_7.5_) of **1**–**9** were determined as reported previously.^56^

### p*K*_a_

The p*K*_a_ of **7** was determined by photometry as reported previously.^57^

### Biochemical VHL Binding Assay

The complex of VHL protein, Elongin B, and Elongin C protein (VBC complex) was labeled with an amine reactive (isothiocyanate group) terbium chelate. A HIF-1alpha derived peptide with hydroxyproline was labeled with fluorescein on the C terminus (H-DLDLEMLA-Hyp-YIPMDDDFQL-Lys(FAM)-NH_2_). The hydroxyproline is essential for the binding of the peptide to the VBC complex, leading to an energy transfer from terbium to fluorescein and the generation of FRET signals (ex 340 nm, em 520 nm). The decrease in the FRET signal is proportional to the binding of a compound to VHL.

Serial dilutions of test compounds were prepared in DMSO. Compounds were tested in duplicates at 10 concentrations (30 μM, 10 μM, 3.3 μM, 1.1 μM, 0.37 μM, 0.12 μM, 41 nM, 14 nM, 5 nM, 2 nM). Buffer composition: 50 mM Hepes, pH 7.5, 1.5 mM MgCl_2_, 5 mM KCl, 0.001% Brij, 0.001% BSA, and 1 mM DTT. The assay was performed in a white 384 well microtiter plate (Greiner, Germany) with 25 μL of VBC complex (final concentration: 5 nM), 1 μL of compound dilution, and 25 μL of peptide (final concentration: 2.5 nM). The signal was measured for 90 min in a PHERAstar reader (BMG, Germany) at room temperature. IC_50_ values are determined by interpolation from plots of relative FRET signal versus inhibitor concentration. In order to ensure that IC_50_ values were not affected by the precipitation of poorly soluble test compounds, microtiter plates were inspected visually during the assay, then the shape of the inhibition curves was also examined.

### Cell-Based VHL Binding Assay

Live cells (live mode) using the In-cell VHL Kit from Promega (Promega, USA) were used to assess VHL target engagement as well as cell permeability. Serial dilutions of test compounds were prepared in DMSO at 100× the final assay concentration using a Precision Pipetting System (BioTek, USA, the resulting final concentrations are given at the end of the assay description). The dilution series of the test compound in DMSO (50 nL of each concentration) was transferred to white nonbinding microtiter test plates (384, Corning, USA) using a Hummingbird liquid handler (Digilab, MA, USA). Plates were sealed with adhesive foil and stored at −20 °C until use. Cells overexpressing NanoLuc-VHL (600 cells/well) were diluted in Optimem without phenol red and plated in the assay plates containing the dilution series of the test compounds. VHL tracer (1 μM final concentration for live mode) was added. The live mode assay was incubated for 2 h at 37 °C, 5% CO_2_. Immediately prior to BRET measurements, NanoBRET NanoGlo substrate was prepared in Optimem without phenol red (10 μM final concentration), additionally for the live mode the NanoGlo extracellular inhibitor was added at a final concentration of 20 μM (Promega, USA). Donor emission (450 nm) and acceptor emission (610 or 630 nm) was recorded after 3 min of substrate addition with a PHERAstar reader (BMG, Germany) using a NanoBRET module (Luminescence Module 450–610). The ratio of the emissions at 610 and 450 nm was used as the specific signal for further evaluation. The data were normalized using the controls: No Tracer = 100% displacement, control wells with tracer = 0% displacement. Compounds were tested in duplicates at up to 11 concentrations (e.g., 50 μM, 20 μM, 5.7 μM, 1.6 μM, 0.47 μM, 0.13 μM, 38 nM, 11 nM, 3.1 nM, 0.89 nM, and 0.25 nM). IC_50_ values were calculated using a four-parameter fit, with a commercial software package (Genedata Screener, Switzerland). In order to ensure that IC_50_ values were not affected by the precipitation of poorly soluble test compounds, microtiter plates were inspected visually during the assay, then the shape of the inhibition curves was also examined.

### PAMPA Permeability

The permeabilities of **1**–**9** in the parallel artificial membrane permeability assay (PAMPA) was determined at Pharmaron.^58^ Methotrexate (Log *P*_e_ −8.37) and testosterone (Log *P*_e_ −4.24) were used as controls. Stock solutions of the controls and of **1**–**9** were prepared in DMSO at a concentration of 10 mM, then they were further diluted with PBS (pH 7.4) to a final concentration of 10 μM. Five μL of a lecithin/dodecane mixture and 300 μL of PBS (pH 7.4) solution were pipetted into each acceptor plate well, followed by addition of 300 μL of the solutions of the controls or **1**–**9** to each well of the donor plate. The acceptor plate was placed into the donor plate followed by incubation at 25 °C, 60 rpm for 16 h.^31^ After incubation, aliquots of 50 μL from each well of the acceptor and donor plates are transferred into a 96-well plate and 200 μL of acetonitrile was added to each well. Samples were centrifuged for 20 min and compound concentrations were determined by LC/MS/MS (LC system: Shimadzu; Triple Quad).

### NMR Spectroscopy

The NMR spectra of PROTACs **1**, **2**, **3**, **6**, and **7** were recorded in CDCl_3_ on an 800 MHz Bruker Avance Neo NMR spectrometer equipped with a TXI cryogenic probe. The compounds were assigned based on ^1^H, TOCSY, COSY, HSQC, HMBC, and NOESY NMR spectra. Spectra were recorded at 25 °C for PROTACs **3**, **6**, and **7**, at −25 °C for PROTACs **1**, **2**, **3**, and **7** and at −20 °C for PROTAC **6**. The ^1^H and ^13^C NMR chemical shift assignments are listed in Tables S1–S5.

NOESY experiments were recorded with different mixing times (100, 200, 300, 400, 500, 600, and 700 ms). All spectra were recorded with 16 scans with 8192 and 512 Hz data points in the direct (F2) and indirect (F1) dimensions, and with a relaxation delay of 2.5 s. The spectra were processed using the MestReNova version 14.3.0 software. The NOE peak intensities were normalized according to ([cross peak1 × cross peak2]/[diagonal peak1 × diagonal peak2])^0.5^.^39^ The initial rate approximation^59^ was used to calculate interproton distances. A minimum of three mixing times with *R*^2^ ≥ 0.91 for the build-up rate (σ_ab_) were required for distance calculation and further NAMFIS analysis. Interproton distances were calculated based on the NOE build-up rates using the equation , where *r*_ab_ is the distance between protons a and b, *r*_ref_ is the distance between two germinal methylene protons that are used as a reference (distance of 1.78 Å), and σ_ref_ and σ_ab_ are the slope of the NOE build-up curve for the reference and a, b protons. The interproton distances for the NAMFIS analysis are summarized in Tables S6–S10.

### Theoretical Conformational Ensembles

To ensure that the entire conformational space available for the PROTACs was sampled, ten different unrestrained Monte Carlo Multiple Minimum (MCMM) conformational searches were set up for each PROTAC using five different force fields (AMBER*, MMFF, OPLS, OPLS4, and OPLS2005), each using the GB/SA implicit solvation models for both chloroform and water. All conformational searches for each PROTAC were performed with the same input structure, after which each conformation was minimized using the Polak-Ribiere Conjugate Gradient (PRCG) with a maximum of 5000 iterations as implemented in the batchmin algorithm of MacroModel v 13.6 (Schrödinger Release 2023-3, MacroModel, Schrödinger, LLC, New York, NY, 2023).^60,61^ The conformational searches were performed with intermediate torsion sampling, a 42.0 kJ/mol energy window for saving structures, an RMSD cutoff of 2.0 Å, and 50 000 maximum number of steps. The number of torsion angles allowed to vary during each Monte Carlo step ranged from 1 to *n* – 1 where n equals the total number of rotatable bonds. The conformational searches fulfilled the eq 1 – (1 – (1/*N*))*^M^* as an estimate of the probability that the conformational search is complete, where *N* is the total number of conformers and *M* is the number of search steps. All of the 10 conformational searches obtained from the MCMM conformational search for each PROTAC were combined and identical conformations were removed by performing redundant conformational elimination (RCE) using an RMSD cutoff of 3.0 Å to obtain a final ensemble for the NAMFIS analysis.

### NAMFIS Analysis

The conformational ensembles of the PROTACs were determined using the NAMFIS algorithm by fitting population-weighted, back-calculated interproton distances from conformations in the theoretical ensembles to those experimentally determined, as described previously.^36^ Methylene (CH_2_) signals were treated according to the equation *d* = ((*d*_1_^–6^) + (*d*_2_^–6^))/2^–1/6^, and methyl (CH_3_) signals according to *d* = (((*d*_1_^–6^) + (*d*_2_^–6^) + (*d*_3_^–6^))/3)^−1/6^. The output conformational ensembles were first validated by comparison of the experimentally observed and back-calculated distances in terms of RMSD (Tables S16 and S17). Additional validation involved detection of no significant change in the ensembles by the addition of up to 5% random noise to the experimental data, and upon random removal of individual experimental restraints.

### Molecular Descriptors Calculations

The radius of gyration (*R*_gyr_) and the solvent-accessible three-dimensional polar surface area (SA 3D PSA) of the different solution conformations (Table S18) was calculated using PyMol (version 2.5.0) with a solvent probe radius of 1.4 Å and a partial charge threshold of >1.0, as previously described.^62^ The SA 3D PSA of the linker, and the ERK5 and VHL ligands in selected conformations was calculated from the solvent accessible polar surface area calculated for all polar atoms (O, N, and attached H). Intramolecular interactions, such as intramolecular hydrogen bonds (IMHBs), π–π interactions and NH–π interactions, were calculated using the Maestro module from the Schrödinger suite (version 2023-4). All plots for the property analyses of the solution ensembles were created using the RStudio (version 1.3.959) and PyMol (version 2.5.0) tools.

## Data Availability

Original NMR spectra (FIDs) and the structure files for the ensembles of PROTACs **1**, **2**, **3**, **6** and **7** are available free of charge at Zenodo: https://doi.org/10.5281/zenodo.12805072.

## Supporting Information Available

HPLC purity analyses, HRMS and ^1^H NMR spectra for PROTACs **1**–**8**; physicochemical property correlations for PROTACs **1**–**9**; 800 MHz ^1^H NMR data, Monte Carlo molecular mechanics conformational searches, NAMFIS conformational analyses and characterization of the resulting solution ensembles for **2**, **3**, **6** and **7** (PDF)
Molecular formula strings document with structures (SMILES codes), aqueous solubility, chromatographic LogD, and permeability data for **1**–**9** SI-2_MFS_VHL_Rev241129 (CSV)

## Abbreviations Used

bRo5: beyond rule of 5
CRBN: cereblon
ERK5: extracellular signal-regulated kinase 5
IMHB: intramolecular hydrogen bond
MD: molecular dynamics
NAMFIS: NMR analysis of molecular flexibility in solution
NRotB: number of rotatable bonds
PAMPA: parallel artificial membrane permeability assay
POI: protein of interest
PROTAC: proteolysis targeting chimera
R_gyr_: radius of gyration
Ro5: Lipinski′s rule of 5
PSA: polar surface area
SA 3D PSA: solvent accessible 3D polar surface area
TPSA: topological polar surface area
VHL: von Hippel-Lindau
