# Supplementary material for: Impact of Linker Composition on VHL PROTAC Cell Permeability
Source: J Med Chem. 2024 Dec 18;68(1):638–57. doi: 10.1021/acs.jmedchem.4c02492 (PMC11726670; doi:10.1021/acs.jmedchem.4c02492)
Supplement: Supplementary file 1 — jm4c02492_si_001.pdf [file jm4c02492_si_001.pdf]

SUPPORTING INFORMATION

Impact of Linker Composition on VHL PROTAC Cell Permeability

Yordanos Esubalew Abeje,[a] Lianne H. E. Wieske,[a] Vasanthanathan Poongavanam,[a] Stefanie Maassen,[b] Yoseph Atilaw,[a] Philipp Cromm,[b] Lutz Lehmann,[b] Mate Erdelyi,[a] Daniel Meibom[b,*] and Jan Kihlberg[a,*]

[a] Department of Chemistry - BMC, Uppsala University, Box 576, 75123 Uppsala, Sweden

[b] Bayer AG, Drug Discovery Sciences, 42113 Wuppertal, Germany

Corresponding authors

Daniel Meibom, daniel.meibom@bayer.com, ORCID: 0000-0003-4978-9842

Jan Kihlberg, jan.kihlberg@kemi.uu.se, ORCID: 0000-0002-4205-6040

Contents

1. Characterization of the purity and identity of PROTACs **1**–**8**
2. Physicochemical property correlations for PROTACs **1**–**9**
3. ^{1}H-NMR assignments for PROTACs **1**, **2**, **3**, **6** and **7**
4. Interproton distances from NOE build-up curves for PROTACs **1**, **2**, **3**, **6** and **7**
5. Monte Carlo Molecular Mechanics (MCMM) conformational searches for PROTACs **2**, **3**, **6** and **7**
6. Conformational ensembles derived by NAMFIS-analysis for PROTACs **2**, **3**, **6** and **7**
7. R_{gyr} and SA 3D PSA for the conformational ensembles derived by NAMFIS-analysis for PROTACs **2**, **3**, **6** and **7**

1. Characterization of the purity and identity of PROTACs 1–8

Analytical Methods

NMR Spectroscopy: ^{1}H NMR spectra were recorded in solvents indicated below at RT with Bruker Avance spectrometers operating at 400 or 500 MHz. Chemical shifts are reported in ppm relative to tetramethylsilane (TMS) as an internal standard.

LC/MS-Methode 2: System MS: Waters TOF instrument; System UPLC: Waters Acquity I-CLASS; Column: Waters, HSST3, 2.1 x 50 mm, C18 1.8 µm; Eluent A: 1 l Water + 0.01% Formic acid; Eluent B: 1 l Acetonitrile + 0.01% Formic acid; Gradient: 0.0 min 2% B → 0.5 min 2% B → 7.5 min 95% B → 10.0 min 95% B; Oven: 50°C; Flow: 1.00 ml/min; UV-Detection: 210 nm

LC/MS-Methode 4: Instrument: SHIMADZU LCMS-2020 SingleQuad; Column: Chromolith@Flash RP-18E 25-2 MM; eluent A: water + 0.0375 vol % trifluoroaceticacid, eluent B: acetonitrile + 0.01875 vol % trifluoroacetic acid; gradient: 0-0.8 min, 5-95% B, 0.8-1.2 min 95% B; flow 1.5 ml/min; temperature: 50 °C; PDA: 220nm&254nm.

Single Mass Analysis (HR-MS): Instrument: Waters Time of Flight System (ToF), Electrospray Ionization (ESI).

Chromatograms and Spectra

3-methyl-N-{9-[(4-{1-[4-(trifluoromethoxy)benzoyl]piperidin-4-yl}pyrido[3,2-d]pyrimidin-7-yl)oxy]nonanoyl}-L-valyl-(4R)-4-hydroxy-N-[4-(4-methyl-1,3-thiazol-5-yl)benzyl]-L-prolinamide (**1**):

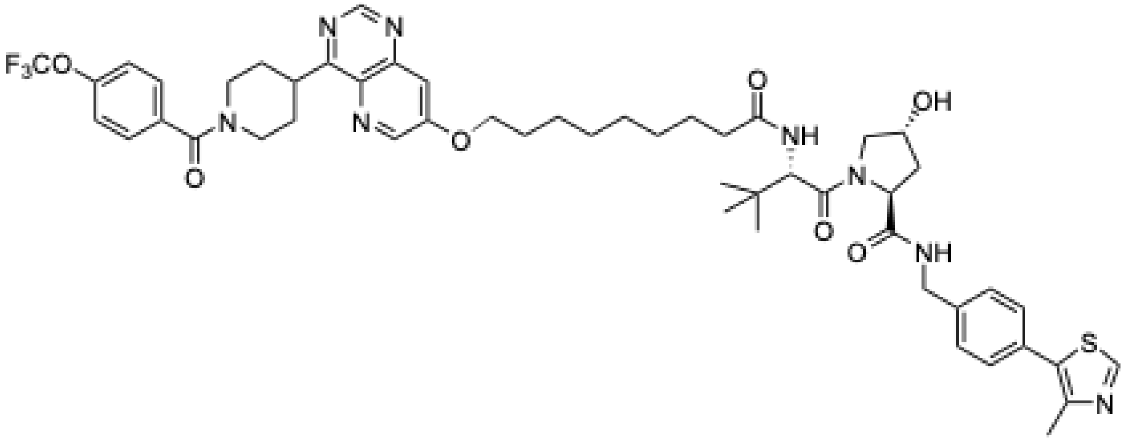

LC-MS (Method 4): R_t = 1.048 min; MS (ESIpos): m/z = 987 $[M-H]^+$

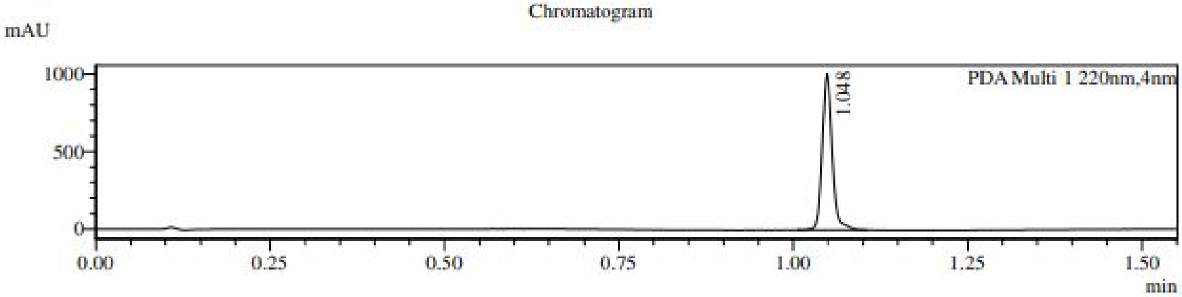

HRMS (ESI) m/z: calcd for $C_{51}H_{62}N_8O_7F_3S$ $[M+H]^+$, 987.4414; found, 987.4432

Single Mass Analysis

Tolerance = 2.0 PPM / DBE: min = -1.0, max = 50.0

Element prediction: Off

Number of isotope peaks used for i-FIT = 4

Monoisotopic Mass, Even Electron Ions

603 formula(e) evaluated with 1 results within limits (up to 50 best isotopic matches for each mass)

Elements Used:

C: 48-55 H: 20-100 N: 4-12 O: 4-12 F: 3-3 S: 0-1

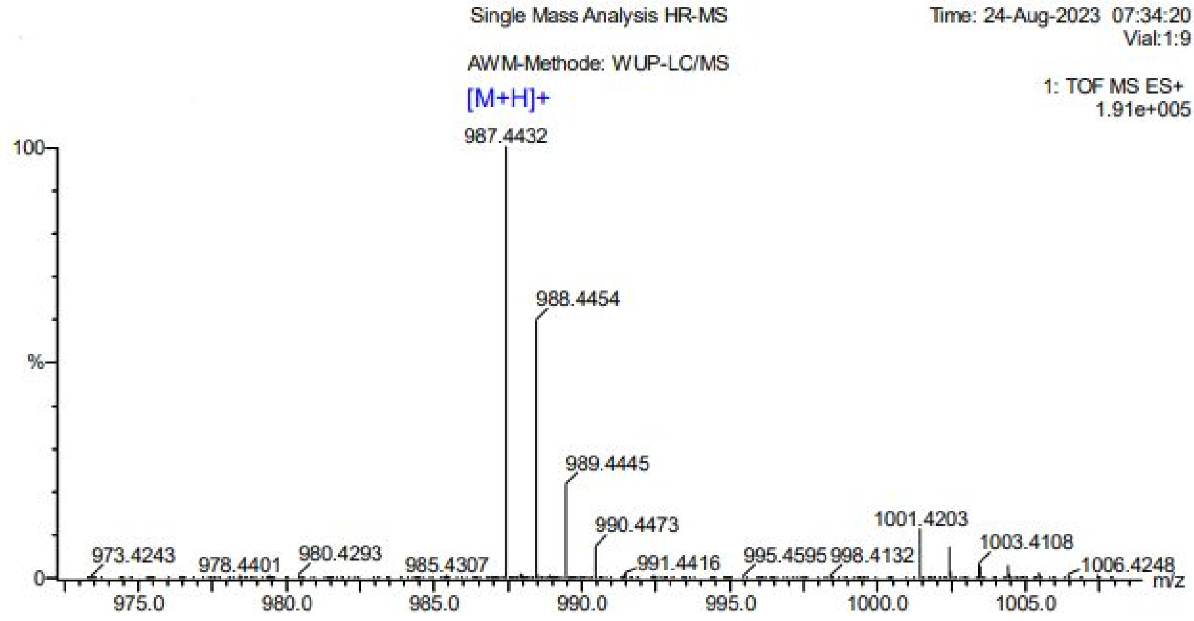

Minimum:				-1.0				
Maximum:		5.0	2.0	50.0				
Mass	Calc. Mass	mDa	PPM	DBE	i-FIT	Norm	Conf(%)	Formula
987.4432	987.4414	1.8	1.8	23.5	481.0	n/a	n/a	C51 H62 N8 O7 F3 S

Summenformelvorschlag

NMR (400 MHz, $CDCl_3$):

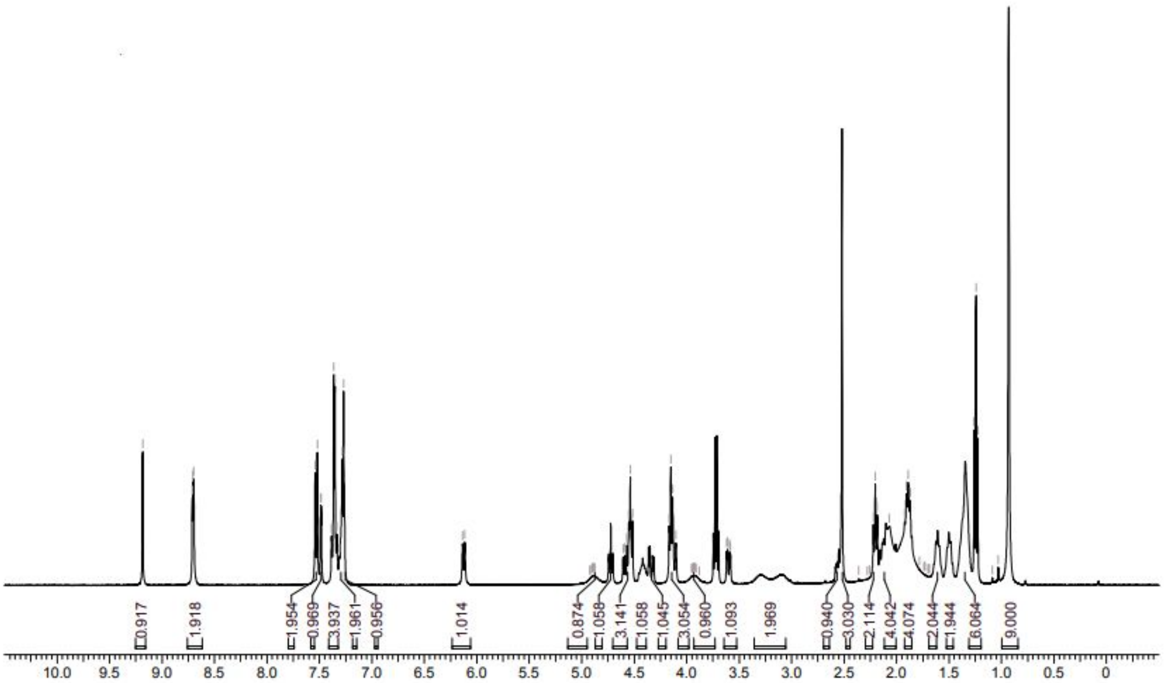

3-methyl-N-[3-(2-{2-[(4-{1-[4-(trifluoromethoxy) benzoyl]piperidin-4-yl}pyrido[3,2-d]pyrimidin-7-yl)oxy]ethoxy}ethoxy)propanoyl]-L-valyl-(4R)-4-hydroxy-N-[4-(4-methyl-1,3-thiazol-5-yl)benzyl]-L-prolinamide (**2**):

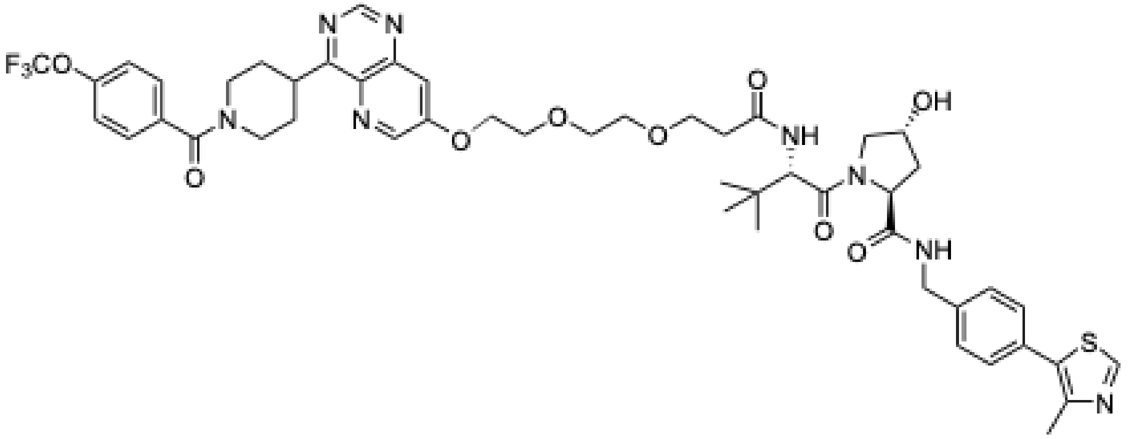

LC-MS (Method 2): R_t = 4.38 min; MS (ESIpos): m/z = 991 $[M+H]^+$

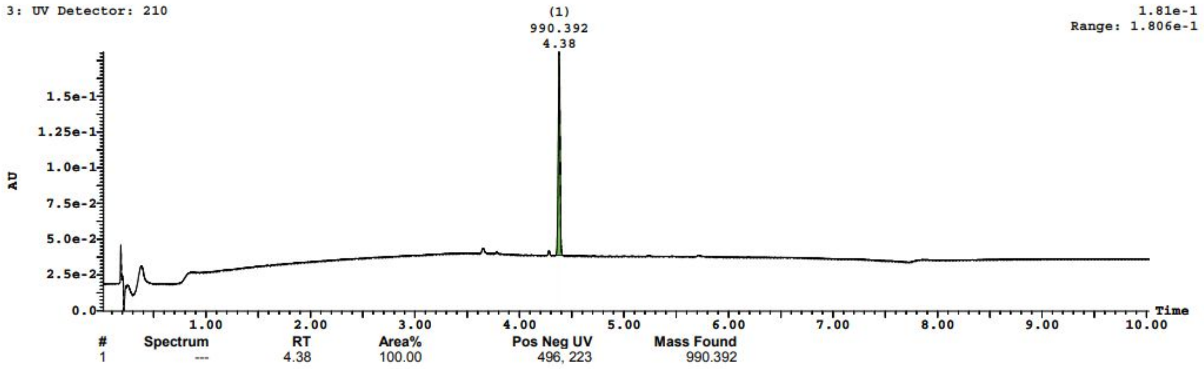

HRMS (ESI) m/z: calcd for $C_{49}H_{58}N_8O_9F_3S$ $[M+H]^+$, 991.4006; found, 991.4006

Single Mass Analysis
Tolerance = 2.0 PPM / DBE: min = -1.0, max = 30.0
Element prediction: Off
Number of isotope peaks used for i-FIT = 3

Monoisotopic Mass, Even Electron Ions
350 formula(e) evaluated with 1 results within limits (up to 50 closest results for each mass)
Elements Used:
C: 45-55 H: 10-100 N: 0-10 O: 0-10 F: 3-3 S: 1-1

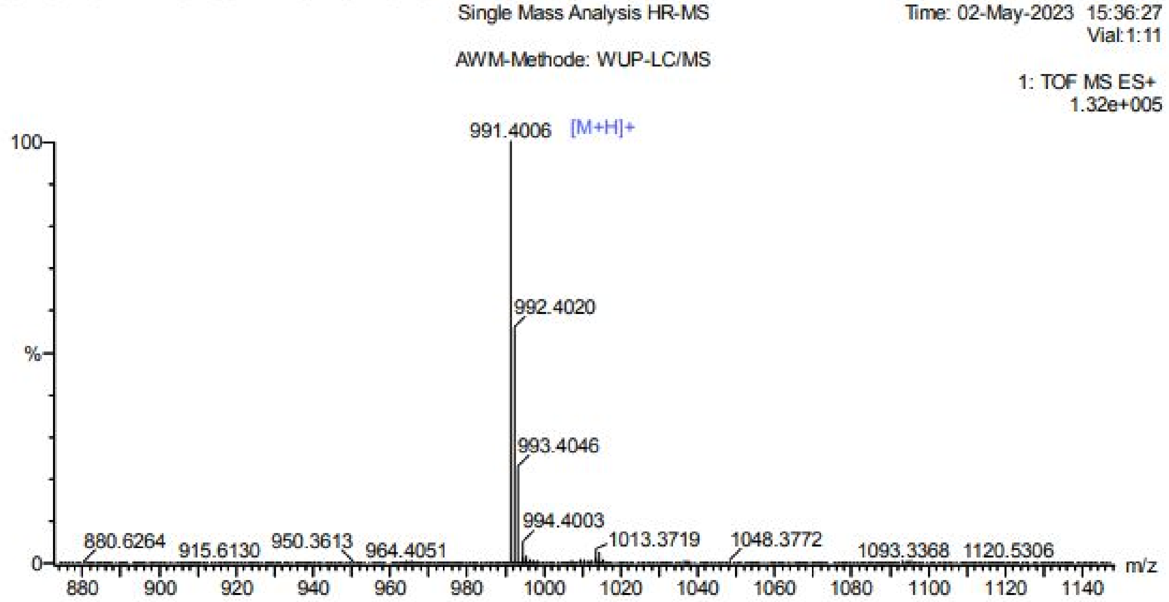

Minimum:				-1.0				
Maximum:		5.0	2.0	30.0				
Mass	Calc. Mass	mDa	PPM	DBE	i-FIT	Norm	Conf(%)	Formula
991.4006	991.4000	0.6	0.6	23.5	257.1	n/a	n/a	C49 H58 N8 O9 F3 S

Summenformelvorschlag

NMR (500 MHz, DMSO):

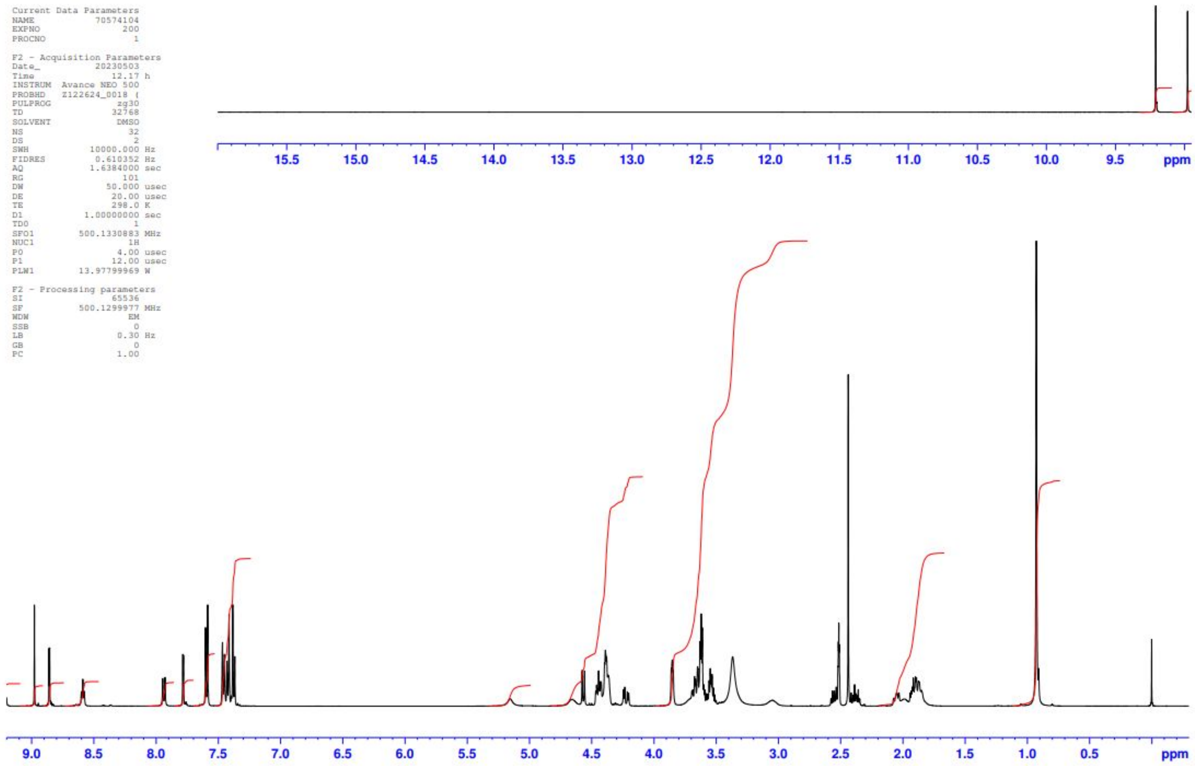

3-methyl-N-[3-(2-{2-[(4-{1-[4-(trifluoromethoxy)benzoyl]piperidin-4-yl}pyrido[3,2-d]pyrimidin-7-yl)oxy]acetamido}ethoxy)propanoyl]-L-valyl-(4R)-4-hydroxy-N-[4-(4-methyl-1,3-thiazol-5-yl)benzyl]-L-prolinamide (**3**):

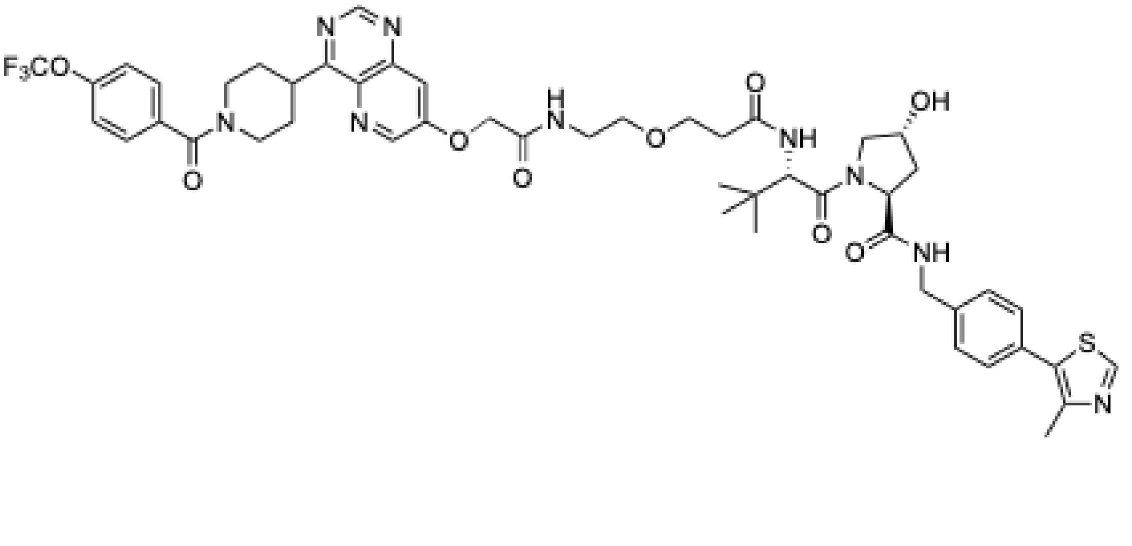

LC-MS (Method 2): R_t = 4.11 min; MS (ESIpos): m/z = 1004 [M+H]$^+$

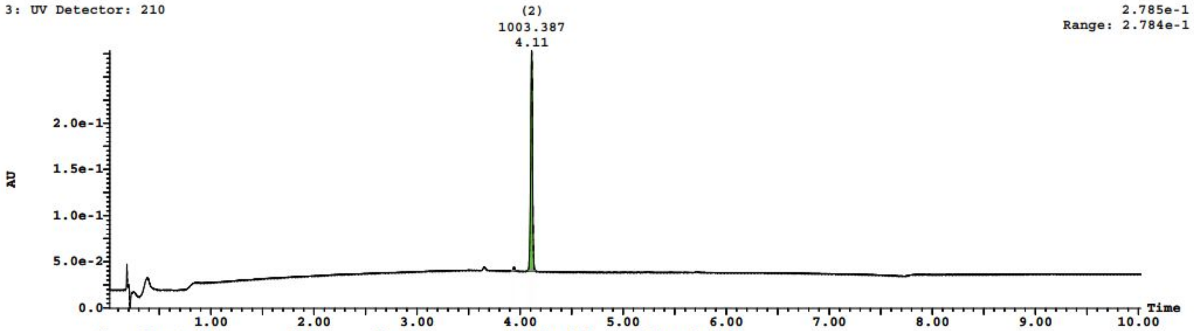

#	Spectrum	RT	Area%	Pos Neg UV	Mass Found
1	---	3.94	1.43	778, 221	
2	---	4.11	98.57	1004, 200	1003.387

HRMS (ESI) m/z: calcd for $C_{49}H_{57}N_9O_9F_3S$ [M+H]$^+$, 1004.3952; found, 1004.3947

Elemental Composition Report **Page 1**

Single Mass Analysis
Tolerance = 2.0 PPM / DBE: min = -1.0, max = 30.0
Element prediction: Off
Number of isotope peaks used for i-FIT = 3

Monoisotopic Mass, Even Electron Ions
415 formula(e) evaluated with 1 results within limits (up to 50 closest results for each mass)
Elements Used:
C: 45-55 H: 10-100 N: 0-12 O: 0-10 F: 3-3 S: 1-1

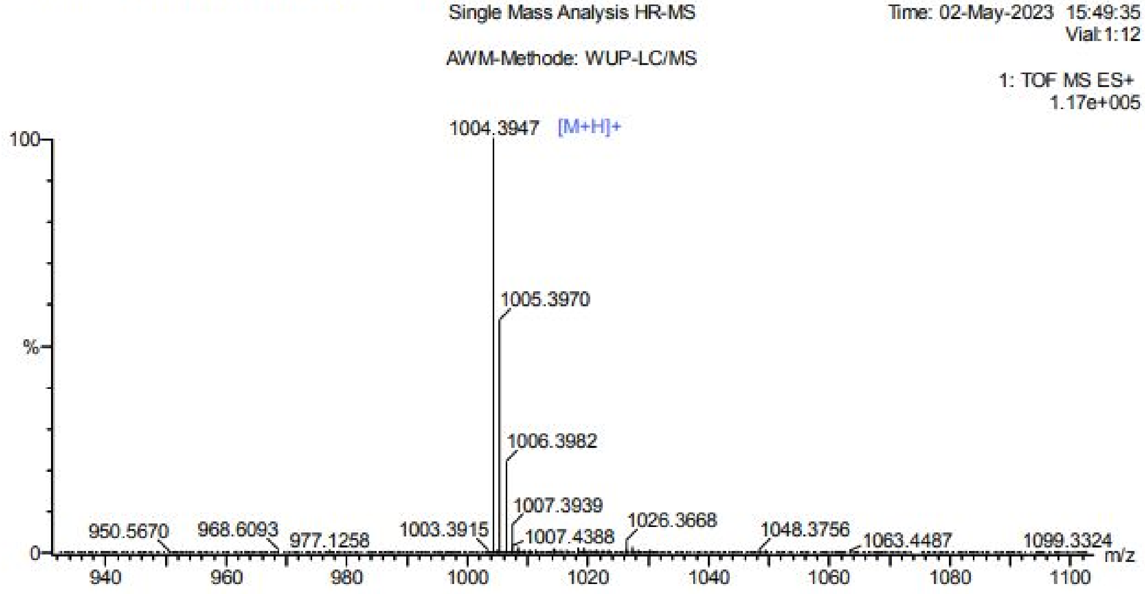

	Calc. Mass	mDa	PPM	DBE	i-FIT	Norm	Conf(%)	Formula
Minimum:				-1.0				
Maximum:		5.0	2.0	30.0				
**Mass**	**Calc. Mass**	**mDa**	**PPM**	**DBE**	**i-FIT**	**Norm**	**Conf(%)**	**Formula**
1004.3947	1004.3952	-0.5	-0.5	24.5	254.3	n/a	n/a	C49 H57 N9 O9 F3 S

Summenformelvorschlag

NMR (500 MHz, DMSO):

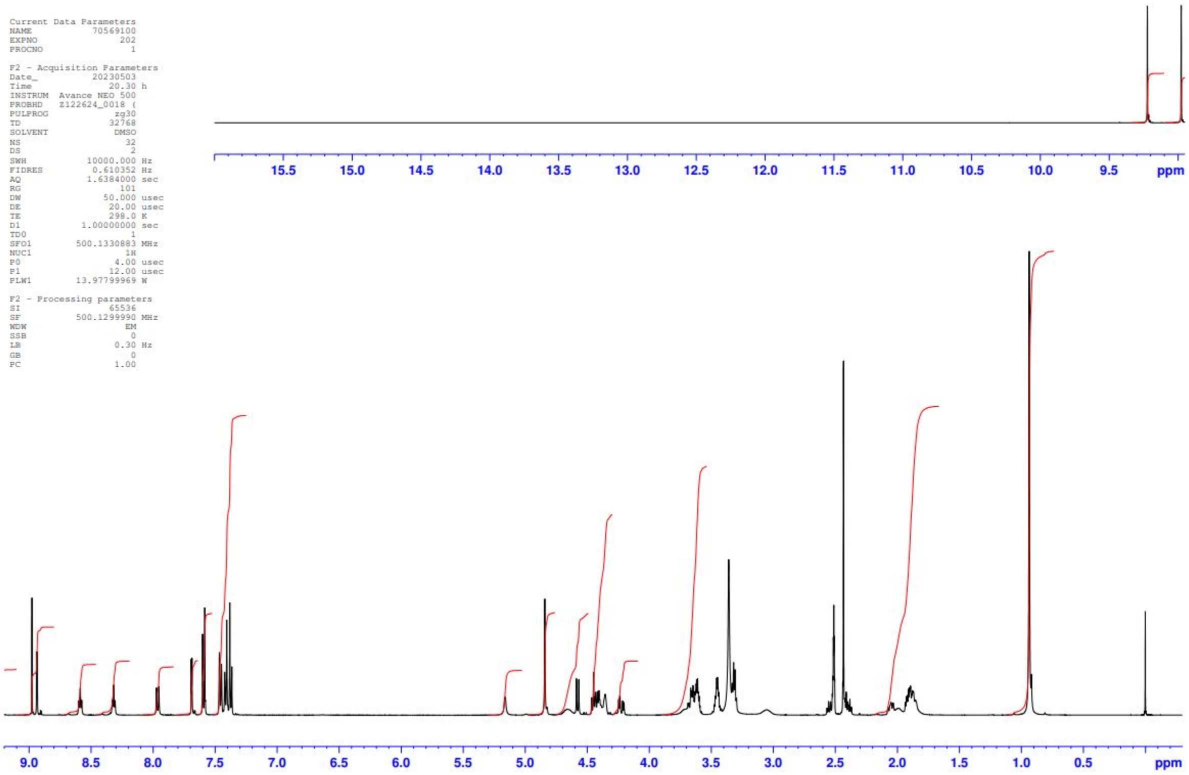

3-methyl-N-{[(1-{2-[(4-{1-[4-(trifluoromethoxy)benzoyl]piperidin-4-yl}pyrido[3,2-d]pyrimidin-7-yl)oxy]ethyl}-1H-1,2,3-triazol-4-yl)methoxy]acetyl}-L-valyl-(4R)-4-hydroxy-N-[4-(4-methyl-1,3-thiazol-5-yl)benzyl]-L-prolinamide (**4**):

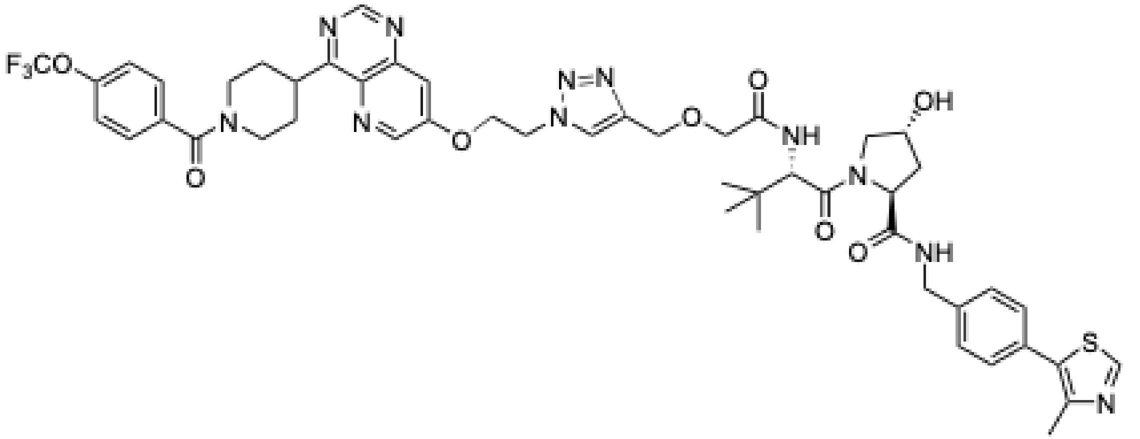

LC-MS (Method 2): R_t = 4.16 min; MS (ESIpos): m/z = 1014 $[M+H]^+$

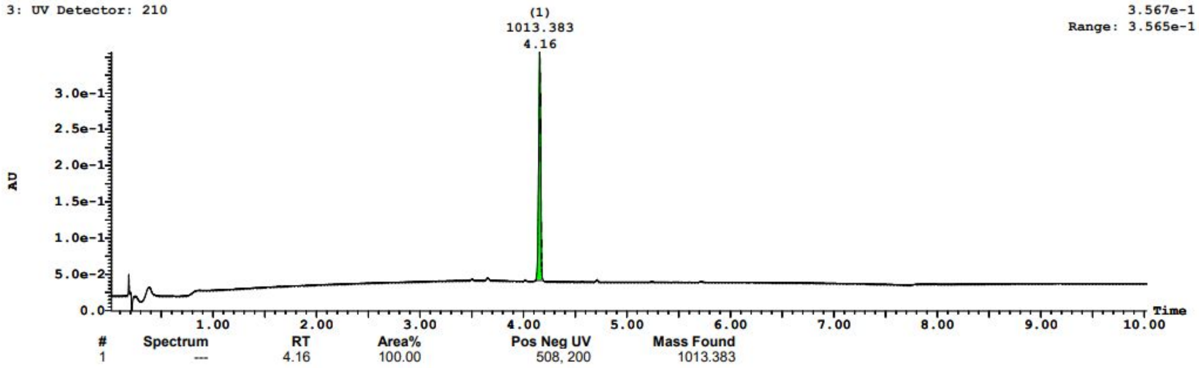

HRMS (ESI) m/z: calcd for $C_{49}H_{55}N_{11}O_8F_3S$ $[M+H]^+$, 1014.3908; found, 1014.3907

Elemental Composition Report **Page 1**

Single Mass Analysis
Tolerance = 2.0 PPM / DBE: min = -1.0, max = 30.0
Element prediction: Off
Number of isotope peaks used for i-FIT = 3

Monoisotopic Mass, Even Electron Ions
771 formula(e) evaluated with 1 results within limits (up to 50 closest results for each mass)
Elements Used:
C: 45-55 H: 10-100 N: 0-20 O: 0-10 F: 3-3 S: 1-1

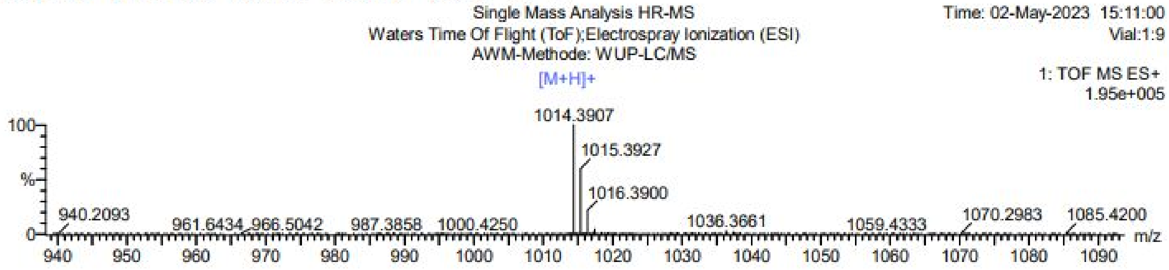

Minimum:				-1.0				
Maximum:		5.0	2.0	30.0				

Summenformelvorschlag

Mass	Calc. Mass	mDa	PPM	DBE	i-FIT	Norm	Conf(%)	Formula
1014.3907	1014.3908	-0.1	-0.1	26.5	309.9	n/a	n/a	C49 H55 N11 O8 F3 S

NMR (500 MHz, DMSO):

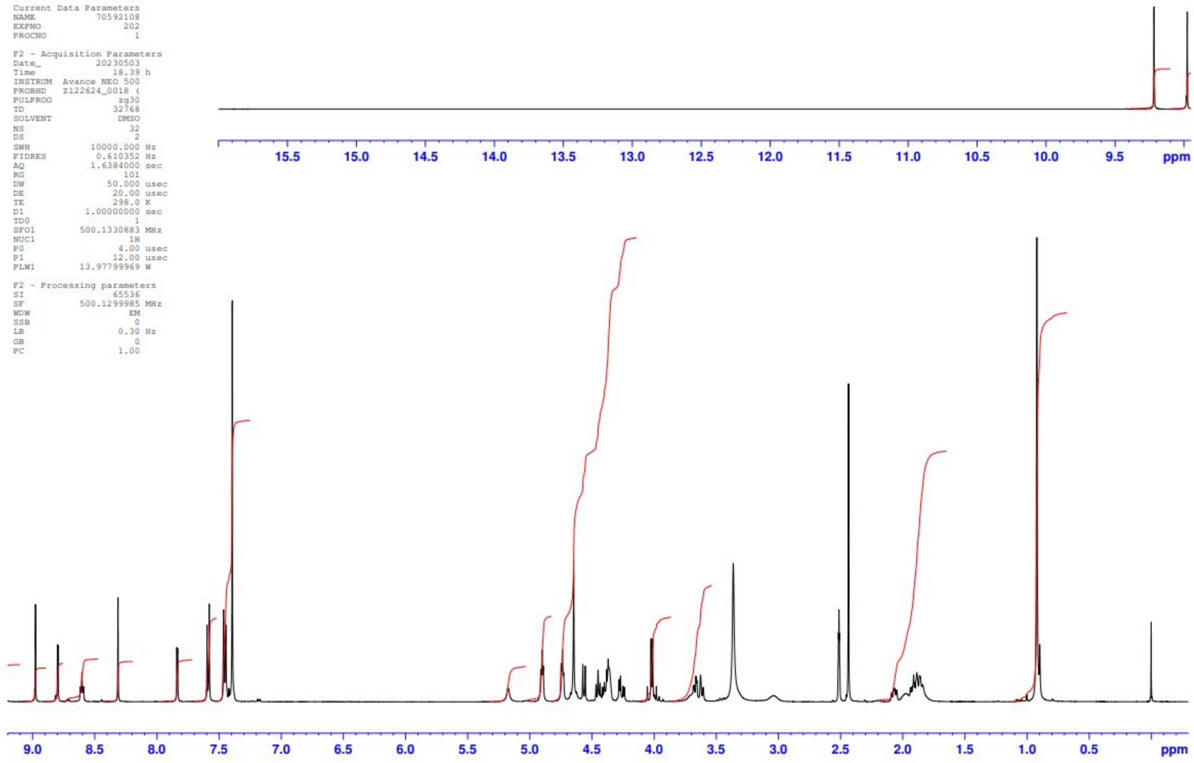

3-methyl-N-[(4-{2-[(4-{1-[4-(trifluoromethoxy)benzoyl]piperidin-4-yl}pyrido[3,2-d]pyrimidin-7-yl)oxy]ethoxy}phenyl)acetyl]-L-valyl-(4R)-4-hydroxy-N-[4-(4-methyl-1,3-thiazol-5-yl)benzyl]-L-prolinamide (**5**):

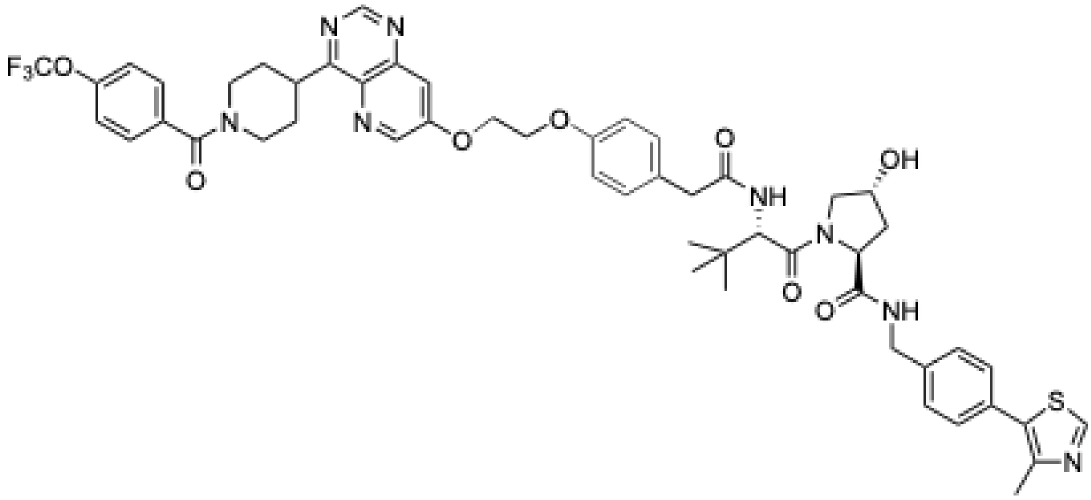

LC-MS (Method 2): R_t = 4.71 min; MS (ESIpos): m/z = 1009 $[M+H]^+$

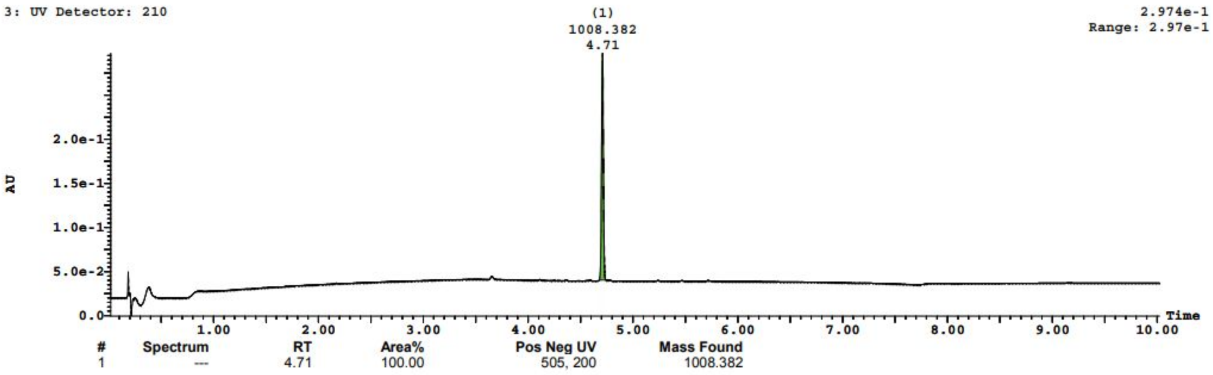

HRMS (ESI) m/z: calcd for $C_{52}H_{56}N_8O_8F_3S$ $[M+H]^+$, 1009.3894; found, 1009.3904

Elemental Composition Report

Page 1

Single Mass Analysis

Tolerance = 2.0 PPM / DBE: min = -1.0, max = 30.0
Element prediction: Off
Number of isotope peaks used for i-FIT = 3

Monoisotopic Mass, Even Electron Ions
797 formula(e) evaluated with 1 results within limits (up to 50 closest results for each mass)
Elements Used:
C: 45-55 H: 10-100 N: 0-20 O: 0-10 F: 3-3 S: 1-1

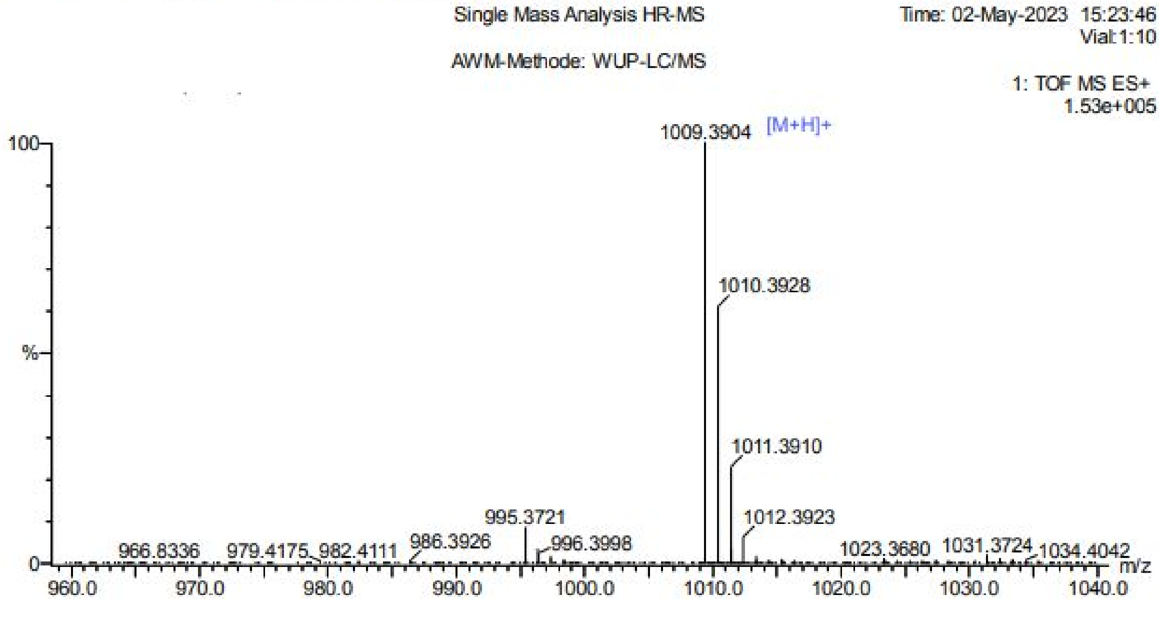

	Mass	Calc. Mass	mDa	PPM	DBE	i-FIT	Norm	Conf(%)	Formula
Minimum:					-1.0				
Maximum:			5.0	2.0	30.0				
	1009.3904	1009.3894	1.0	1.0	27.5	264.4	n/a	n/a	C52 H56 N8 O8 F3 S

Summenformelvorschlag

NMR (500 MHz, DMSO):

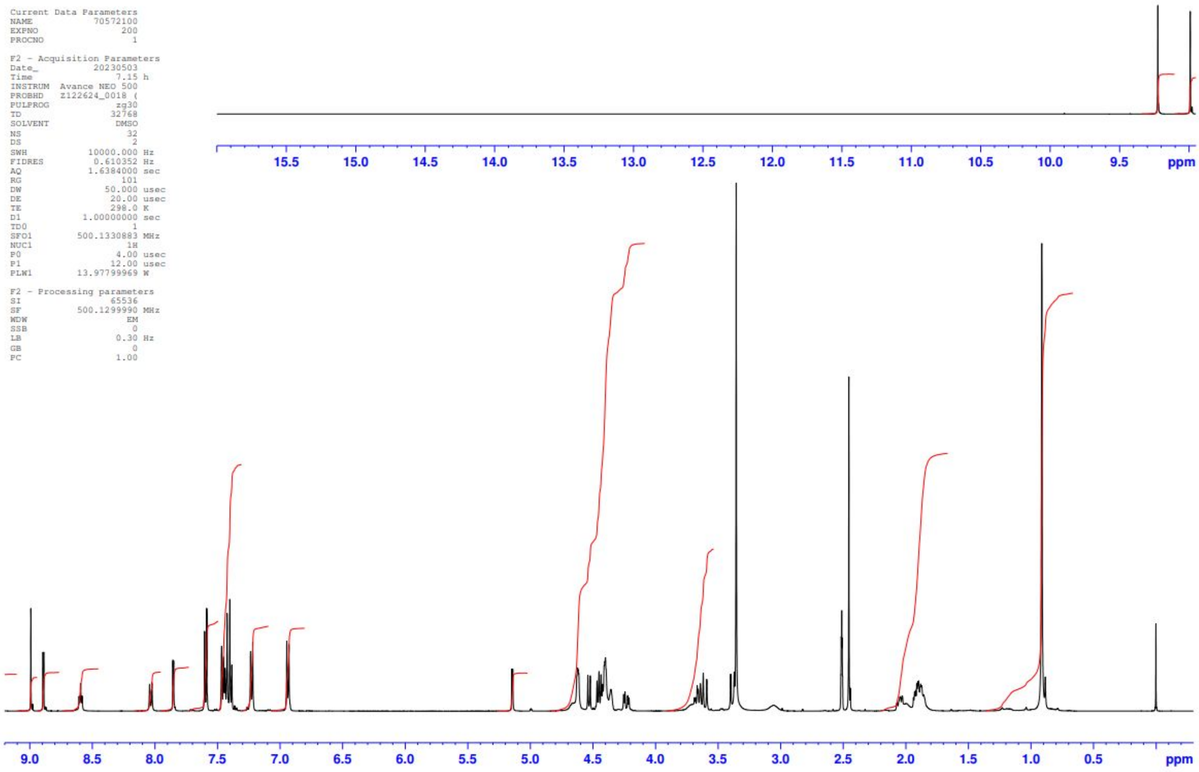

3-methyl-N-[(5-{2-[(4-{1-[4-(trifluoromethoxy)benzoyl]piperidin-4-yl}pyrido[3,2-d]pyrimidin-7-yl)oxy]ethoxy}pyridin-2-yl)acetyl]-L-valyl-(4R)-4-hydroxy-N-[4-(4-methyl-1,3-thiazol-5-yl)benzyl]-L-prolinamide (**6**):

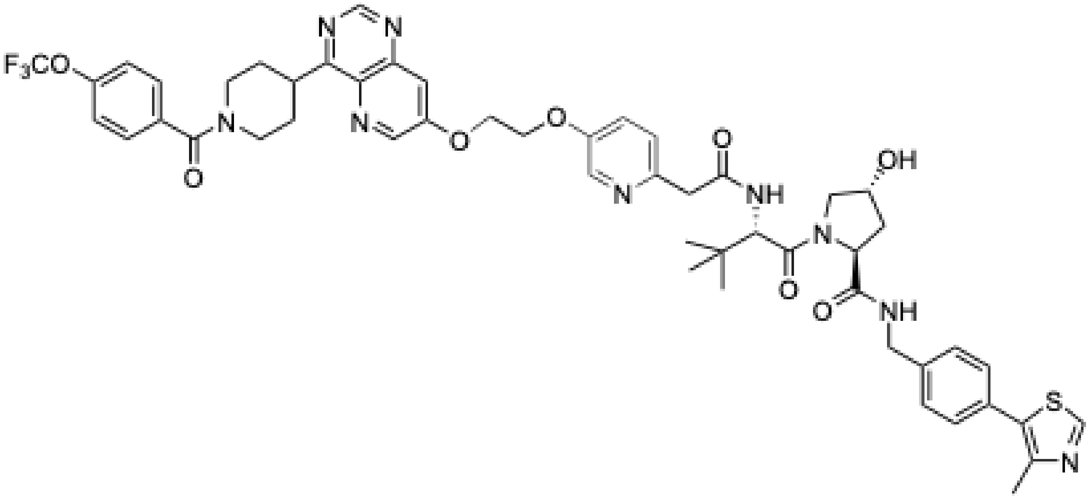

LC-MS (Method 2): R_t = 4.34 min; MS (ESIpos): m/z = 1010 $[M+H]^+$

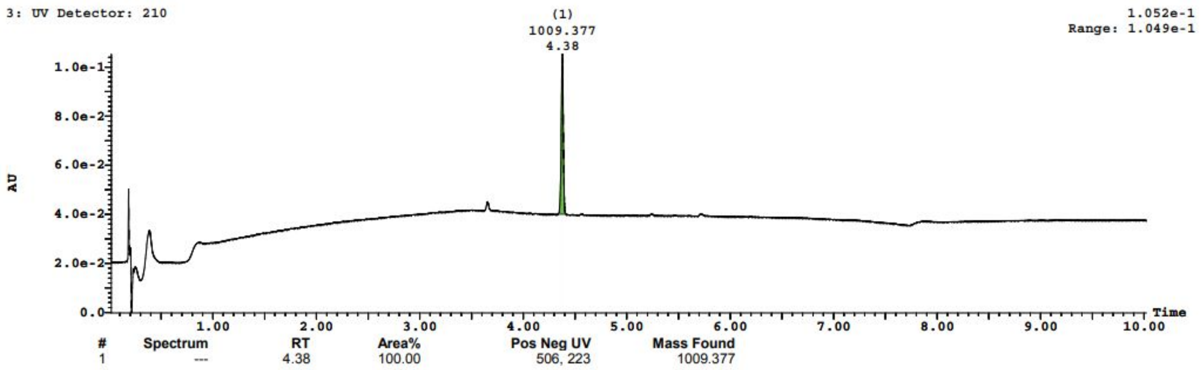

HRMS (ESI) m/z: calcd for $C_{51}H_{55}N_9O_8F_3S$ $[M+H]^+$, 1010.3846; found, 1010.3849

Elemental Composition Report **Page 1**

Single Mass Analysis
Tolerance = 2.0 PPM / DBE: min = -1.0, max = 30.0
Element prediction: Off
Number of isotope peaks used for i-FIT = 3

Monoisotopic Mass, Even Electron Ions
390 formula(e) evaluated with 1 results within limits (up to 50 closest results for each mass)
Elements Used:
C: 45-55 H: 10-100 N: 0-12 O: 0-10 F: 3-3 S: 1-1

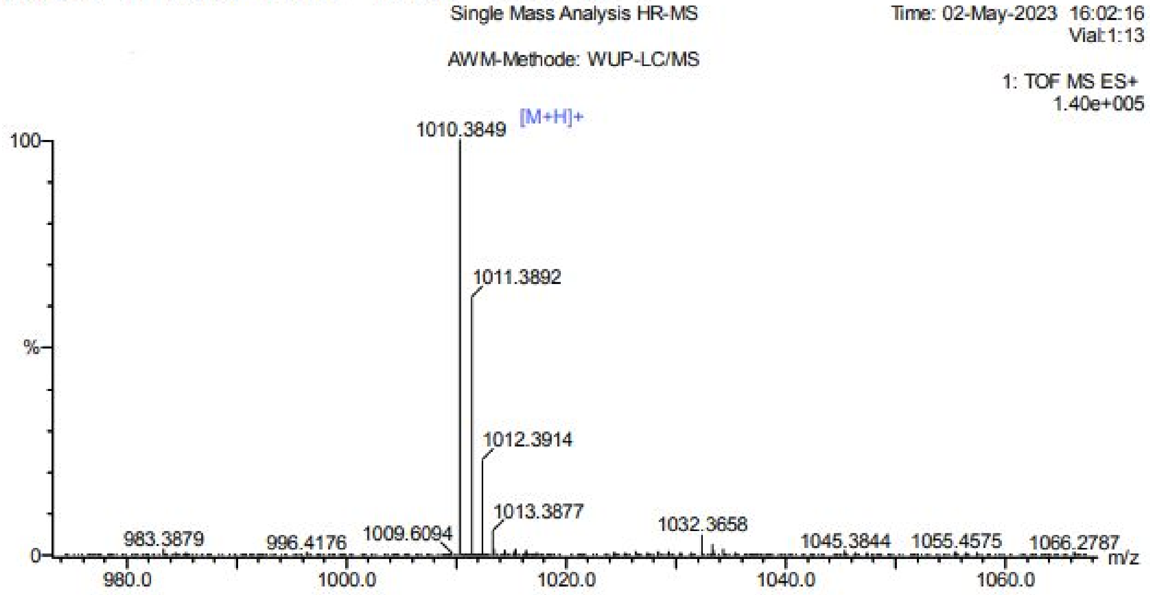

Minimum:				-1.0				
Maximum:		5.0	2.0	30.0				
Mass	Calc. Mass	mDa	PPM	DBE	i-FIT	Norm	Conf(%)	Formula
1010.3849	1010.3846	0.3	0.3	27.5	283.6	n/a	n/a	C51 H55 N9 O8 F3 S

Summenformelvorschlag

NMR (500 MHz, DMSO):

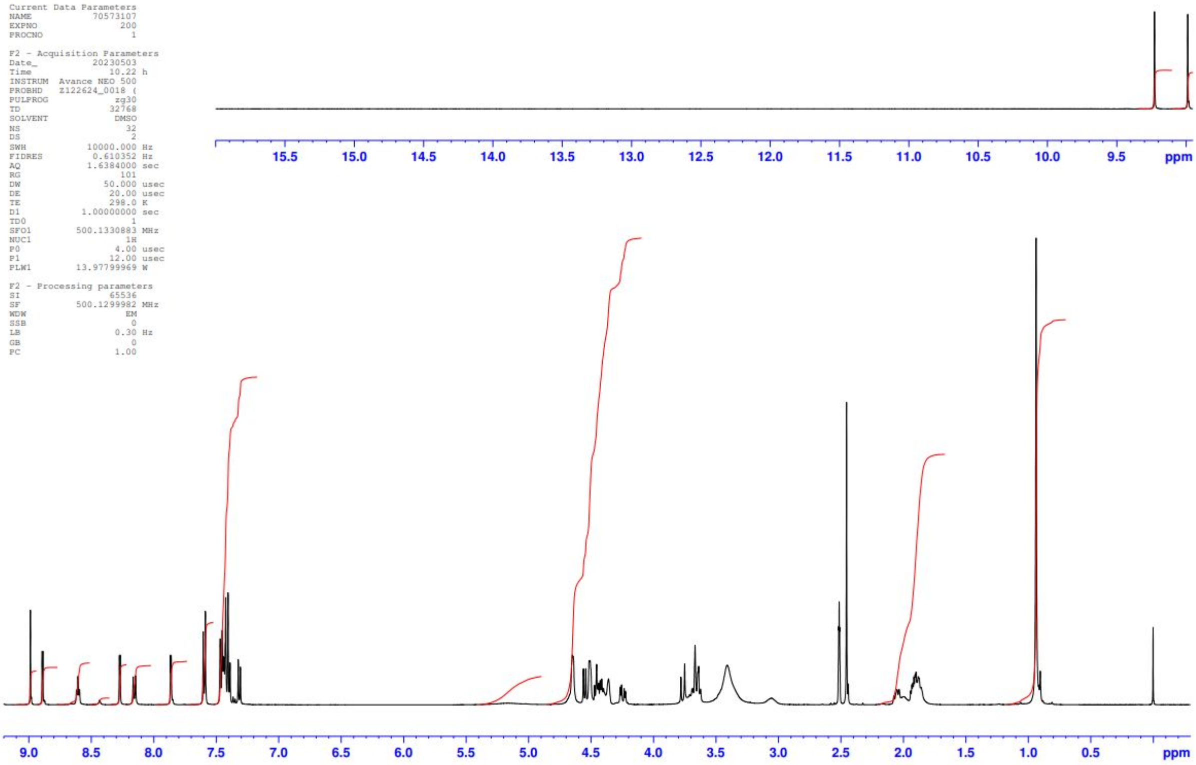

N-methyl-N-{6-[(4-{1-[4-(trifluoromethoxy)benzoyl]piperidin-4-yl}pyrido[3,2-d]pyrimidin-7-yl)oxy]hexyl}glycyl-3-methyl-L-valyl-(4R)-4-hydroxy-N-[4-(4-methyl-1,3-thiazol-5-yl)benzyl]-L-prolinamide (**7**):

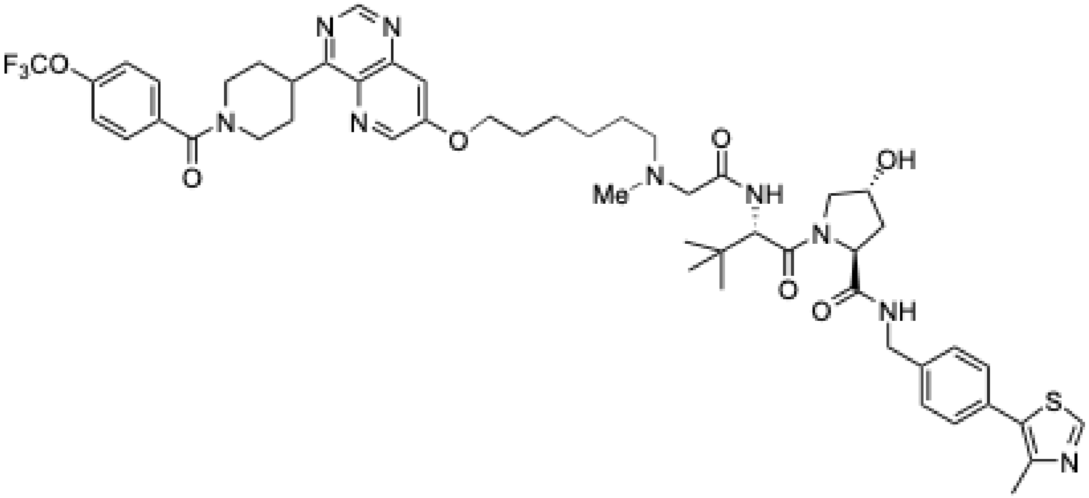

LC-MS (Method 2): R_t = 3.70 min; MS (ESIpos): m/z = 1002 $[M+H]^+$

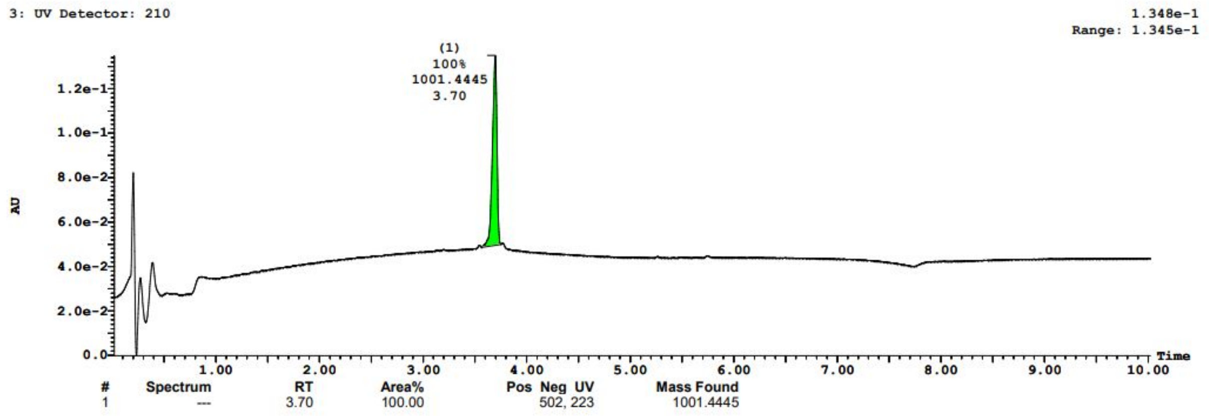

MS (ESI) m/z: calcd for $C_{51}H_{63}N_9O_7F_3S$ $[M+H]^+$, 1002.4445; found, 1002.4532

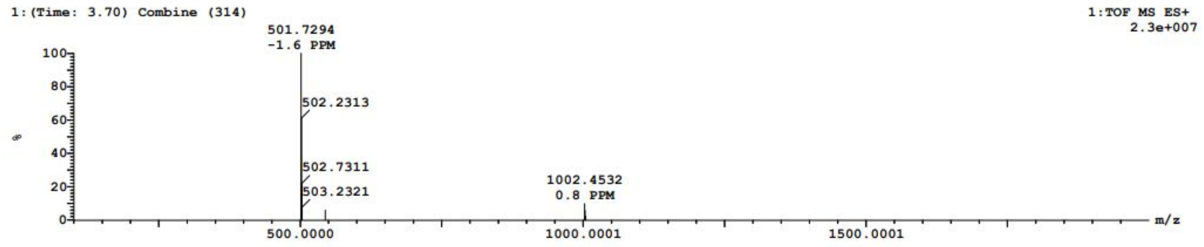

NMR (400 MHz, DMSO):

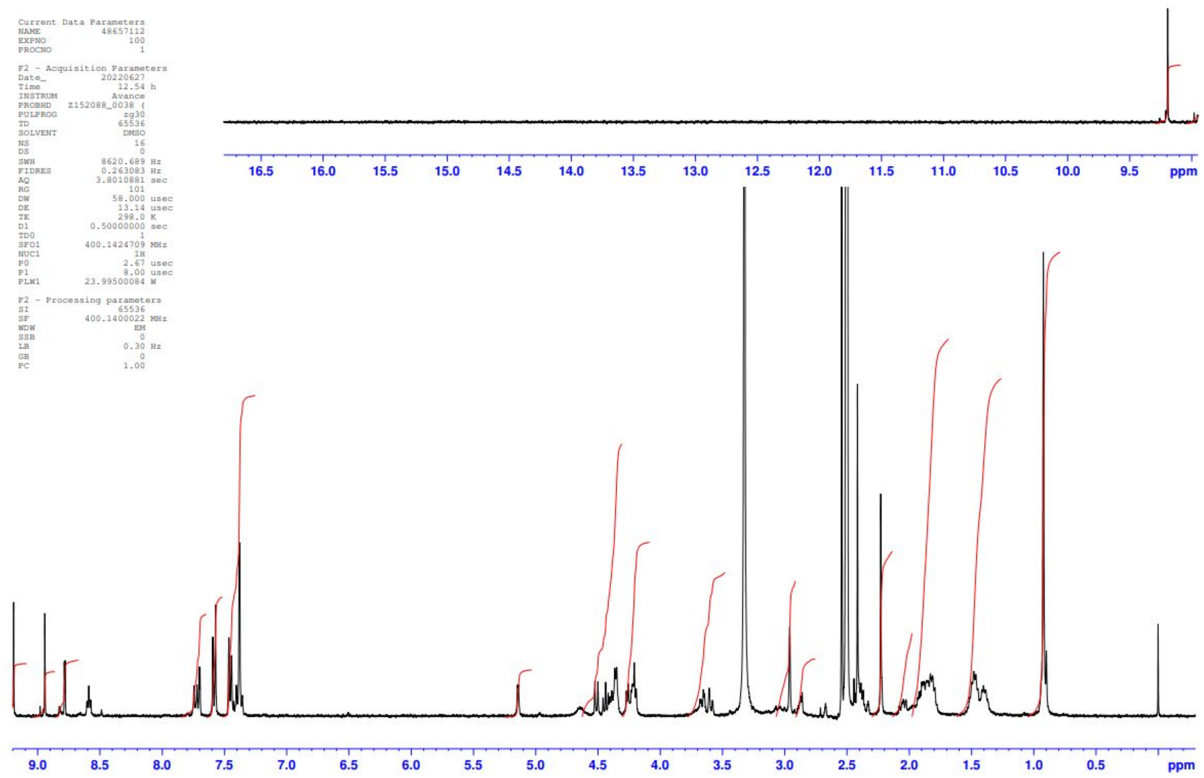

3-methyl-N-[(4-{3-[(4-{1-[4-(trifluoromethoxy)benzoyl]piperidin-4-yl}pyrido[3,2-d]pyrimidin-7-yl)oxy]propyl}piperidin-1-yl)acetyl]-L-valyl-(4R)-4-hydroxy-N-[4-(4-methyl-1,3-thiazol-5-yl)benzyl]-L-prolinamide (**8**):

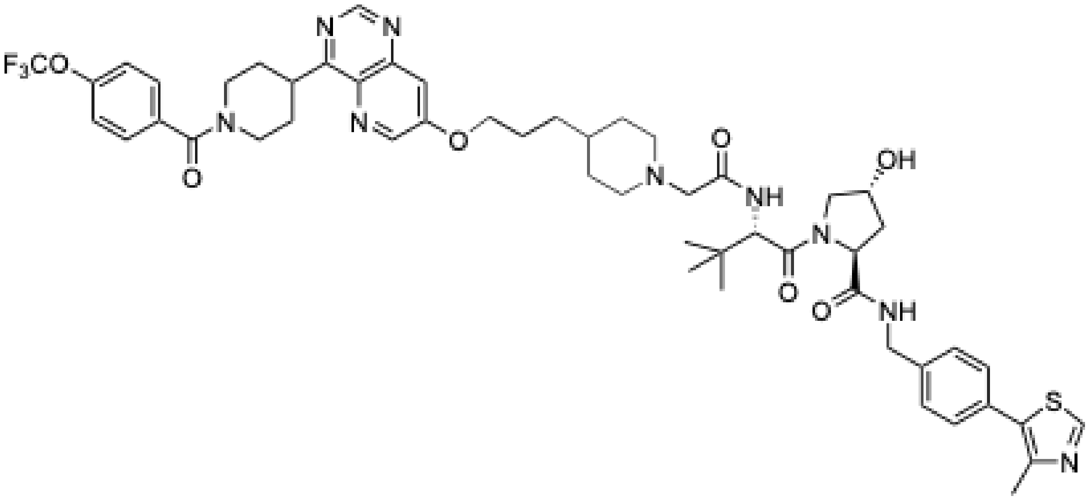

LC-MS (Method 2): R_t = 3.66 min; MS (ESIpos): m/z = 1014 $[M+H]^+$

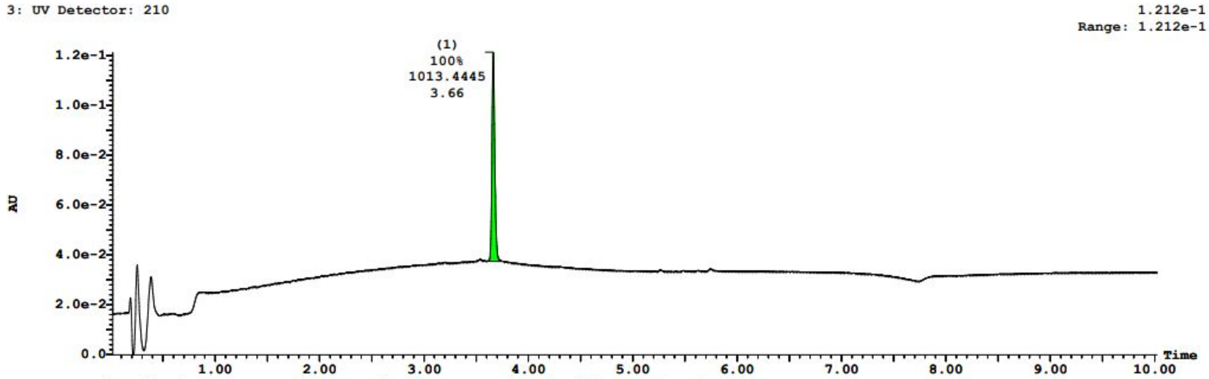

#	Spectrum	RT	Area%	Pos Neg UV	Mass Found
1	---	3.66	100.00	508, 223	1013.4445

HRMS (ESI) m/z: calcd for $C_{52}H_{63}N_9O_7F_3S$ $[M+H]^+$, 1014.4523; found, 1014.4539

Elemental Composition Report **Page 1**

Single Mass Analysis
Tolerance = 2.0 PPM / DBE: min = -1.0, max = 50.0
Element prediction: Off
Number of isotope peaks used for i-FIT = 4

Monoisotopic Mass, Even Electron Ions
482 formula(e) evaluated with 2 results within limits (up to 50 closest results for each mass)
Elements Used:
C: 40-55 H: 10-80 N: 0-10 O: 0-10 F: 1-3 S: 1-1

Single Mass Analysis HR-MS
Waters Time Of Flight (ToF);Electrospray Ionization (ESI)
AWM-Methode: WUP-LC/MS

Time: 09-Aug-2023 11:01:16
Vial:1:6

1: TOF MS ES+
8.53e+005

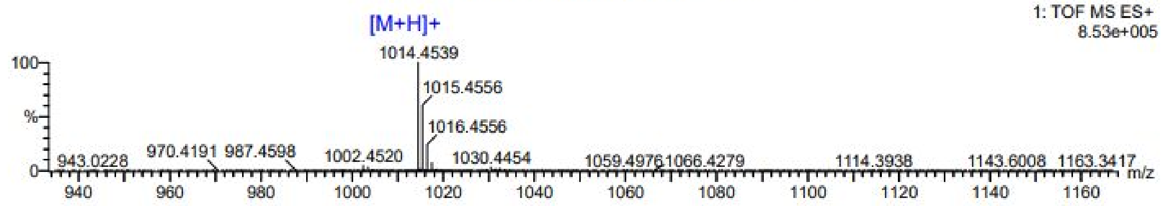

Minimum:				-1.0				
Maximum:		5.0	2.0	50.0				

Summenformelvorschlag

Mass	Calc. Mass	mDa	PPM	DBE	i-FIT	Norm	Conf(%)	Formula
1014.4539	1014.4523	1.6	1.6	24.5	661.9	0.047	95.45	C52 H63 N9 O7 F3 S
	1014.4559	-2.0	-2.0	23.5	665.0	3.090	4.55	C51 H65 N9 O10 F S

NMR (400 MHz, DMSO):

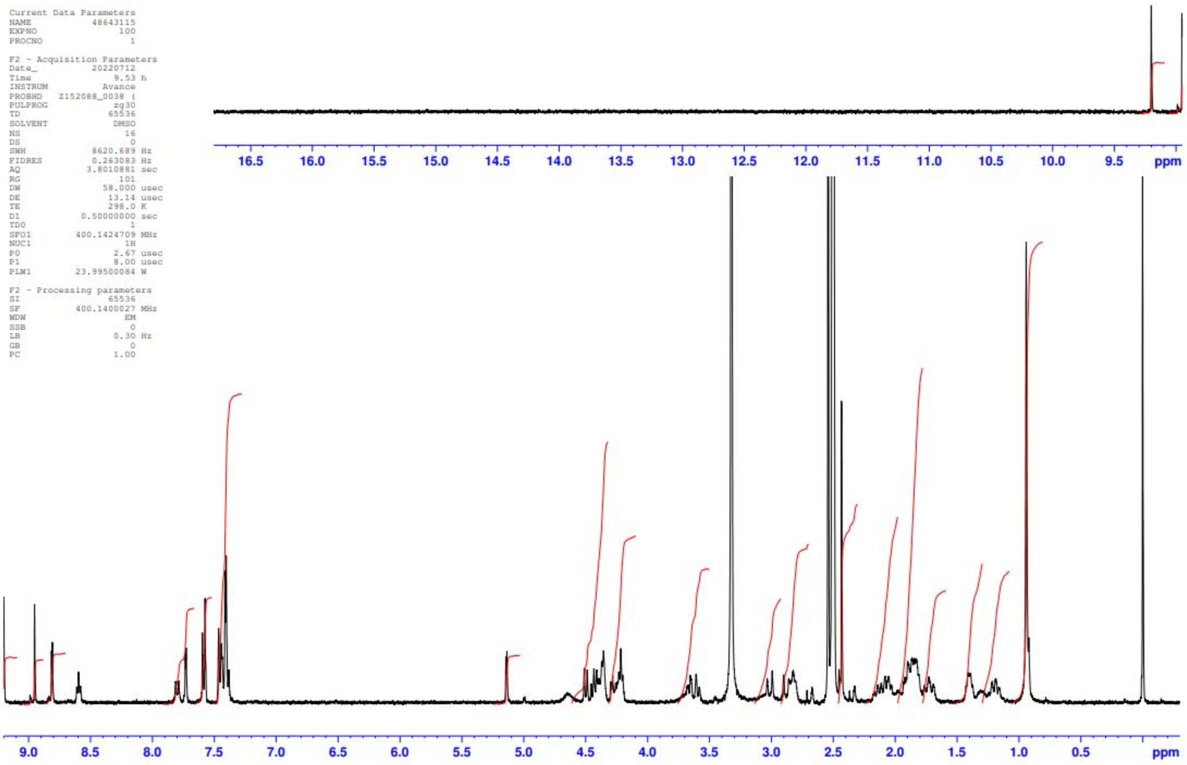

2. Physicochemical property correlations for PROTACs 1–9

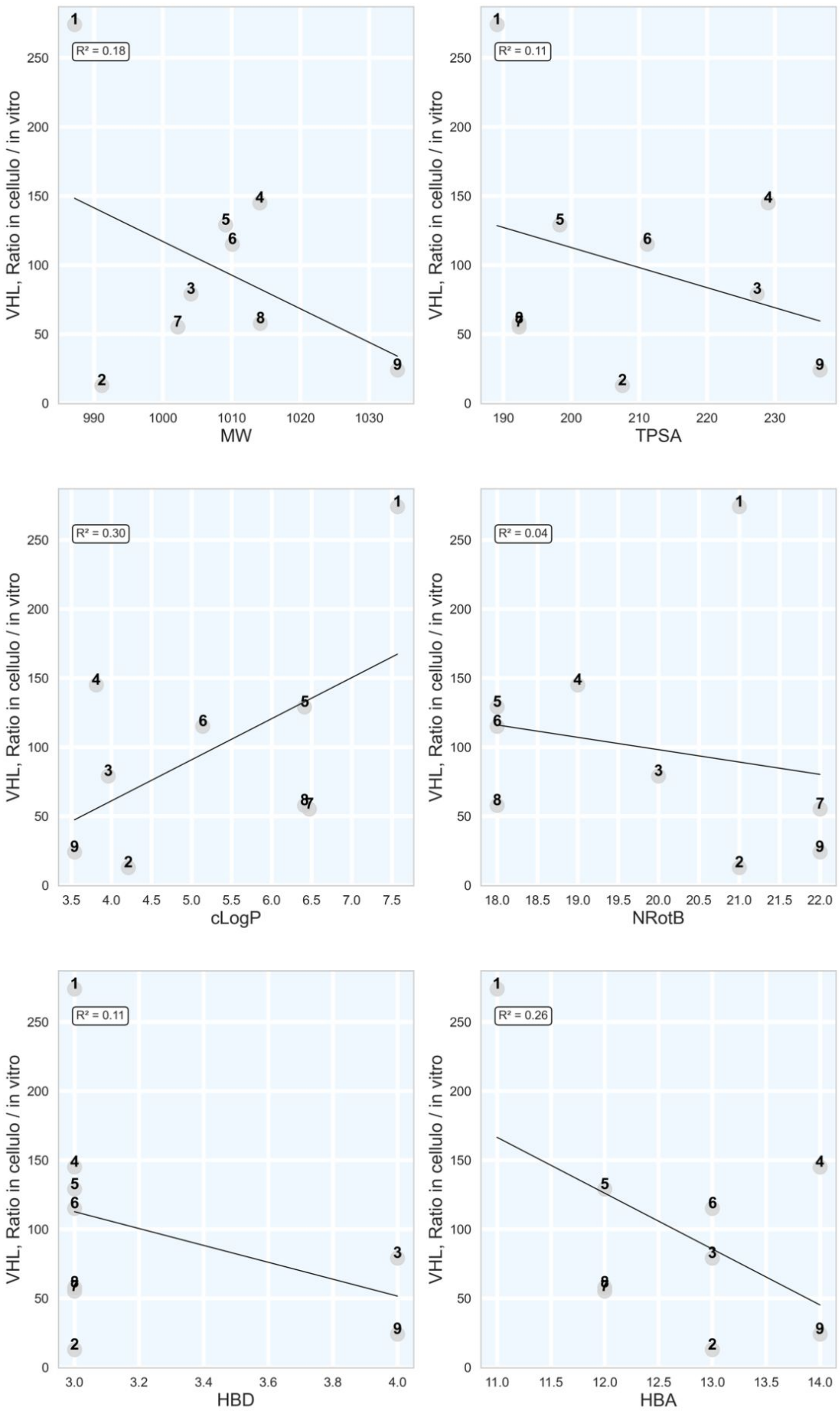

Figure S1. Correlations between the cell permeabilities of PROTACs **1**–**9**, determined as the ratio between the *in cellulo* and *in vitro* potencies for binding to VHL, and calculated descriptors.

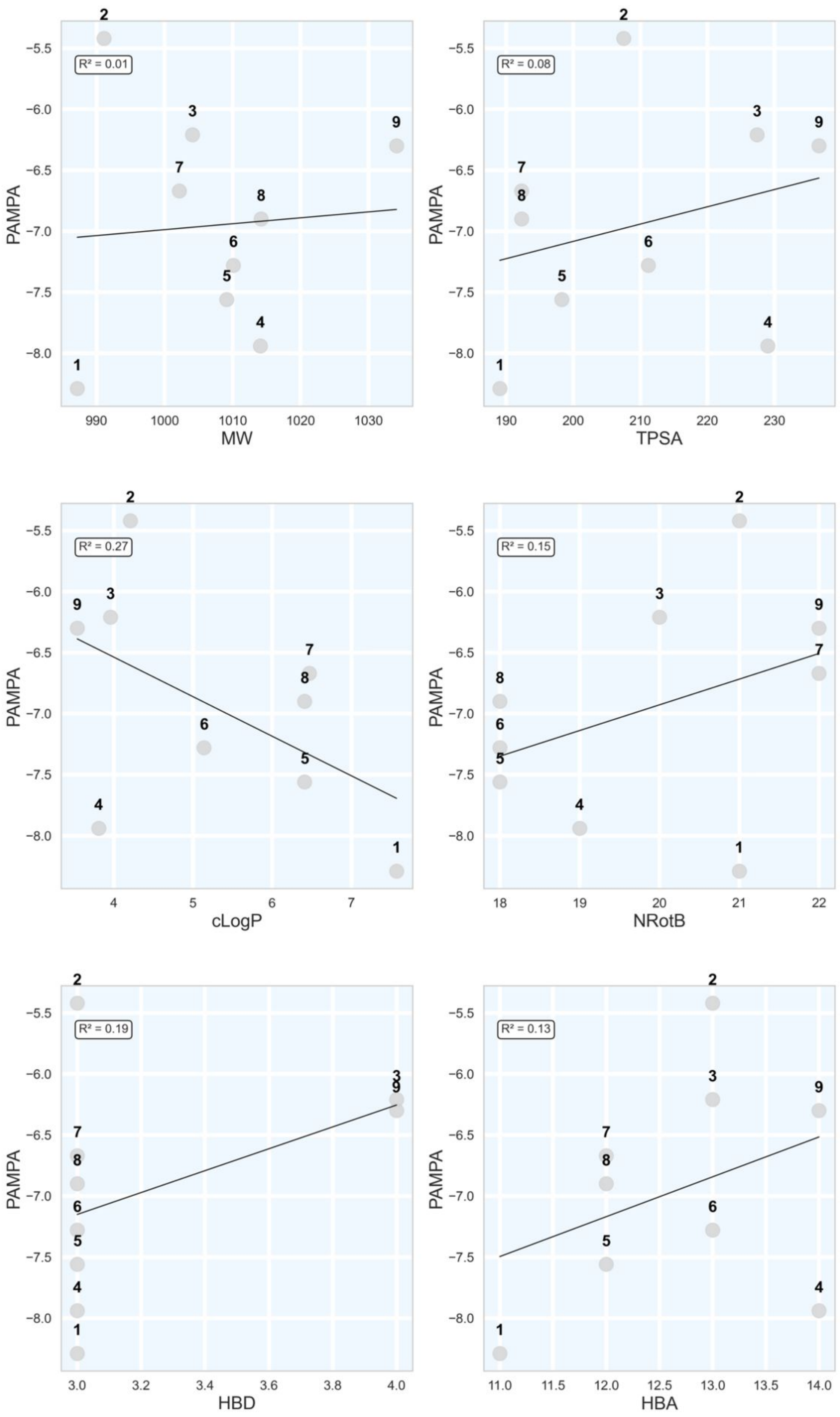

Figure S2. Correlations between the PAMPA permeabilities of PROTACs **1**–**9** and calculated descriptors.

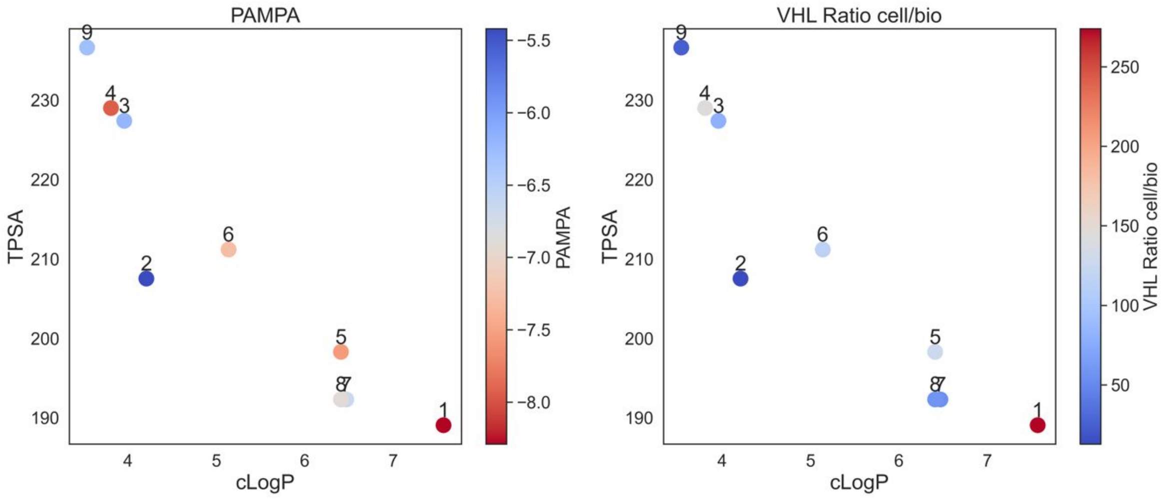

Figure S3. Graphical visualization of the PAMPA and cell permeabilities of PROTACs **1**–**9** with the combination of TPSA and cLogP. High permeabilities are in dark blue, while low permeabilities are in red. Cell permeabilities were determined as the ratio between the *in cellulo* and *in vitro* potencies for binding to VHL. Note that highly permeable PROTACs have a low ratio, while a high ratio characterizes a PROTAC with low permeability.

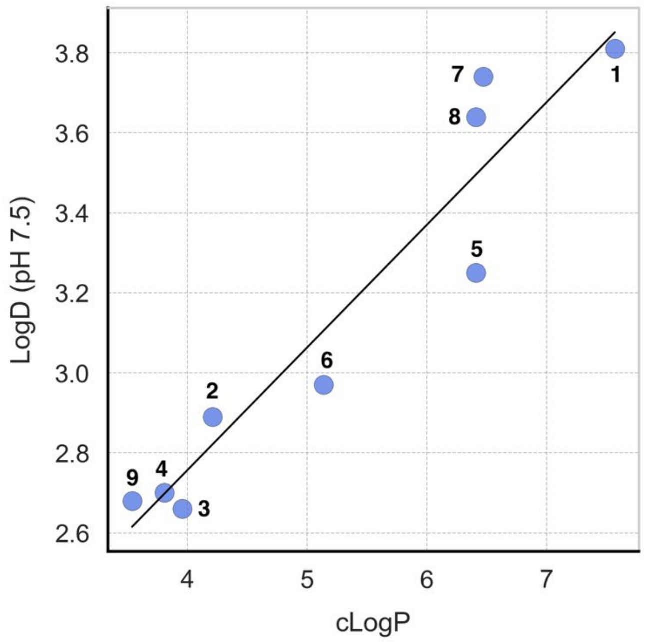

Figure S4. Correlation between chromatographic LogD and cLogP for PROTACs **1**–**9**.

3. ^{1}H-NMR assignments for PROTACs 1, 2, 3, 6 and 7

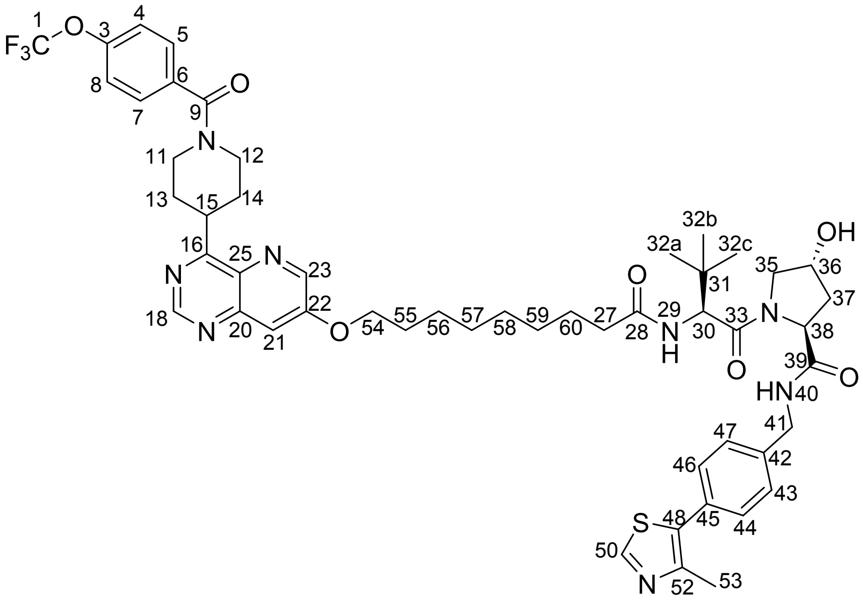

Structure and numbering of non-hydrogen atoms of PROTAC **1**

Table S1. ^{1}H- and ^{13}C-NMR assignments for PROTAC **1** at -25 ˚C.

Atom	^{13}C (ppm)	^{1}H (ppm)	Atom	^{13}C (ppm)	^{1}H (ppm)
	**POI**		33	171.7	-
1	n.a.	-	35	56.6	4.08; 3.61
3	149.6	-	36	69.9	4.50
4-8	121.0	7.28	36-OH	-	n.a.
5-7	128.5	7.52	37	35.9	2.41; 2.15
6	134.7	-	38	58.5	4.67
9	169.1	-	39	170.8	-
11-14	48.0	3.93; 3.30	40-NH	-	7.48
	42.7	4.89; 3.06	41	42.9	4.58; 4.30
	37.2	2.08; 1.98	42	137.8	-
	30.8	2.07; 1.89	43-47	127.9	7.35
15	37.1	4.41	44-46	129.3	7.34
16	172.9	-	45	130.6	-
18	155.6	9.18	48	131.6	-
20	147.9	-	50	150.5	8.72
21	112.1	7.47	52	148.0	-
22	158.1	-	53	16.0	2.51
23	146.3	8.70		**Linker**	
25	133.9	-	54	68.8	4.11
	**VHL**		55	28.4	1.87
27	36.4	2.17	56	25.7	1.47
28	173.9	-	57	28.9	1.30
29-NH	-	6.24	58	29.1	1.30
30	57.1	4.51	59	29.3	1.30
31	35.0	-	60	25.4	1.57
32a,b,c	26.2	0.92			

n.a. Signal not assigned in the spectrum

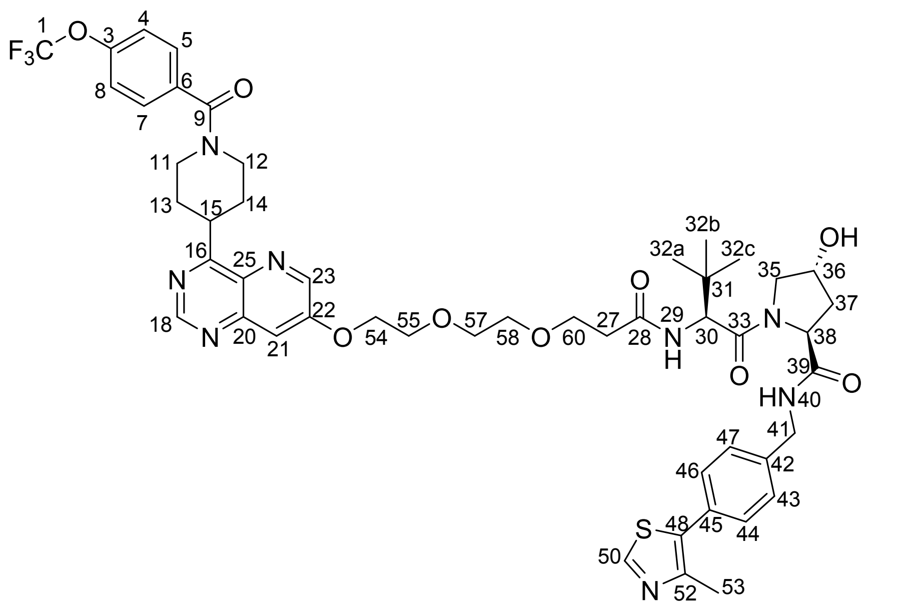

Structure and numbering of non-hydrogen atoms of PROTAC **2**

Table S2. ^{1}H- and ^{13}C-NMR assignments for PROTAC **2** at -25 ˚C.

Atom	^{13}C (ppm)	^{1}H (ppm)	Atom	^{13}C (ppm)	^{1}H (ppm)
	**POI**		32a,b,c	26.0	1.01
1	n.a.	-	33	170.3	-
3	149.5	-	35	56.8	4.14; 3.72
4-8	121.0	7.27	36	69.9	4.51
5-7	128.5	7.52	36-OH	-	n.a.
6	134.2	-	37	36.9	2.28; 2.22
9	169.0	-	38	58.7	4.64
11-14	47.7	3.92; 3.29	39	172.0	-
	42.2	4.89; 3.04	40-NH	-	9.01
	30.6	2.05; 1.85	41	42.7	4.73; 4.31
	30.1	2.05; 1.97	42	138.5	-
15	37.1	4.39	43-47	127.9	7.39
16	173.2	-	44-46	129.1	7.28
18	155.0	9.09	45	130.1	-
20	147.5	-	48	131.4	-
21	112.6	7.75	50	150.4	8.70
22	157.9	-	52	147.9	-
23	146.3	8.73	53	15.9	2.47
25	132.8	-		**Linker**	
	**VHL**		54	68.4	4.88; 4.04
27	36.1	2.50; 2.31	55	69.0	3.69; 3.82
28	172.3	-	57	67.0	3.72; 3.66
29-NH	-	7.25	58	56.8	3.72
30	57.0	4.58	60	69.9	3.74; 3.67
31	35.0	-			

n.a. Signal not assigned in the spectrum

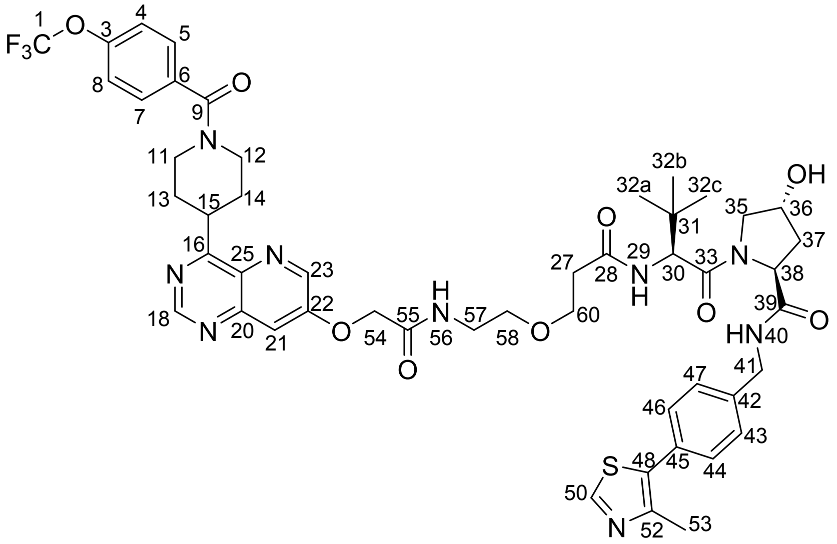

Structure and numbering of non-hydrogen atoms of PROTAC **3**

Table S3. ^{1}H- and ^{13}C-NMR assignments for PROTAC **3** at 25 ˚C.

Atom	^{13}C (ppm)	^{1}H (ppm)	Atom	^{13}C (ppm)	^{1}H (ppm)
	**POI**		35	57.1	4.19; 3.68
1	n.a.	-	36	70.1	4.55
3	149.9	-	36-OH	-	n.a.
4-8	120.9	7.72	37	36.8	2.41; 2.24
5-7	128.8	7.52	38	58.5	4.75
6	134.5	-	39	171.1	-
9	169.1	-	40-NH	-	7.57
11-14	30.7	2.07; 1.89	41	43.1	4.61; 4.30
15	37.6	4.40	42	138.1	-
16	173.8	-	43-47	128.1	7.32
18	155.7	9.18	44-46	129.4	7.29
20	147.3	-	45	129.7	-
21	113.8	7.82	48	131.3	-
22	156.8	-	50	150.2	8.65
23	146.0	8.84	52	148.2	-
25	133.7	-	53	15.9	2.43
	**VHL**			**Linker**	
27	36.6	2.41; 2.34	54	67.6	4.72; 4.59
28	172.2	-	55	166.9	-
29-NH	-	7.75	56-NH	-	7.24
30	58.2	4.61	57	39.6	3.61; 3.35
31	34.8	-	58	69.7	3.52
32a,b,c	26.6	1.02	60	66.7	3.73; 3.63
33	171.8	-			

n.a. Signal not assigned in the spectrum

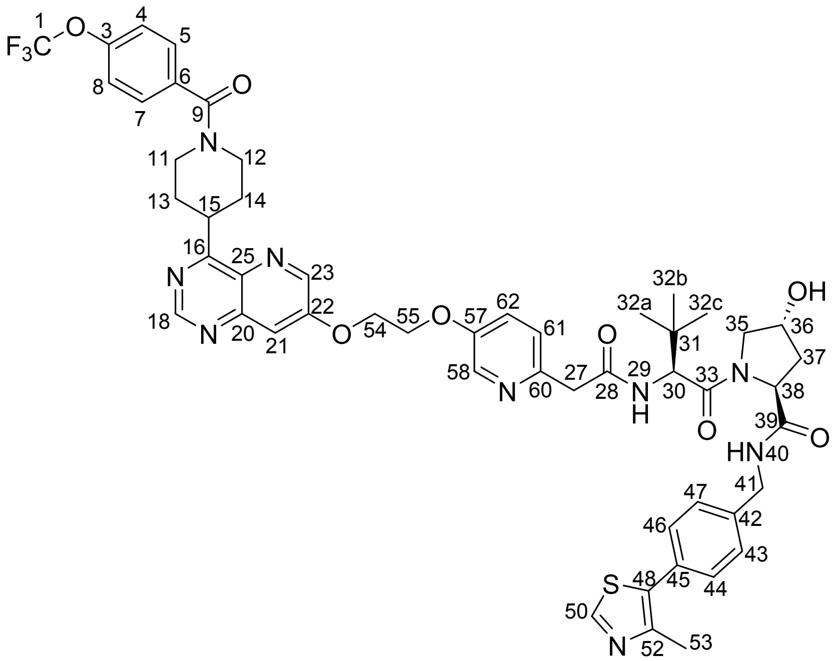

Structure and numbering of non-hydrogen atoms of PROTAC **6**

Table S4. ^{1}H- and ^{13}C-NMR assignments for PROTAC **6** at -20 ˚C.

Atom	^{13}C (ppm)	^{1}H (ppm)	Atom	^{13}C (ppm)	^{1}H (ppm)
	**POI**		33	171.9	-
1	120.6 (q)	-	35	56.7	4.11; 3.60
3	150.1	-	36	70.3	4.50
4-8	121.1	7.28	36-OH	-	n.f.
5-7	128.9	7.53	37	35.8	2.39; 2.12
6	134.9	-	38	58.3	4.71
9	169.3	-	39	170.8	-
11-14	48.0	3.94; 3.31	40-NH	-	7.79
	42.6	4.90; 3.06	41	43.3	4.52; 4.31
	30.9	2.10; 2.00	42	138.2	-
	30.8	2.10; 1.89	43-47	128.3	7.32
15	37.7	4.42	44-46	129.6	7.32
16	173.4	-	45	131.0	-
18	156.2	9.22	48	131.7	-
20	148.0	-	50	150.5	8.67
21	113.1	7.57	52	148.6	-
22	157.6	-	53	16.2	2.48
23	146.1	8.78		**Linker**	
25	133.7	-	54	67.3	4.52
	**VHL**		55	66.5	4.47
27	43.3	3.67	57	153.7	-
28	170.3	-	58	137.0	8.28
29-NH	-	8.49	60	147.9	-
30	58.3	4.41	61	124.8	7.18
31	34.7	-	62	122.8	7.26
32a,b,c	26.6	0.92			

n.f. Signal was not found in the spectrum due to exchange

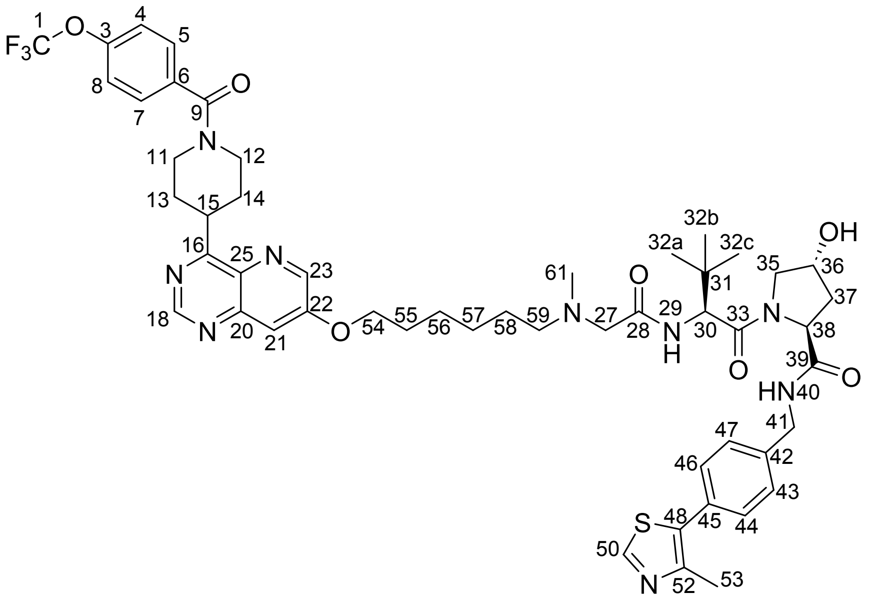

Structure and numbering of non-hydrogen atoms of PROTAC **7**

Table S5. ^{1}H- and ^{13}C-NMR assignments for PROTAC **7** at -25 ˚C.

Atom	^{13}C (ppm)	^{1}H (ppm)	Atom	^{13}C (ppm)	^{1}H (ppm)
	**POI**		33	170.8	-
1	120.3 (q)	-	35	56.9	4.11; 3.66
3	149.8	-	36	70.0	4.51
4-8	121.2	7.27	36-OH	-	n.f.
5-7	128.8	7.52	37	36.5	2.38; 2.16
6	134.6	-	38	58.5	4.73
9	169.2	-	39	171.5	-
11-14	47.9	3.92; 3.30	40-NH	-	8.29
	42.5	4.89; 3.05	41	43.0	4.57; 4.33
	30.9; 30.8	2.07; 1.98	42	138.3	-
	30.5; 30.4	2.07; 1.86	43-47	128.0	7.33
15	37.2	4.39	44-46	129.4	7.30
16	173.2	-	45	130.6	-
18	155.6	9.14	48	131.6	-
20	148.0	-	50	150.6	8.68
21	112.2	7.52	52	148.3	-
22	158.4	-	53	16.2	2.48
23	146.5	8.68	**Linker**		
25	133.0	-	54	68.6	4.22; 4.01
	**VHL**		55	28.7	1.91; 1.79
27	61.1	2.94	56	25.6	1.51
28	171.9	-	57	26.5	1.43; 1.38
29-NH	-	8.00	58	27.3	1.51; 1.43
30	56.9	4.47	59	57.7	2.44; 2.38
31	35.2	-	61	42.8	2.22
32a,b,c	26.4	0.98			

n.f. Signal was not found in the spectrum due to exchange

4. Interproton distances from NOE build-up curves for PROTACs 1, 2, 3, 6 and 7

Table S6. Interproton distances (Å) derived from NOE build-up curves in $CDCl_3$ for PROTAC **1** at -25 ˚C

No.	Proton A	Proton B	δ_A (ppm)	δ_B (ppm)	σ	R^2	Distance, r_{AB} (Å)
1	**18**	**21**	9.18	7.47	3.212×10^{-6}	0.99	4.16
2	**23**	**56**	8.70	1.47	3.710×10^{-6}	0.99	4.13
3	**23**	**55**	8.70	1.87	3.916×10^{-6}	0.99	4.09
4	**40-NH**	**37'**	7.48	2.41	2.889×10^{-5}	0.99	2.93
5	**40-NH**	**41''**	7.48	4.30	1.240×10^{-4}	0.99	2.30
6	**40-NH**	**38**	7.48	4.67	2.889×10^{-4}	0.98	2.00
7	**21**	**54**	7.47	4.11	1.845×10^{-4}	0.99	2.18
8	**21**	**55**	7.47	1.87	1.039×10^{-5}	0.98	3.48
9	**29-NH**	**30**	6.24	4.51	3.552×10^{-5}	0.97	2.84
10	**29-NH**	**60**	6.24	1.57	1.585×10^{-5}	0.99	3.24
11	**29-NH**	**35''**	6.24	3.61	7.358×10^{-6}	0.96	3.69
12	**29-NH**	**27**	6.24	2.17	1.446×10^{-4}	0.99	2.24
13	**38**	**35'**	4.67	4.08	8.245×10^{-6}	0.95	3.62
14	**38**	**37''**	4.67	2.15	1.563×10^{-4}	0.99	2.51
15	**30**	**37''**	4.51	2.15	1.248×10^{-4}	0.99	2.30
16	**40-NH**	**29-NH**	7.48	6.24	9.968×10^{-6}	0.99	3.50
17	**29-NH**	**35'**	6.24	4.08	1.416×10^{-5}	0.98	3.31
18	**38**	**37'**	4.67	2.41	7.469×10^{-5}	0.99	2.22
19	**38**	**35''**	4.67	3.61	1.151×10^{-5}	0.99	3.42
20	**35''**	**37''**	3.61	2.15	3.624×10^{-5}	0.98	2.83
21	**23**	**54**	8.70	4.11	5.831×10^{-6}	0.99	3.83
22	**54**	**56**	4.11	1.47	3.580×10^{-5}	0.99	2.83
23	**55**	**56**	1.87	1.47	3.436×10^{-5}	0.99	2.85
24	**54**	**55**	4.11	1.87	8.036×10^{-5}	0.99	2.47
25	**27**	**60**	2.17	1.57	5.186×10^{-5}	0.99	2.66
Ref.	**37'**	**37''**	2.41	2.15	5.805×10^{-4}	0.99	1.78
Ref.	**35'**	**35''**	4.08	3.61	7.656×10^{-4}	0.99	1.70
Ref.	**41'**	**41''**	4.58	4.30	4.586×10^{-4}	0.99	1.85

Table S7. Interproton distances (Å) derived from NOE build-up curves in $CDCl_3$ for PROTAC **2** at -25 ˚C that were included for the NAMFIS analysis**.**

No.	Proton A	Proton B	δ_A (ppm)	δ_B (ppm)	σ	R^2	Distance, r_{AB} (Å)
1	**30**	**35'**	4.58	4.14	3.779×10^{-4}	0.99	1.93
2	**21**	**54'**	7.75	4.88	1.787×10^{-4}	0.99	2.19
3	**54''**	**55'**	4.04	3.69	1.897×10^{-4}	0.99	2.16
4	**43, 47**	**41''**	7.39	4.31	1.131×10^{-4}	0.99	3.46
5	**40**	**43, 47**	9.01	7.39	1.122×10^{-5}	0.99	3.42
6	**21**	**54''**	7.75	4.04	2.347×10^{-4}	0.99	2.09
7	**23**	**54'**	8.73	4.88	5.176×10^{-6}	0.99	3.94
8	**36**	**35'**	4.51	4.14	1.419×10^{-4}	0.99	2.27
9	**40**	**41''**	9.01	4.31	1.281×10^{-4}	0.99	2.31

10	**18**	**41''**	9.09	4.31	9.070×10^{-6}	0.99	3.59
11	**54'**	**55'**	4.88	3.69	9.469×10^{-5}	0.99	2.43
12	**21**	**41'**	7.75	4.73	5.646×10^{-6}	0.99	3.89
13	**18**	**37'**	9.09	2.28	6.335×10^{-6}	0.99	3.81
14	**43, 47**	**54''**	7.39	4.04	7.202×10^{-6}	0.99	3.73
15	**54''**	**55''**	4.04	3.82	1.494×10^{-4}	0.99	2.25
16	**36**	**37'**	4.51	2.28	1.185×10^{-4}	0.99	2.34
17	**40**	**41'**	9.01	4.73	9.062×10^{-5}	0.99	2.45
18	**46, 44**	**54'**	7.28	4.88	6.173×10^{-6}	0.99	3.83
19	**21**	**43, 47**	7.75	7.39	9.552×10^{-6}	0.99	3.56
20	**23**	**54''**	8.73	4.04	8.392×10^{-6}	0.99	3.64
21	**21**	**41''**	7.75	4.31	6.827×10^{-6}	0.98	3.77
22	**54'**	**55''**	4.88	3.82	6.832×10^{-5}	0.98	2.57
23	**40**	**37'**	9.01	2.28	3.998×10^{-5}	0.98	2.8
24	**43, 47**	**55'**	7.39	3.69	1.654×10^{-6}	0.98	4.77
25	**21**	**55''**	7.75	3.82	2.330×10^{-5}	0.97	3.07
26	**43, 47**	**36**	7.39	4.51	9.043×10^{-7}	0.97	5.27
27	**40**	**36**	9.01	4.51	6.064×10^{-6}	0.97	3.84
28	**18**	**41'**	9.09	4.73	5.752×10^{-6}	0.97	3.87
29	**21**	**35'**	7.75	4.14	2.900×10^{-6}	0.97	4.34
30	**35'**	**37'**	4.14	2.28	2.758×10^{-5}	0.97	2.98
31	**40**	**54'**	9.01	4.88	7.004×10^{-6}	0.97	3.75
32	**43, 47**	**37'**	7.39	2.28	2.395×10^{-6}	0.97	4.48
33	**21**	**55'**	7.75	3.69	2.894×10^{-5}	0.97	2.96
34	**40**	**35'**	9.01	4.14	6.364×10^{-6}	0.96	3.81
35	**21**	**30**	7.75	4.58	2.243×10^{-6}	0.96	4.53
36	**21**	**38**	7.75	4.64	3.421×10^{-5}	0.99	2.88
37	**40**	**38**	9.01	4.64	2.937×10^{-4}	0.99	2.01
38	**18**	**43, 47**	9.09	7.39	2.612×10^{-6}	0.99	4.42
39	**38**	**35'**	4.64	4.14	2.060×10^{-5}	0.99	3.13
40	**23**	**55''**	8.73	3.82	4.701×10^{-6}	0.99	4.01
41	**23**	**55'**	8.73	3.69	2.922×10^{-6}	0.98	4.34
42	**18**	**35'**	9.09	4.14	7.577×10^{-7}	0.98	5.43
43	**18**	**36**	9.09	4.51	2.452×10^{-6}	0.97	4.47
Ref.	**41'**	**41''**	4.73	4.31	5.080×10^{-4}	0.99	1.84
Ref.	**54'**	**54''**	4.88	4.04	4.607×10^{-4}	0.99	1.87
Ref.	**55'**	**55''**	3.69	3.82	6.118×10^{-4}	0.99	1.78

Table S8. Interproton distances (Å) derived from NOE build-up curves in $CDCl_3$ for PROTAC **3** at 25 ˚C that were included for the NAMFIS analysis.

No.	Proton A	Proton B	δ_A (ppm)	δ_B (ppm)	σ	R^2	Distance, r_{AB} (Å)
1	**18**	**27''**	9.18	2.34	3.600×10^{-7}	0.95	4.42
2	**18**	**29-NH**	9.18	7.75	2.276×10^{-6}	0.91	3.25
3	**18**	**27'**	9.18	2.41	5.080-×10^{7}	0.99	4.18
4	**23**	**54''**	8.84	4.59	2.121×10^{-6}	0.97	3.29
5	**23**	**54'**	8.84	4.72	9.899×10^{-7}	0.95	3.74
6	**23**	**15**	8.84	4.40	7.937×10^{-7}	0.96	3.88

7	**23**	**21**	8.84	7.82	$5.821{\times}10^{-7}$	0.97	4.08
8	**21**	**54''**	7.82	4.59	$1.040{\times}10^{-5}$	0.99	2.53
9	**21**	**54'**	7.82	4.72	$2.437{\times}10^{-5}$	0.99	2.19
10	**29-NH**	**27''**	7.75	2.34	$5.666{\times}10^{-6}$	0.97	2.79
11	**29-NH**	**27'**	7.75	2.41	$5.969{\times}10^{-6}$	0.99	2.77
12	**29-NH**	**30**	7.75	4.61	$2.754{\times}10^{-6}$	0.98	3.15
13	**40-NH**	**38**	7.57	4.75	$1.731{\times}10^{-5}$	0.99	2.32
14	**40-NH**	**41'**	7.57	4.61	$1.131{\times}10^{-6}$	0.99	3.39
15	**40-NH**	**37''**	7.57	2.24	$1.233{\times}10^{-6}$	0.97	3.60
16	**38**	**37''**	4.75	2.24	$1.534{\times}10^{-5}$	0.99	2.37
17	**30**	**35''**	4.61	3.68	$9.672{\times}10^{-6}$	0.99	2.56
18	**30**	**35'**	4.61	4.19	$3.563{\times}10^{-5}$	0.99	2.06
19	**36**	**35'**	4.55	4.19	$5.396{\times}10^{-6}$	0.98	2.82
20	**36**	**35''**	4.55	3.68	$5.769{\times}10^{-6}$	0.99	2.79
21	**36**	**37''**	4.55	2.24	$3.760{\times}10^{-6}$	0.99	2.99
22	**35'**	**37'**	4.19	2.41	$9.911{\times}10^{-7}$	0.95	3.74
23	**35'**	**37''**	4.19	2.24	$1.654{\times}10^{-6}$	0.99	3.43
24	**35''**	**37'**	3.68	2.41	$1.522{\times}10^{-6}$	0.96	3.48
25	**35''**	**37''**	3.68	2.24	$1.431{\times}10^{-6}$	0.99	3.51
26	**60''**	**27'**	3.63	2.41	$5.270{\times}10^{-6}$	0.95	2.83
27	**57'**	**58**	3.61	3.52	$2.100{\times}10^{-5}$	0.99	2.25
28	**58**	**27''**	3.52	2.34	$9.230{\times}10^{-7}$	0.99	3.78
29	**58**	**27'**	3.52	2.41	$1.787{\times}10^{-6}$	0.99	3.39
30	**58**	**57''**	3.52	3.35	$1.331{\times}10^{-5}$	0.98	2.42
31	**18**	**30**	9.18	4.61	$6.754{\times}10^{-7}$	0.99	3.98
32	**21**	**57''**	7.82	3.35	$2.175{\times}10^{-7}$	0.97	4.81
33	**21**	**58**	7.82	3.52	$4.746{\times}10^{-7}$	0.99	4.22
34	**21**	**57'**	7.82	3.61	$4.178{\times}10^{-7}$	0.97	4.32
35	**56-NH**	**54'**	7.24	4.72	$2.801{\times}10^{-6}$	0.96	3.14
36	**56-NH**	**54''**	7.24	4.59	$3.230{\times}10^{-6}$	0.97	3.07
37	**56-NH**	**58**	7.24	3.52	$1.456{\times}10^{-6}$	0.96	3.50
38	**56-NH**	**57'**	7.24	3.61	$6.100{\times}10^{-6}$	0.98	2.76
39	**56-NH**	**57''**	7.24	3.35	$5.161{\times}10^{-6}$	0.99	2.84
Ref.	**35'**	**35''**	4.19	3.68	$8.481{\times}10^{-5}$	0.99	1.78
Ref.	**57'**	**57''**	3.61	3.35	$3.369{\times}10^{-5}$	0.99	2.08
Ref.	**37'**	**37''**	2.41	2.24	$2.329{\times}10^{-5}$	0.96	2.21
Ref.	**54'**	**54''**	4.72	4.59	$9.260{\times}10^{-5}$	0.98	1.75
Ref.	**60'**	**60''**	3.73	3.63	$7.414{\times}10^{-5}$	0.92	1.82

Table S9. Interproton distances (Å) derived from NOE build-up curves in $CDCl_3$ for PROTAC **6** at -20 ˚C that were included for the NAMFIS analysis.

No.	Proton A	Proton B	δ_A (ppm)	δ_B (ppm)	σ	R^2	Distance, r_{AB} (Å)
1	**23**	**15**	8.78	4.42	$8.773{\times}10^{6}$	0.99	4.20
2	**23**	**55**	8.78	4.47	$3.051{\times}10^{-6}$	0.97	5.01
3	**29-NH**	**38**	8.49	4.71	$1.250{\times}10^{-5}$	0.99	3.96
4	**29-NH**	**30**	8.49	4.41	$4.077{\times}10^{-5}$	0.99	3.25
5	**29-NH**	**61**	8.49	7.18	$6.525{\times}10^{-6}$	0.94	4.41
6	**29-NH**	**27**	8.49	3.67	$8.998{\times}10^{-5}$	0.99	2.85

7	**58**	**27**	8.28	3.67	4.473×10^{-6}	0.99	4.70
8	**58**	**55**	8.28	4.47	8.503×10^{-5}	0.99	2.88
9	**58**	**54**	8.28	4.52	2.288×10^{-5}	0.97	3.58
10	**40-NH**	**41''**	7.79	4.31	6.318×10^{-5}	0.98	3.02
11	**21**	**54**	7.57	4.52	2.804×10^{-4}	0.99	2.36
12	**21**	**55**	7.57	4.47	4.704×10^{-5}	0.97	3.17
13	**62**	**54**	7.26	4.52	1.214×10^{-5}	0.98	3.98
14	**61**	**27**	7.18	3.67	8.930×10^{-5}	0.99	2.85
15	**38**	**37''**	4.71	2.12	1.881×10^{-4}	0.99	2.54
16	**38**	**37'**	4.71	2.39	1.077×10^{-4}	0.99	2.77
17	**38**	**41'**	4.71	4.52	9.597×10^{-6}	0.96	4.14
18	**38**	**36**	4.71	4.50	1.815×10^{-5}	0.97	3.72
19	**38**	**35'**	4.71	4.11	1.884×10^{-5}	0.99	3.70
20	**38**	**35''**	4.71	3.60	2.320×10^{-5}	0.93	3.57
21	**36**	**35'**	4.50	4.11	1.559×10^{-4}	0.99	2.60
22	**36**	**35''**	4.50	3.60	2.077×10^{-4}	0.96	2.48
23	**36**	**37''**	4.50	2.12	1.902×10^{-4}	0.94	2.52
24	**36**	**37'**	4.50	2.39	1.680×10^{-4}	0.99	2.57
25	**30**	**35''**	4.41	3.60	2.078×10^{-4}	0.94	2.48
26	**40-NH**	**38**	7.79	4.71	3.034×10^{-4}	0.99	2.33
27	**30**	**35'**	4.41	4.11	3.436×10^{-4}	0.99	2.28
28	**35'**	**37''**	4.11	2.12	5.287×10^{-5}	0.99	3.11
29	**35''**	**37'**	3.60	2.39	8.774×10^{-5}	0.96	2.86
30	**61**	**54**	7.18	4.52	1.790×10^{-6}	0.98	5.47
31	**29-NH**	**35'**	8.49	4.11	2.395×10^{-5}	0.95	3.55
32	**29-NH**	**58**	8.49	8.28	2.377×10^{-5}	0.96	3.56
33	**40-NH**	**37'**	7.79	2.39	3.081×10^{-5}	0.97	3.41
34	**40-NH**	**37''**	7.79	2.12	4.785×10^{-5}	0.95	3.17
35	**62**	**27**	7.26	3.67	8.145×10^{-6}	0.99	4.25
36	**35'**	**37'**	4.11	2.39	4.301×10^{-5}	0.99	3.22
37	**62**	**55**	7.26	4.47	4.887×10^{-5}	0.99	3.15
Ref.	**35'**	**35''**	4.11	3.60	1.514×10^{-3}	0.93	1.78
Ref.	**37'**	**37''**	2.39	2.12	5.872×10^{-4}	0.99	2.08

Table S10. Interproton distances (Å) derived from NOE build-up curves in $CDCl_3$ for PROTAC **7** at -25 ˚C that were included for the NAMFIS analysis**.**

No.	Proton A	Proton B	δ_A (ppm)	δ_B (ppm)	σ	R^2	Distance, r_{AB} (Å)
1	**18**	**38**	9.14	4.73	5.508×10^{-6}	0.99	3.84
2	**18**	**37''**	9.14	2.16	6.281×10^{-6}	0.99	3.75
3	**23**	**54''**	8.68	4.01	3.330×10^{-6}	0.98	4.17
4	**23**	**54'**	8.68	4.22	2.467×10^{-6}	0.99	4.39
5	**40-NH**	**35'**	8.29	4.11	1.953×10^{-6}	0.97	4.56
6	**40-NH**	**38**	8.29	4.73	2.677×10^{-4}	0.99	2.01
7	**40-NH**	**54''**	8.29	4.01	1.979×10^{-6}	0.97	4.55
8	**40-NH**	**35''**	8.29	3.66	4.316×10^{-6}	0.98	4.00
9	**40-NH**	**41''**	8.29	4.33	9.779×10^{-5}	0.99	2.38
10	**40-NH**	**41'**	8.29	4.57	8.660×10^{-5}	0.99	2.42
11	**29-NH**	**57'**	8.00	1.43	3.340×10^{-5}	0.99	2.84

12	**29-NH**	**59'**	8.00	2.44	2.726×10^{-5}	0.98	2.94
13	**29-NH**	**38**	8.00	4.73	4.120×10^{-6}	0.98	4.03
14	**29-NH**	**30**	8.00	4.47	5.087×10^{-5}	0.98	2.65
15	**21**	**54''**	7.52	4.01	9.574×10^{-5}	0.99	2.38
16	**21**	**38**	7.52	4.73	7.307×10^{-6}	0.99	3.66
17	**21**	**54'**	7.52	4.22	1.106×10^{-4}	0.99	2.33
18	**21**	**55''**	7.52	1.79	5.121×10^{-6}	0.97	3.88
19	**38**	**37''**	4.73	2.16	2.284×10^{-4}	0.99	2.06
20	**36**	**35''**	4.51	3.66	1.935×10^{-4}	0.99	2.12
21	**54'**	**55''**	4.22	1.79	8.292×10^{-5}	0.99	2.44
22	**54'**	**55'**	4.22	1.91	7.015×10^{-5}	0.99	2.51
23	**54'**	**57”**	4.22	1.38	2.000×10^{-5}	0.99	3.10
24	**35'**	**37''**	4.11	2.16	2.747×10^{-5}	0.98	2.94
25	**54''**	**57”**	4.01	1.38	1.030×10^{-5}	0.98	3.46
26	**54''**	**55''**	4.01	1.79	1.045×10^{-4}	0.99	2.35
27	**54''**	**55'**	4.01	1.91	8.667×10^{-5}	0.99	2.42
28	**59'**	**57”**	2.44	1.38	5.167×10^{-5}	0.98	2.64
29	**55'**	**57”**	1.91	1.38	3.236×10^{-5}	0.99	2.86
30	**55''**	**57”**	1.79	1.38	2.537×10^{-5}	0.97	2.98
31	**36**	**37'**	4.51	2.38	1.163×10^{-4}	0.99	2.31
32	**38**	**37'**	4.73	2.38	8.727×10^{-5}	0.99	2.42
33	**30**	**35'**	4.47	4.11	3.731×10^{-4}	0.99	1.90
34	**29-NH**	**59'**	8.00	2.38	1.820×10^{-5}	0.99	3.14
35	**18**	**21**	9.14	7.52	3.134×10^{-6}	0.97	4.22
36	**59”**	**57”**	2.38	1.38	3.069×10^{-5}	0.99	2.88
37	**18**	**37'**	9.14	2.38	1.600×10^{-6}	0.98	4.72
38	**40-NH**	**21**	8.29	7.52	1.405×10^{-5}	0.99	3.28
39	**23**	**55'**	8.68	1.91	3.273×10^{-6}	0.96	4.19
40	**29-NH**	**35''**	8.00	3.66	1.054×10^{-5}	0.96	3.44
41	**27**	**59”**	2.94	2.38	7.862×10^{-5}	0.99	2.46
42	**43-47**	**54''**	7.33	4.01	3.543×10^{-6}	0.94	4.13
43	**44-46**	**54'**	7.30	4.22	1.331×10^{-6}	0.96	4.86
44	**59'**	**61**	2.44	2.22	4.065×10^{-5}	0.99	2.75
45	**59”**	**61**	2.38	2.22	4.547×10^{-5}	0.99	2.70
46	**44-46**	**54''**	7.30	4.01	3.736×10^{-6}	0.99	4.09
47	**43-47**	**54'**	7.33	4.22	2.399×10^{-6}	0.97	4.41
Ref.	**35'**	**35''**	4.11	3.66	5.530×10^{-4}	0.99	1.78
Ref.	**54'**	**54''**	4.22	4.01	5.477×10^{-4}	0.99	1.78

5. Monte Carlo Molecular Mechanics (MCMM) conformational searches for PROTACs 2, 3, 6 and 7

Table S11. Results of the Monte Carlo conformational searches

			Number of conformations	
**PROTAC #**	**Solvent**	**Force Field**	**Total[a]**	**Final ensemble[b]**
**2**	**$CHCl_3$**	OPLS2005	1205	507
		OPLS4	980	
		AMBER*	1332	
		MMFF	1707	
	**H_2O**	OPLS2005	2197	
		OPLS4	673	
		AMBER*	1633	
		MMFF	1046	
**3**	**$CHCl_3$**	OPLS	464	226
		OPLS-2005	992	
		OPLS4	1384	
		AMBER*	781	
		MMFF	1711	
	**H_2O**	OPLS	1799	
		OPLS-2005	1809	
		OPLS4	599	
		AMBER*	1390	
		MMFF	1221	
**6**	**$CHCl_3$**	OPLS	261	
		OPLS-2005	817	
		OPLS4	550	
		AMBER*	1205	
		MMFF	1100	
	**H_2O**	OPLS	230	
		OPLS-2005	510	
		OPLS4	949	
		AMBER*	483	
		MMFF	878	181
**7**	**$CHCl_3$**	OPLS	647	98
		OPLS-2005	1992	
		OPLS4	983	
		AMBER*	3082	
		MMFF	1475	
	**H_2O**	OPLS	859	

OPLS-2005	1618
OPLS4	1447
AMBER*	1244
MMFF	1068

[a]Total number of unique conformations found. The global minimum was found for all investigated compounds at least 5 times. [b]Conformations obtained after redundant conformation elimination with the root-mean-square deviation cutoff 3.0 Å for heavy atoms. To these the crystal structures (Table S11) were added to yield the final ensembles used as input in NAMFIS.

6. Conformational ensembles derived by NAMFIS-analysis for PROTACs 2, 3, 6 and 7

Table S12. Population of the conformations in the ensemble of PROTAC **2** in $CDCl_3$ at -25 ˚C.

Conf. No.[a]	%	Conf. No.[a]	%
**1**	21	**8**	6
**2**	16	**9**	3
**3**	11	**10**	3
**4**	11	**11**	2
**5**	9	**12**	2
**6**	8	**13**	2
**7**	6		

Table S13. Population of the conformations in the ensemble of PROTAC **3** in $CDCl_3$ at 25 ˚C.

Conf. No.[a]	%	Conf. No.[a]	%
**1**	24	**7**	5
**2**	16	**8**	4
**3**	15	**9**	4
**4**	13	**10**	3
**5**	6	**11**	3
**6**	5	**12**	2

Table S14. Population of the conformations in the ensemble of PROTAC **6** in $CDCl_3$ at -20 ˚C.

Conf. No.[a]	%	Conf. No.[a]	%
**1**	45	**4**	9
**2**	18	**5**	8
**3**	17	**6**	3

Table S15. Population of the conformations in the ensemble of PROTAC **7** in $CDCl_3$ at -25 ˚C.

Conf. No.[a]	%	Conf. No.[a]	%
**1**	23	**8**	5
**2**	14	**9**	3
**3**	14	**10**	2
**4**	12	**11**	2
**5**	7	**12**	2
**6**	7	**13**	2
**7**	5	**14**	2

Figure S5. Conformations adopted by POTAC **2** in $CDCl_3$ at -25 °C. Parts of the molecule which were not sampled by NAMFIS are faded out, non-polar hydrogens are omitted for clarity.

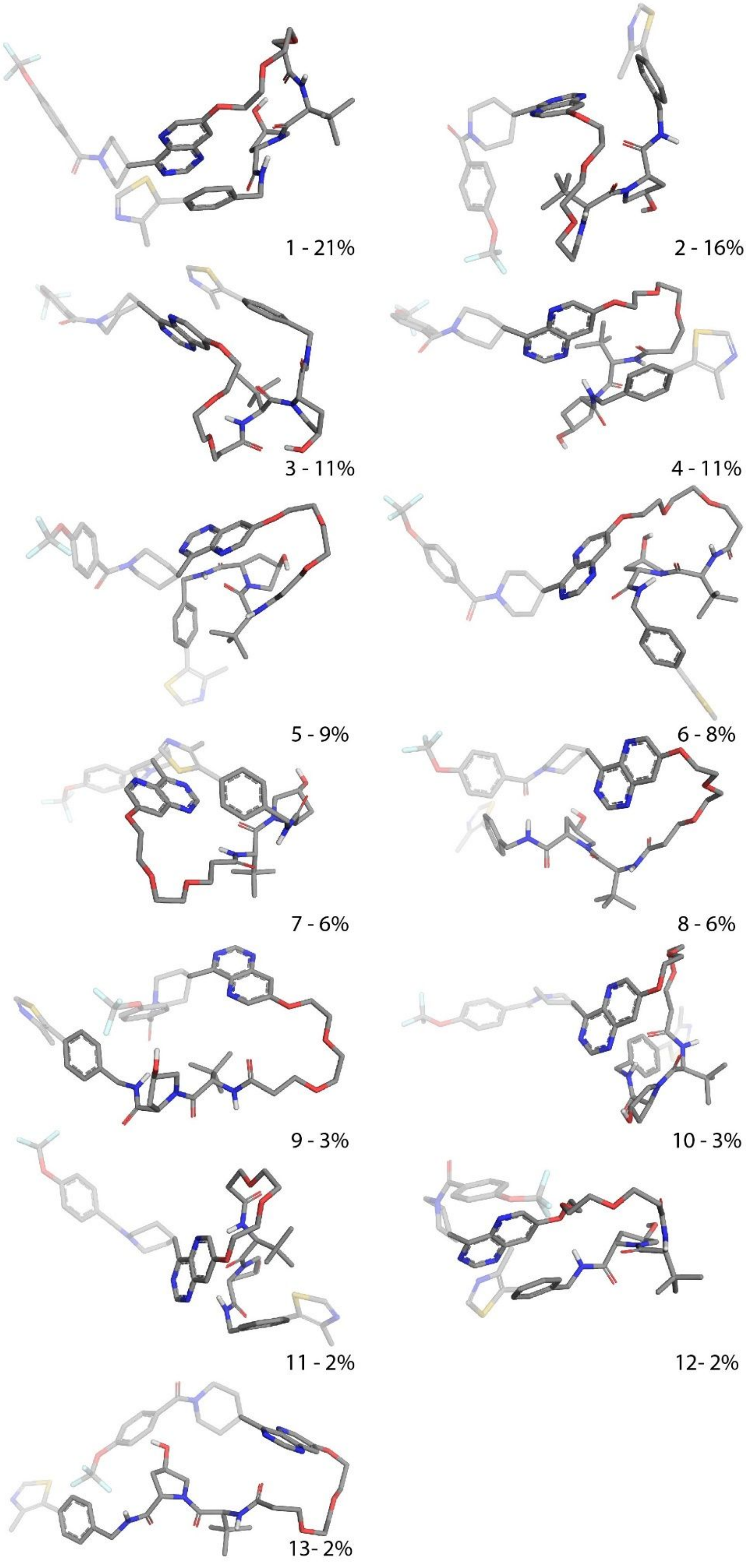

Figure S6. Conformations adopted by POTAC **3** in $CDCl_3$ at 25 °C. Parts of the molecule which were not sampled by NAMFIS are faded out, non-polar hydrogens are omitted for clarity.

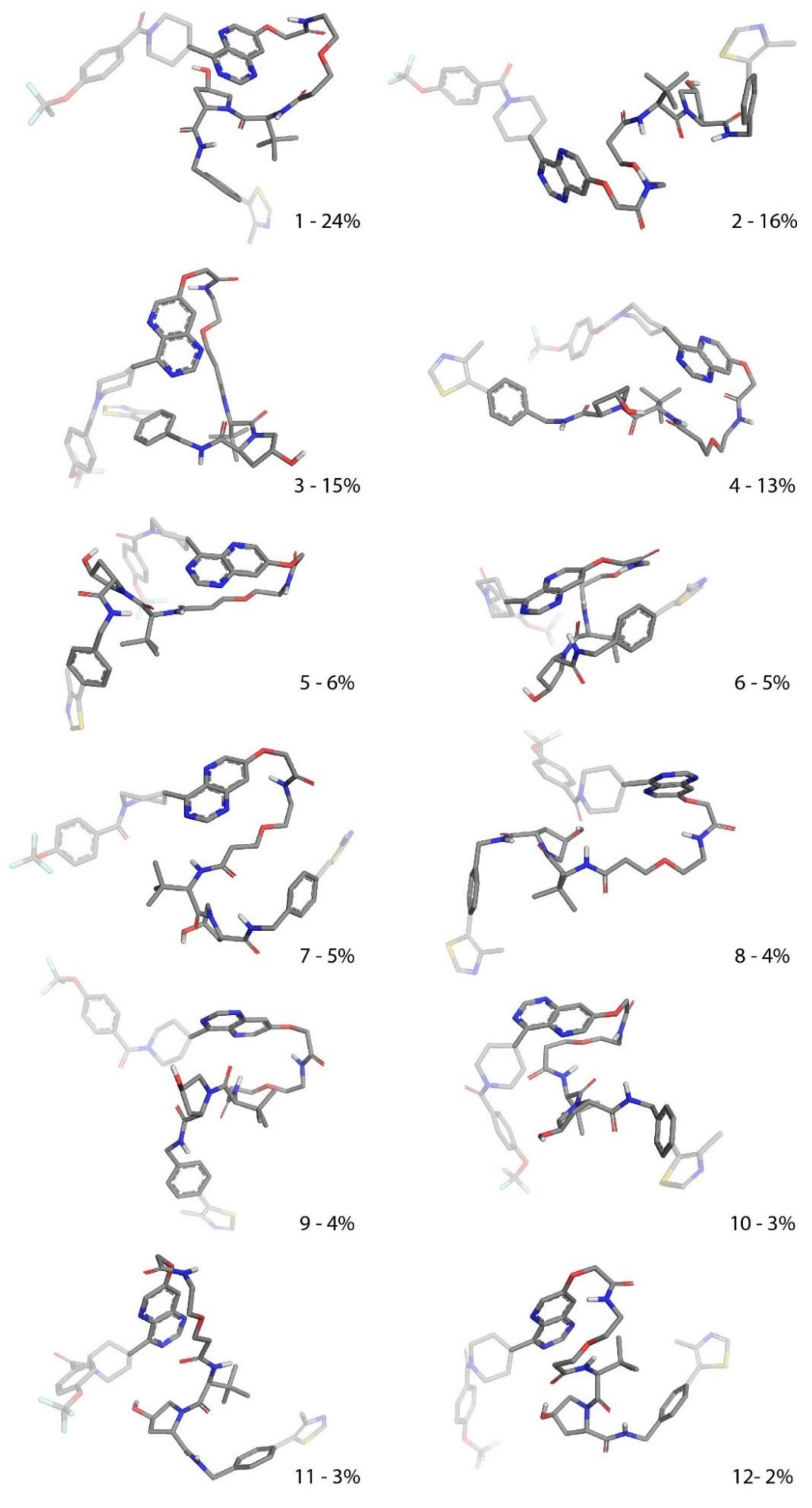

Figure S7. Conformations adopted by POTAC **6** in $CDCl_3$ at -20 °C. Parts of the molecule which were not sampled by NAMFIS are faded out, non-polar hydrogens are omitted for clarity.

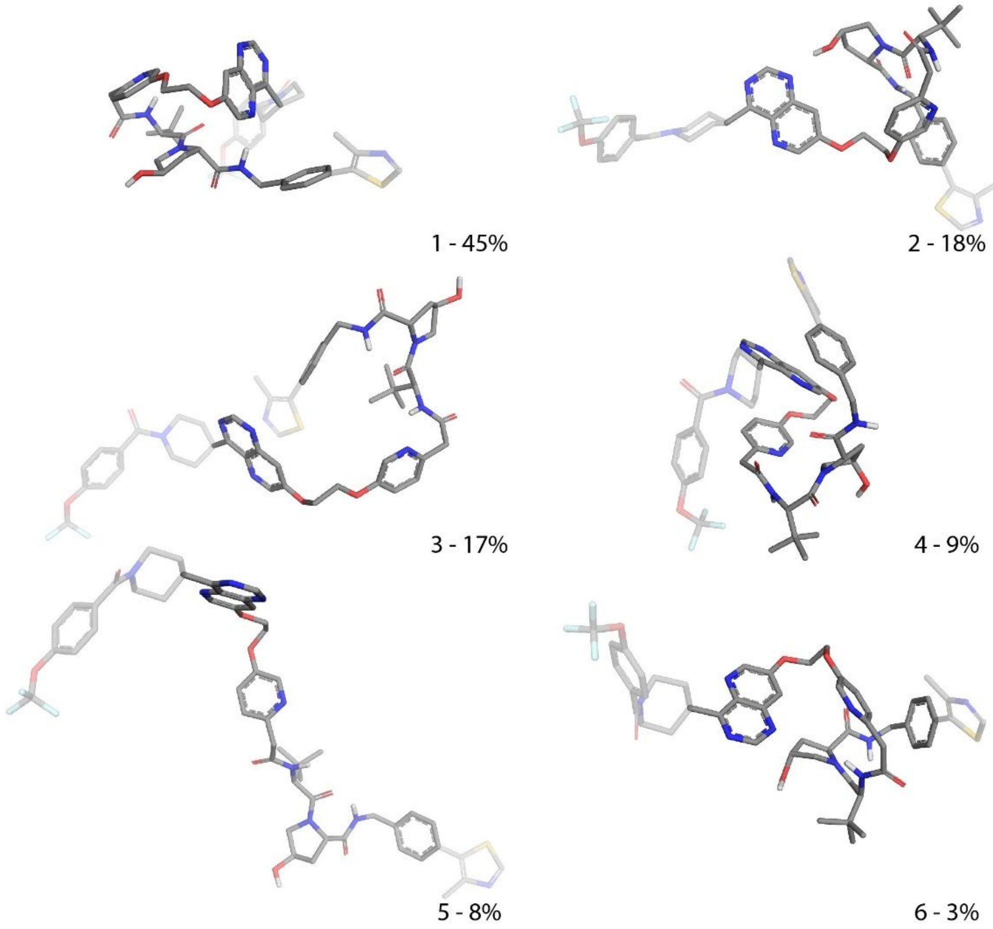

Figure S8. Conformations adopted by POTAC **7** in $CDCl_3$ at -25 °C. Parts of the molecule which were not sampled by NAMFIS are faded out, non-polar hydrogens are omitted for clarity.

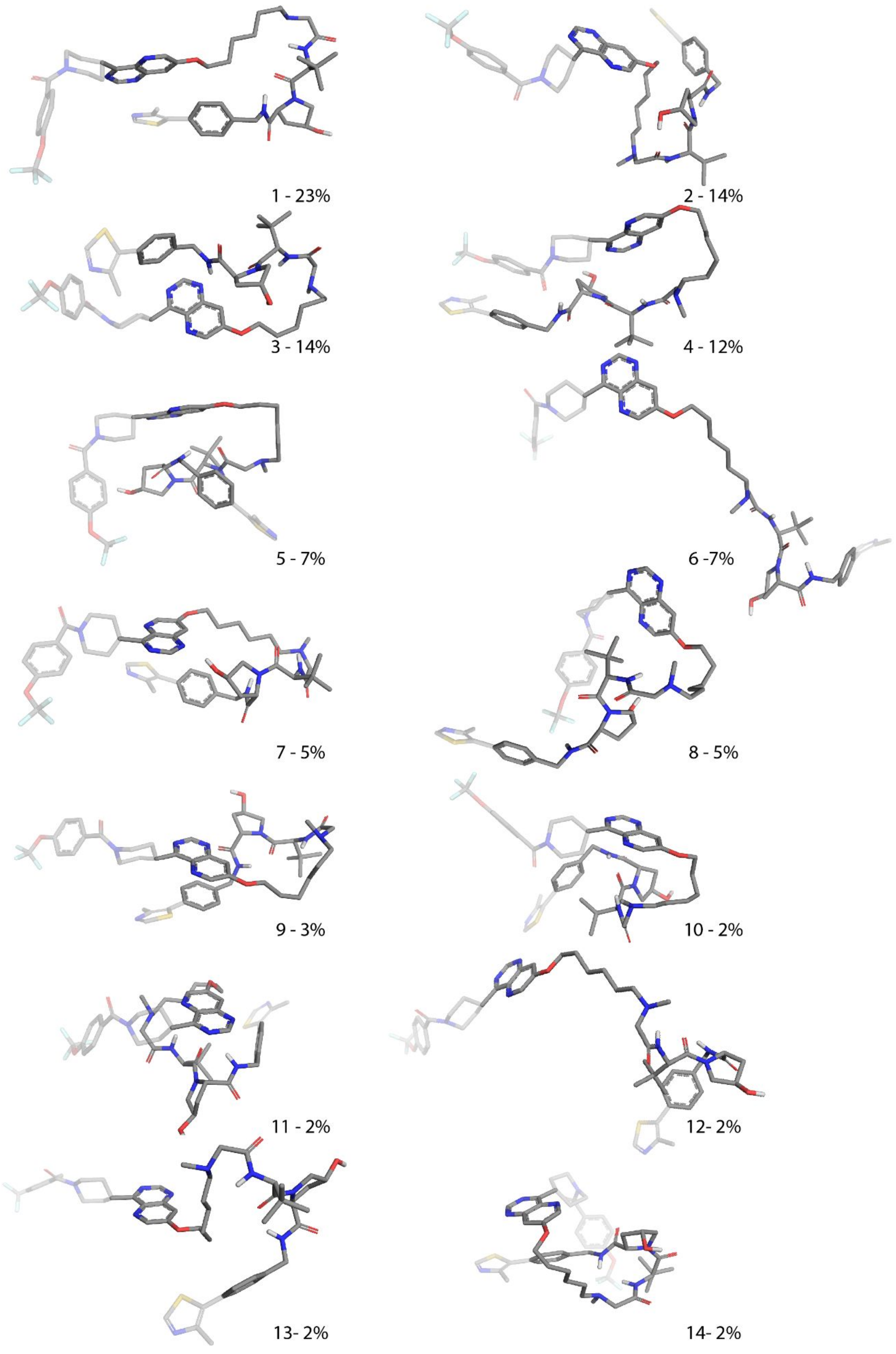

Table S16. Experimentally determined and back-calculated (NAMFIS) interproton distances (Å) of PROTACs **2** (-25 ˚C) and **3** (25 ˚C).

PROTAC **2** (-25 °C)		PROTAC **3** (25 °C)	
Exp.	Calc.	Exp.	Calc.
1.93	2.40	4.42	4.37
2.19	2.40	3.25	3.61
2.16	2.60	4.18	4.70
3.46	2.78	3.29	3.39
3.42	3.26	3.74	3.86
2.09	2.51	3.88	4.28
3.94	3.80	4.08	4.30
2.27	2.53	2.53	2.70
2.31	2.52	2.19	2.43
3.59	4.02	2.79	2.69
2.43	2.64	2.77	2.53
3.89	4.16	3.15	2.94
3.81	4.26	2.32	2.49
3.73	3.68	3.39	2.73
2.25	2.51	3.60	3.84
2.34	2.42	2.37	2.36
2.45	2.59	2.56	2.33
3.83	3.95	2.06	2.34
3.56	3.92	2.82	2.41
3.64	3.31	2.79	2.79
3.77	4.06	2.99	2.90
2.57	2.58	3.74	3.31
2.80	3.16	3.43	3.87
4.77	4.91	3.48	3.40
3.07	3.47	3.51	3.05
5.27	5.35	2.83	2.74
3.84	4.08	2.25	2.55
3.87	4.21	3.78	4.22
4.34	4.38	3.39	3.53
2.98	3.17	2.42	2.59
3.75	3.93	3.98	4.24
4.48	4.63	4.81	5.19
2.96	3.12	4.22	3.99
3.81	3.97	4.32	5.12
4.53	4.50	3.14	3.12
2.88	3.04	3.07	3.08
2.01	2.51	3.50	3.57
4.42	4.41	2.76	2.58
3.13	3.81	2.84	2.46
4.01	4.14		

4.34	4.36		
5.43	5.40		
4.47	4.71		

Table S17. Experimentally determined and back-calculated (NAMFIS) interproton distances (Å) of PROTACs **6** (-20 ˚C) and **7** (-25 ˚C).

PROTAC **6** (-20 ˚C)		PROTAC **7** (-25 ˚C)	
Exp.	Calc.	Exp.	Calc.
4.20	4.31	3.84	4.11
5.01	4.80	3.75	4.03
3.96	4.45	4.17	4.09
3.25	2.94	4.39	4.30
4.41	4.26	4.56	4.80
2.85	2.78	2.01	2.43
4.70	4.85	4.55	4.72
2.88	2.72	4.00	3.95
3.58	3.77	2.38	2.47
3.02	2.80	2.42	2.68
2.36	2.39	2.84	2.85
3.17	3.42	2.94	2.95
3.98	3.86	4.03	4.15
2.85	2.77	2.65	2.93
2.54	2.82	2.38	2.38
2.77	2.39	3.66	3.53
4.14	4.42	2.33	2.47
3.72	3.89	3.88	3.81
3.70	3.99	2.06	2.34
3.57	3.69	2.12	2.37
2.60	2.39	2.44	2.55
2.48	2.89	2.51	2.70
2.52	2.43	3.10	2.98
2.57	2.91	2.94	3.04
2.48	2.43	3.46	3.27
2.33	2.41	2.35	2.50
2.28	2.41	2.42	2.58
3.11	3.18	2.64	2.82
2.86	3.08	2.86	2.79
5.47	5.83	2.98	3.01
3.55	3.69	2.31	2.42
3.56	3.78	2.42	2.78
3.41	3.54	1.90	2.32
3.17	3.26	3.14	3.39
4.25	5.02	4.22	4.61
3.22	3.84	2.88	2.99

3.15	2.72	4.72	4.45
		3.28	3.56
		4.19	4.29
		3.44	3.53
		2.46	2.77
		4.13	4.56
		4.86	4.74
		2.75	2.90
		2.70	2.95
		4.09	4.20
		4.41	4.69

7. R_{gyr} and SA 3D PSA for the conformational ensembles derived by NAMFIS-analysis for PROTACs 2, 3, 6 and 7

Table S18. Overview of solution conformations and calculated descriptors for each conformation of PROTACs **1**–**2** in the ensembles obtained by NAMFIS analysis.

PROTACs	Conf. No	Popul. (%)	SA 3D PSA (Å^2)[a]	R_{gyr} (Å)[b]
2	1	19	146.0	6.59
	2	15	192.3	5.63
	3	10	145.1	6.71
	4	10	210.0	6.79
	5	8	151.3	6.54
	6	7	149.6	7.2
	7	6	182.0	6.41
	8	6	179.2	6.72
	9	3	174.0	5.96
	10	3	196.3	6.53
	11	2	195.0	6.33
	12	2	190.4	5.64
	13	2	188.9	6.62
	Population weighted mean		***170.9***	***6.46***
3	1	23	218.7	6.78
	2	16	259.2	7.64
	3	15	263.2	5.82
	4	13	199.2	6.37
	5	6	191.5	5.92
	6	5	189.6	5.37
	7	5	207.9	6.47
	8	4	183.6	6.38
	9	4	226.6	6.71
	10	3	190.3	6.18
	11	3	236.2	6.38
	12	2	204.6	6.36
	Population weighted mean		***224.0***	***6.52***
6	1	45	198.4	5.88
	2	18	191.7	7.70
	3	17	228.9	7.63
	4	9	157.1	5.25
	5	8	244.4	10.3
	6	3	193.4	7.20
	Population weighted mean		***206.3***	***6.98***
7	1	23	178.6	7.65
	2	14	152.8	7.52
	3	14	122.1	6.56
	4	12	153.9	6.33
	5	7	168.3	5.69
	6	7	226.6	10.5
	7	5	156.4	7.14
	8	5	176.4	6.19

	9	3	175.0	6.95
	10	2	174.9	5.95
	11	2	191.6	5.49
	12	2	224.1	9.55
	13	2	217.9	8.92
	14	2	194.0	5.28
	Population weighted mean		**167.6**	**7.20**

[a]SA 3D PSA = solvent accessible 3D polar surface area. [b]R_{gyr} = radius of gyration.
